# Porous Supramolecular Crystalline Probe that Detects Non‐Covalent Interactions Involved in Molecular Recognition of Furanic Compounds

**DOI:** 10.1002/smll.202405507

**Published:** 2024-07-30

**Authors:** Shohei Tashiro, Kyohei Kuwabara, Kosei Otsuru, Mitsuhiko Shionoya

**Affiliations:** ^1^ Department of Chemistry Graduate School of Science The University of Tokyo 7‐3‐1 Hongo, Bunkyo‐ku Tokyo 113‐0033 Japan; ^2^ Research Institute for Science and Technology Tokyo University of Science 2641 Yamazaki Noda Chiba 278‐8510 Japan

**Keywords:** biomass, hydrogen bonds, molecular recognition, noncovalent interactions, X‐ray diffraction

## Abstract

Selective separation and conversion of furan‐based biomass‐derived compounds, which are closely related to biorefineries, is currently an important issue. To improve their performance, it is important to deepen the understanding of non‐covalent interactions that act on the molecular recognition of furanic compounds on separation or catalyst matrices. Here, a new method is reported to comprehensively visualize such intermolecular interactions using a porous supramolecular crystalline probe with polar and non‐polar binding sites within a low‐symmetric nanochannel consisting of four isomeric Pd^II^_3_‐macrocycles. Single‐crystal X‐ray diffraction analysis of the crystals including 5‐hydroxymethylfurfural, furfural, furfuryl alcohol, or 2‐acetylfuran reveals a variety of interactions involving their furan rings and polar substituents. It is also found that cooperative and competitive effects between guest and solvent molecules significantly change their recognition mode.

## Graphical abstract

A method is reported for visualizing non‐covalent interactions acting on molecular recognition of furanic compounds with a supramolecular probe, which has polar and non‐polar sites in low‐symmetric cavities. Crystallographic analysis demonstrates that furans are recognized not only through interactions on their polar substituents but also on the furan ring itself.

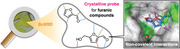

## 1 Introduction

In recent years, in preparation for the depletion of fossil resources, progress has been made in the development of biorefineries that use biomass instead of oil to produce raw materials and chemicals.^[^ ^1^ ^]^ Biomass such as wood are easily converted into several furans, called furanic compounds, which can be further transformed into a variety of useful chemicals. For example, 5‐hydroxymethylfurfural (HMF) is obtained from cellulose‐derived hexoses that make up biomass through a dehydration reaction and is converted into key chemicals such as 2,5‐furandicarboxylic acid and levulinic acid.^[^ ^2^ ^]^ Therefore, furanic compounds are considered as platform compounds in biorefineries, and it is particularly important to develop efficient catalytic conversion and purification methods for them.^[^ ^3^ ^]^

**Figure 1:**
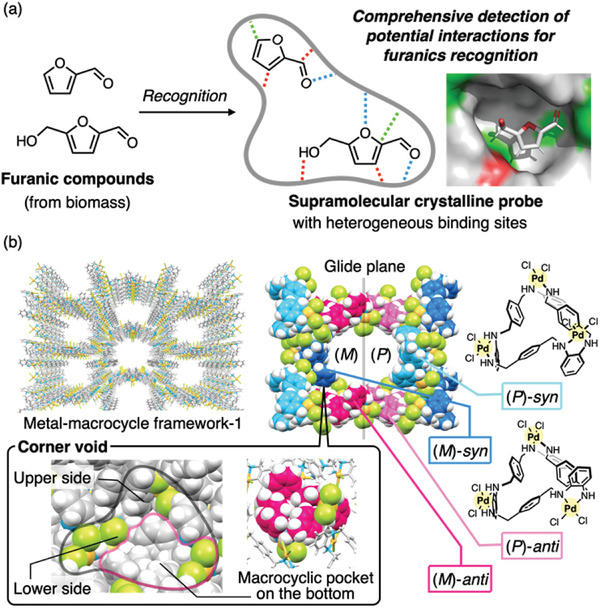
The concept of this study using a porous metal‐macrocycle framework‐1 (MMF‐1). a) Crystallographic detection of non‐covalent interactions affecting molecular recognition of furanic compounds. b) Crystal structures of MMF‐1 formed from (*P*/*M*)‐*syn*‐ and (*P*/*M*)‐*anti*‐Pd^II^_3_‐macrocycles and its corner voids comprising upper, lower sides, and a macrocyclic pocket on the bottom. For Corey‐Pauling‐Koltun (CPK) model, Pd: yellow, Cl: lime, N: light blue, H: white, C: sky blue, blue, pink, and magenta for (*P*)‐*syn*, (*M*)‐*syn*, (*P*)‐*anti*, and (*M*)‐*anti*‐isomers, respectively.

Recently, the use of porous crystalline materials such as zeolites, metal‐organic frameworks (MOFs), and covalent organic frameworks (COFs) as heterogeneous catalysts and separation matrices for furanic compounds has been widely tested.^[^ ^4^, ^5^ ^]^ For example, catalytic Diels‐Alder cycloaddition reactions of furanic compounds have been reported to produce useful aromatic compounds using zeolites or MOFs.^[^ ^5b,f^ ^]^ Selective adsorption of furanic compounds onto MOFs also enabled their separation from aqueous solutions.^[^ ^5c,g^ ^]^ Molecular recognition of furanic compounds is key for such applications, so it is essential to understand the non‐covalent interactions that affect the molecular recognition of furanic compounds during the conversion and purification processes. Crystallographic analysis of furan derivatives entrapped in crystalline host compounds is one of the best ways to experimentally reveal such interactions. For example, unsubstituted furans were introduced as guests in several crystalline hosts, and their host‐guest interactions were analyzed crystallographically.^[^ ^6^ ^]^ In contrast, reported host‐guest crystal structures for biomass‐derived furanic compounds are currently quite limited. Recently, furfural has successfully been encapsulated in porous crystals, but the analysis of intermolecular interactions has mainly focused on its polar substituents.^[^ ^7^ ^]^ To this end, it is also important to understand intermolecular interactions that may be formed by the furan ring itself. More recently, such interactions have been studied using theoretical calculations to improve our understanding of the separation process.^[^ ^5g^ ^]^ However, a comprehensive crystallographic analysis of such interactions using porous crystals remains a challenge due to the lack of suitable host materials with the ability to recognize furan compounds (**Figure** **1**a).

With the recent development of porous crystalline materials, cutting‐edge materials with unique porous structures that can exhibit excellent molecular recognition abilities and space‐specific functions are attracting great attention.^[^ ^8^ ^]^ We have also recently developed porous metal‐macrocycle frameworks (MMFs) formed by the self‐assembly of helically twisted trinuclear Pd^II^‐macrocycles via hydrogen bonds and Pd^II^‐Pd^II^ interactions, and have reported their space‐specific functions.^[^ ^9^ ^]^ Among the MMF series, MMF‐1 is composed of four stereoisomeric Pd^II^‐macrocycles and has nanochannels with low symmetry. Its channel surface features a variety of molecular binding sites, including several macrocycle‐derived inner cavities, aromatic moieties, and polar NH and Pd‐Cl functional groups (Figure 1b and see Figure S1, Supporting Information, for the details). In fact, various organic, organometallic, and bio‐related compounds are site‐selectively arranged on the channel surface as guest molecules.^[^ ^10^ ^]^ Therefore, the unique host structure with a low symmetry distribution of polar and non‐polar binding sites is expected to serve as an excellent probe for comprehensively detecting intermolecular interactions formed by guest molecules. Here, we report the crystallographic visualization of non‐covalent interactions that affect the molecular recognition of furanic compounds using MMF‐1 as a supramolecular crystalline probe (Figure 1). This is one of the new applications of the crystalline sponge method.^[^ ^8f^ ^]^ Examining several furan derivatives as guests revealed that the furan ring itself also plays an important role in host‐guest complexation. Moreover, we also found that these molecular recognition processes were controlled by cooperative and competitive effects between guest and solvent molecules within a confined host framework. This is consistent with our previous findings.^[^ ^10a^ ^]^ These results will lead to the rational design of catalysis and purification processes for furanic compounds.

## 2 Results and Discussion

**Figure 2:**
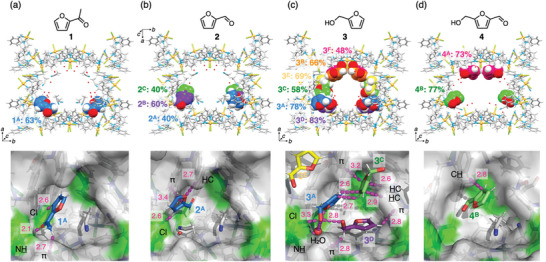
Crystal structures^[^ ^15^ ^]^ of the MMF‐1 nanochannel and its (*M*)‐corner void adsorbing furan derivatives in acetonitrile solution. (a) 2‐Acetylfuran (**1**), (b) furfural (**2**), (c) furfuryl alcohol (**3**), and (d) HMF (**4**). The guest molecules in the nanochannel are represented as CPK models with different colors based on their recognition sites (lower side of the void: blue, macrocyclic pocket on the bottom: purple, upper side of the void: green, lateral macrocyclic pocket: yellow, ceiling H‐bond site: orange, and macrocyclic pocket on the ceiling: red), and the percentage values indicate the chemical occupancy of the guests. In the corner void structures, MMF‐1 and guests are represented as surface and stick models, respectively. The notations such as Cl, NH, and π indicate the counterpart of non‐covalent interactions for the guests shown as magenta dots, and the nearby magenta numbers indicate interatomic distances in Å (see Supporting Information for the details). In the void structure of **2**, disordered guests **2^B^** and **2^C^** are omitted for clarity. The type of the side chain observed in **4^B^** is not specified.

In this study, 2‐acetylfuran (**1**), furfural (**2**), furfuryl alcohol (**3**), 5‐hydroxymethylfurfural (HMF) (**4**), and unsubstituted furan (**5**) were used as guest molecules, among which **2** and **4** are typical furanic compounds derived from lignocellulose. The general procedure we followed was to soak MMF‐1 crystals in an acetonitrile solution of each guest at room temperature and then analyze them by single‐crystal X‐ray diffraction (ScXRD) at −180 °C. As a result, site‐selective guest adsorption onto the channel surface was observed in cases of **1**, **2**, **3**, and **4**, all of which have polar substituents (**Figure** **2**). Their incorporation into MMF‐1 was also confirmed by ^1^H nuclear magnetic resonance (NMR) analysis after dissolving guest@MMF‐1 crystals (Figures S2–S5, Supporting Information). In contrast, no electron distribution attributed to unsubstituted furan **5** in the channel was observed. Based on these results, we next analyzed the binding structure and non‐covalent interactions of each guest in detail.

Figure 2a shows the bonded structure of 2‐acetylfuran (**1**) incorporated into the pores of MMF‐1. **1** is adsorbed to the underside of the void in the bottom corner with 63% occupancy. The position and orientation of **1** are fixed in the void, but the same binding site is also occupied by disordered solvent water and acetonitrile molecules as shown in Figure S7 (Supporting Information) (omitted for clarity in Figure 2a). The main interaction between **1** and MMF‐1 is a classical hydrogen bond between the carbonyl oxygen atom of **1** and the amino proton of MMF‐1. Other non‐covalent interactions are also observed between the furan ring of **1** and the pore surface. For example, the Ar‐H moiety at the 3‐position of the furan ring forms a hydrogen bond with the chloride moiety of MMF‐1.

In contrast, furfural (**2**), which has a formyl group instead of an acetyl group, showed three different binding structures that were disordered in the corner void (Figure 2b). The lower side was occupied by guest **2^A^** (40% occupancy), and the upper side was occupied by guests **2^B^** and **2^C^** (60% and 40% occupancy, respectively), although the formyl groups could not be assigned due to severe disorder. When analyzing the intermolecular interactions of **2^A^**, which shows a clearer electron density, only relatively weak interactions such as O···H‐C, C‐H···Cl‐Pd, and π‐π interactions on the furan ring were observed. However, the polar formyl groups had no significant interactions with the surface. The N‐H site of MMF‐1 that formed a hydrogen bond with the carbonyl group of **1** is instead occupied by an acetonitrile or water molecule. The lack of classical hydrogen bonding may lead to a disordered binding mode of **2** in the corner void. Furthermore, this could explain why the amount of **2** trapped was lower than the other furanic compounds, as shown by ^1^H NMR analysis (Figures S2–S5, Supporting Information).

On the other hand, furfuryl alcohol (**3**) was adsorbed not only to the corner voids but also to the side and ceiling moieties (Figure 2c). In the corner void, guest **3^A^** occupies the lower side with 78% occupancy and forms hydrogen bonds between its hydroxy group and the Pd‐Cl moiety of MMF‐1. In addition, the hydroxy group of **3^A^** also forms hydrogen bonds with the hydroxy group of guest **3^D^** bound to the nearby macrocyclic pocket (83% occupancy) and a co‐adsorbed water molecule at the N‐H site. Furthermore, it was found that the furan ring of **3^A^** formed multipoint CH‐π interactions with that of the guest **3^C^** adsorbed on the upper side (58% occupancy). Considering the intermolecular interactions and occupancies, it is reasonable that the three guests **3^A^**, **3^C^**, and **3^D^** coexist and cooperatively locate in the confined space. Their furan rings also make contact with the pore surfaces via CH‐π and π‐π interactions. In addition to these, guests **3^E^** and **3^F^** adsorb in macrocyclic pockets on the side (69% occupancy) and ceiling (48% occupancy), respectively. The remaining space in the ceiling is also occupied by another guest **3^B^** with 66% occupancy via multipoint hydrogen bonds with the Cl‐Pd and H‐N moieties of MMF‐1. Notably, this is the typical binding mode seen for hydroxymethyl substituents at this site in our previous studies.^[^ ^10a,c,d^ ^]^ There results suggest that furfuryl alcohol (**3**) is efficiently recognized at several binding sites of MMF‐1 through the cooperative effect between the guest and the solvent.

For HMF (**4**), which bears both formyl and hydroxymethyl groups, guests were observed in the corner voids and the ceiling macrocyclic pockets with occupancies of 77% and 73%, respectively (Figure 2d). In the corner void, **4^B^** occupies the upper side through CH‐π interactions, and the nearby macrocyclic pocket adsorbs one acetonitrile molecule, similar to the cases of **1** and **2**. On the ceiling, CH‐π and C‐H···Cl‐Pd interactions were detected on the furan ring of **4^A^** (Figure S32, Supporting Information), but unlike the cases of **1** and **3**, no classical hydrogen bonds were found on the polar substituents.

Comparing the binding structures of four furan derivatives to MMF‐1, we found that the bottom corner voids served as an excellent molecular recognition site. This result is consistent with the trend in our previous studies that several aromatic guests with an elliptical shape, which also applies to guests **1**–**4**, fit the shape of the cavity and bind preferentially to the corner voids.^[^ ^9^ ^]^ On the other hand, the binding mode of each guest to the void is significantly different from each other (Figure 2), which is probably due to the differences in the electronic and steric properties of the substituents. In addition to the corner voids, several macrocyclic pockets at the bottoms, sides, and ceiling of MMF‐1 also tend to bind furan derivatives such as **3** and **4** (Figure S50, Supporting Information). These results suggest that furan derivatives bearing polar substituents show high affinity for channel surfaces bearing several functionalities, but the substituents do not necessarily interact directly with the surface.

**Figure 3:**
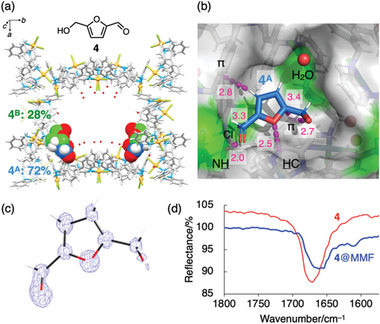
Adsorption of HMF (**4**) onto MMF‐1 in aqueous solution. a,b) The crystal structure^[^ ^15^ ^]^ of the MMF‐1 nanochannel and **4** adsorbed in its (*M*)‐corner void. Representation of the molecules, colors, and notations are identical to those of Figure 2. In the void structure, disordered guest **4^B^** is omitted for clarity. c) Electron density map of **4^A^** in the corner void (contour level: 1.0σ). d) Magnified IR spectra of **4** (red line) and MMF‐1 crystals adsorbed with **4** (blue line).

The solvent effect on the recognition of furanic compounds was also examined by soaking MMF‐1 in aqueous or chloroform solutions of HMF (**4**) with versatile solubility. Crystal structure analysis of MMF‐1 after soaking in an aqueous solution of **4** revealed that **4** adsorbs exclusively to the corner voids and is disordered between two distinct binding modes **4^A^** and **4^B^** in a ratio of 72:28 (**Figure** **3**a). In particular, the major part **4^A^** showed a very distinct electron density distribution by forming a variety of non‐covalent interactions with MMF‐1 (Figure 3b,c). For example, the oxygen and carbon atoms of the formyl group interact with the H‐N and Cl‐Pd moieties of MMF‐1, respectively. In addition, the furan ring is recognized through π‐π, CH‐π, and O···H‐C interactions. The presence of classical hydrogen bonds involving the formyl group was also confirmed by Fourier transform infrared (FT‐IR) spectroscopy, which showed a significant red‐shift of the C═O stretching band (Figure 3d). In comparison, ScXRD analysis of MMF‐1 soaked in a chloroform solution of **4** also gave a bonded structure (62% occupancy) in the corner voids, which was similar to that of **4^A^** obtained in aqueous solution (Figure S49, Supporting Information). In contrast to the aqueous solution, the electron density was less clear than that observed for **4^A^** in aqueous solution, and therefore the orientation of the guest in chloroform could not be determined (Figure S46, Supporting Information).

As mentioned above, the binding structures of **4** in water or chloroform are very similar to each other, so it can be speculated that the binding structure of **4^A^** in water is one of the most stable host‐guest structures at this binding site. In contrast, **4** in acetonitrile exhibited a significantly different binding structure (Figure 2d). A possible reason for the different binding mode observed in acetonitrile is that the acetonitrile molecule bound to the macrocyclic pocket on the bottom sterically inhibits the formation of the stable binding structure observed in water. As shown in the cases of **1** and **2**, in MMF‐1, such a competitive effect may emerge because this macrocyclic pocket tends to capture an acetonitrile molecule (Figure 2a,b).

**Figure 4:**
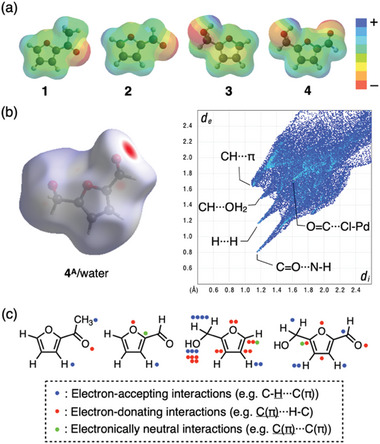
Binding modes of the furan derivatives in MMF‐1. a) Electrostatic potential maps of **1**, **2**, **3**, and **4** created by density functional theory (DFT) calculations at the B3LYP/6‐31G* level. b) Hirshfeld surface, mapped with *d*_norm_, of **4^A^** in the corner void of MMF‐1 soaked in aqueous solution and its 2D fingerprint plot. The *d_i_* and *d_e_* values are the closest internal and external distances from given points on the Hirshfeld surface. c) The total count of non‐covalent interactions for each guest detected by the Hirshfeld surface analyses. Ambiguous interactions caused by structural disorder are essentially not included in this count.

Based on the above crystal structure analyses, we comprehensively analyzed the effective non‐covalent interactions involved in the molecular recognition of furan derivatives with different electronic properties in MMF‐1 (**Figure** **4**). Specifically, we counted all interactions detected by the Hirshfeld surface analysis^[^ ^11^ ^]^ excluding unassignable interactions caused by disorder, and classified the counted interactions into electron‐accepting, electron‐donating, and electronically neutral interactions (Figure 4b,c and Supporting Information). A notable conclusion drawn from this analysis is that in addition to the polar substituents, the furan ring itself also plays an important role in the molecular recognition of furan derivatives. For example, the aromatic π‐faces of **3** and **4** tend to exhibit CH‐π interactions with another H‐Ar moieties, which is consistent with the relatively electron‐donating nature of the hydroxymethyl substituent. On the other hand, regardless of the type of substituents, the Ar‐H moiety of the furan rings at the 3‐ or 4‐position often forms a CH‐π interactions, probably due to the less sterically hindered environment. In addition to the carbon and hydrogen atoms, the oxygen atom in the furan ring also forms weak hydrogen bonds with other components in some cases. These results suggest that such weaker non‐covalent interactions acting cooperatively on the furan ring, in addition to the stronger interactions formed by polar substituents, are one of the important factors controlling the molecular recognition mode of furanic compounds.

It is worth noting that these results also provide new insights into statistical data analysis based on the Cambridge Structural Database. Full Interaction Maps analysis, based on functional group interaction data accumulated in crystallographic databases,^[^ ^12^ ^]^ mainly focuses on hydrogen bonds on the polar substituents, with weak interactions on the furan ring considered to be unimportant and ineffective (Figure S51, Supporting Information). However, the actual experiments in this study demonstrated that these small interactions are cooperative and cannot be ignored. This finding is consistent with the fact that weak non‐covalent interactions often act cooperatively in supramolecular chemistry.^[^ ^13^ ^]^ Therefore, the crystalline probe method presented here represents a useful method for experimental detection of minor but important interactions even when there are not enough data in the database, as in the case of the furanic compounds mentioned in the introduction.

Finally, to support our findings, we tested whether MMF‐1 could be used to separate furanic compounds from a mixture based on molecular recognition. According to the literature,^[^ ^14^ ^]^ cellulose was first decomposed in water by microwave heating, and its filtrate was concentrated to prepare an aqueous mixture composed of HMF (**4**) and many other byproducts, such as sugars. MMF‐1 crystals were then soaked in the mixture for 3 h at 30 °C, and the compounds adsorbed in the crystals were extracted into D_2_O. When the extract was analyzed by ^1^H NMR, only **4** was observed, without any byproducts, suggesting that **4** was selectively separated from the complex mixture of decomposed cellulose by MMF‐1 (Figure S52, Supporting Information). The efficient adsorption of **4** even from aqueous solution is probably due to the cooperative action of CH‐π and CH‐O multipoint interactions in the furan ring in addition to hydrogen bonding at the polar substituents. Therefore, the use of water as the separation solvent is a suitable condition for MMF‐1, which is consistent with a previous report on the separation of **4** from an aqueous mixture using a hydrophobic MOF.^[^ ^5c^ ^]^

## 3 Conclusion

In conclusion, non‐covalent interactions formed by several furan derivatives, such as cellulose‐derived furfural and HMF, were crystallographically visualized using porous MMF‐1 with low‐symmetric binding cavities within the nanochannels. Comprehensive analysis of the detected interactions revealed that non‐covalent interactions such as CH‐π, π‐π, C‐H···Cl‐Pd, and O···H‐C interactions on the furan ring contribute significantly to the molecular recognition of furanic compounds. It is noteworthy that molecular recognition is often mediated by such weak and cooperative interactions of the furan ring as well as hydrogen bonding of polar substituents. We also found that cooperative and competitive effects between guests and solvents control their molecular recognition mode, as seen in the solvent‐dependent binding of HMF and the cooperative arrangement of furfuryl alcohol in the corner void. These findings on the molecular recognition of furanic compounds, combined with other analytical methods, will provide useful guidance for the future design of catalysts and separation matrices for furan‐based biorefineries.

## Conflict of Interest

The authors declare no conflict of interest.

## Supporting information

Supporting Information

Supporting Information

## Data Availability

The data that support the findings of this study are available in the supplementary material of this article.
