# Supplementary material for: Porous Supramolecular Crystalline Probe that Detects Non‐Covalent Interactions Involved in Molecular Recognition of Furanic Compounds
Source: Small. 2024 Jul 30;20(49):2405507. doi: 10.1002/smll.202405507 (PMC11618713; doi:10.1002/smll.202405507)
Supplement: Supplementary file 1 — Supporting Information [file SMLL-20-2405507-s002.pdf]

NANO · MICRO
small

Supporting Information

for *Small*, DOI 10.1002/smll.202405507

Porous Supramolecular Crystalline Probe that Detects Non-Covalent Interactions Involved in Molecular Recognition of Furanic Compounds

Shohei Tashiro, Kyohei Kuwabara, Kosei Otsuru and Mitsuhiko Shionoya**

Supporting Information

Porous Supramolecular Crystalline Probe that Detects Non-Covalent Interactions Involved in Molecular Recognition of Furanic Compounds

Shohei Tashiro,[a] Kyohei Kuwabara,[a] Kosei Otsuru,[a] and Mitsuhiko Shionoya[a,b]

[a]*Department of Chemistry, Graduate School of Science, The University of Tokyo, 7-3-1 Hongo, Bunkyo-ku, Tokyo 113-0033, Japan*

[b]*Research Institute for Science and Technology, Tokyo University of Science, 2641 Yamazaki, Noda, Chiba 278-8510, Japan*

• **Materials and methods** page S2

• **Details of the structure of MMF-1** page S3

• **Confirmation of guest inclusion in MMF-1 by ^{1}H NMR analysis** page S4

• **Crystallographic observation of the adsorption structure of furan derivatives** page S6

*2-Acetylfuran (**1**) in acetonitrile* page S6

*Furfural (**2**) in acetonitrile* page S8

*Furfuryl alcohol (**3**) in acetonitrile* page S12

*5-Hydroxymethylfurfural (**4**) in acetonitrile* page S19

*Furan (**5**) in acetonitrile* page S23

*5-Hydroxymethylfurfural (**4**) in water* page S24

*5-Hydroxymethylfurfural (**4**) in chloroform* page S28

• **Full Interaction Maps analysis of furanic compounds** page S32

• **Separation of HMF from decomposed cellulose using MMF-1** page S33

• **References** page S34

Materials and methods

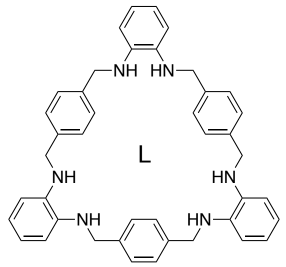

MMF-1 crystals were prepared according to our previous report with macrocyclic ligand L and stored in acetonitrile until use.[1] Other solvents, organic and inorganic reagents are commercially available, and were used without further purification.

Single-crystal X-ray diffraction (ScXRD) analyses were performed using a Rigaku XtaLAB P200 diffractometer with CuKα radiation, and the obtained data were analyzed using the Olex2 crystallographic software package[2] except for refinement, which was performed using the SHELXL-2015 program suite.[3] Hydrogen atoms were placed at the calculated positions with AFIX instructions and refined using a riding model. Several restraints (DFIX, FLAT, RIGU, DELU and ISOR) were applied to MMF, bound guests and solvent molecules to avoid the collapse of the structures during the least-squares refinement due to the large anisotropic displacement parameters. The large parameters of some parts of MMF-1 are derived from its structural flexibility with partial disorder, and those of the guest and solvent molecules are derived from the large thermal vibration arising from weak noncovalent interactions between the trapped molecules and the interior MMF-1 surface. Therefore, relatively large *R*-values sometimes obtained are inherent nature of MMF-1, and it is often improved by applying SQUEEZE or solvent mask functions, though they were not applied in this work as described below. Still, the presence and the positions (orientations) of guest molecules are justified based on the electron density map which are adequate for the conclusion on this work. The SQUEEZE and solvent mask functions that remove ambiguous electrons from the large void were not used in this work to avoid any effect on the electron density of guest molecules trapped on the pore surfaces. Alternatively, relatively weak electron densities in the pore, especially around guests, were assigned into solvent molecules as much as possible to correctly discuss the guest's structures, although the details of such solvents with weaker electron density were not discussed in this study. X-ray structures were displayed using the Mercury[4] or PyMOL[5] programs. The electron density of the guests was displayed using the ShelXle program.[6] Hirshfeld surface analysis was conducted using the CrystalExplorer program (Version 17.5).[7] Intermolecular interaction distances shown in the SI are calculated by the CrystalExplorer program.

NMR spectroscopic measurements were performed using a Bruker AVANCE 500 spectrometer (500 MHz for ^{1}H), and the chemical shifts were reported in parts per million (ppm). FT-IR spectra were recorded on a JASCO FT/IR-4200 spectrometer using a ZnSe attenuated total reflection (ATR) method. Microwave heating was conducted using an Anton Paar Monowave 300 microwave reactor with continuous stirring at 1000 rpm. The reaction temperature was monitored using an IR sensor.

Electrostatic potential map of **1**, **2**, **3**, and **4** were created by DFT calculation at the B3LYP/6-31G* level in gas state, which was conducted with the Spartan 16 program.[8]

Details of the structure of MMF-1

The details of MMF-1 are summarized in our earlier papers,[1] but the details and the structure of each binding site are also presented in this section to aid understanding of this manuscript.

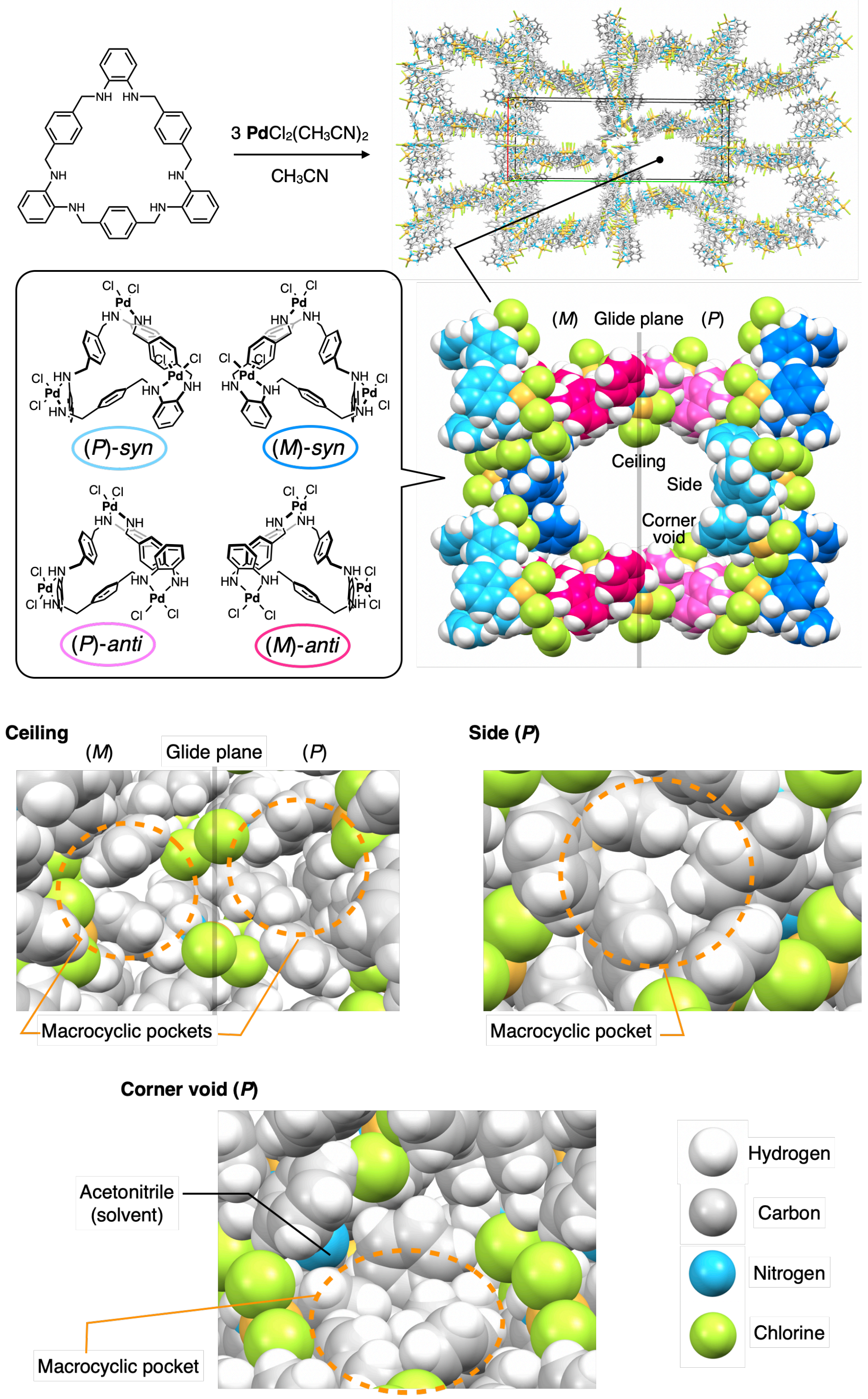

Figure S1. The formation and the crystal structure of MMF-1 composed of four isomeric Pd_3-macrocycles (top), and the detailed structure of each binding site (bottom).

Confirmation of guest inclusion in MMF-1 by ^{1}H NMR analysis

For the purposes of this analysis, the "unit-space" of MMF-1 is defined as half of the unit cell of each crystal structure. Thus, the unit-space corresponds to one unit of nanochannels which contains four molecules of L.

*2-Acetylfuran (**1**)*

MMF-1 crystals were soaked in an acetonitrile solution of **1** (1.0 M) for 15 min at room temperature. The crystals were collected by filtration, washed with a small amount of acetonitrile, and then dissolved in DMSO-d_6 with DCl/D_2O to measure ^{1}H NMR spectroscopy.

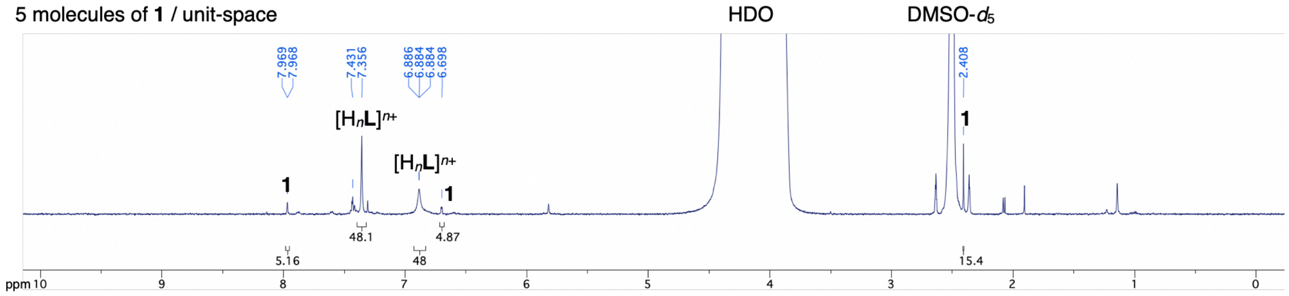

Figure S2. ^{1}H NMR spectrum (500 MHz, DMSO-d_6, 300 K) after dissolving **1**@MMF-1 crystals in DMSO-d_6/DCl-D_2O.

*Furfural (**2**)*

MMF-1 crystals were soaked in an acetonitrile solution of **2** (1.0 M) for 30 min at room temperature. The crystals were collected by filtration, washed with a small amount of acetonitrile, and then dissolved in DMSO-d_6 with DCl/D_2O to measure ^{1}H NMR spectroscopy.

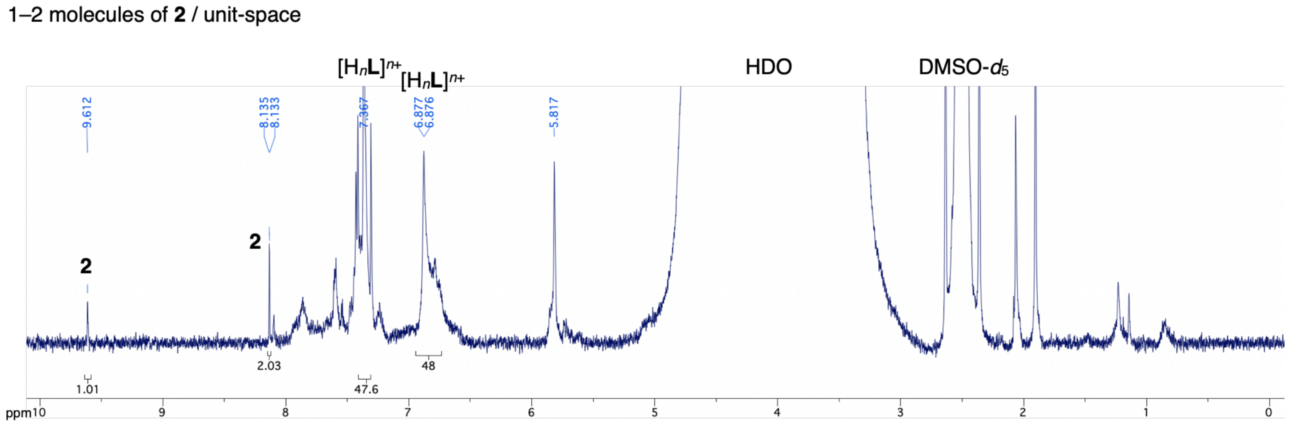

Figure S3. ^{1}H NMR spectrum (500 MHz, DMSO-d_6, 300 K) after dissolving **2**@MMF-1 crystals in DMSO-d_6/DCl-D_2O.

*Furfuryl alcohol (**3**)*

MMF-1 crystals were soaked in an acetonitrile solution of **3** (1.0 M) for 15 min at room temperature. The crystals were collected by filtration, washed with a small amount of acetonitrile, and then dissolved in DMSO-d_6 with DCl/D_2O to measure ^{1}H NMR spectroscopy.

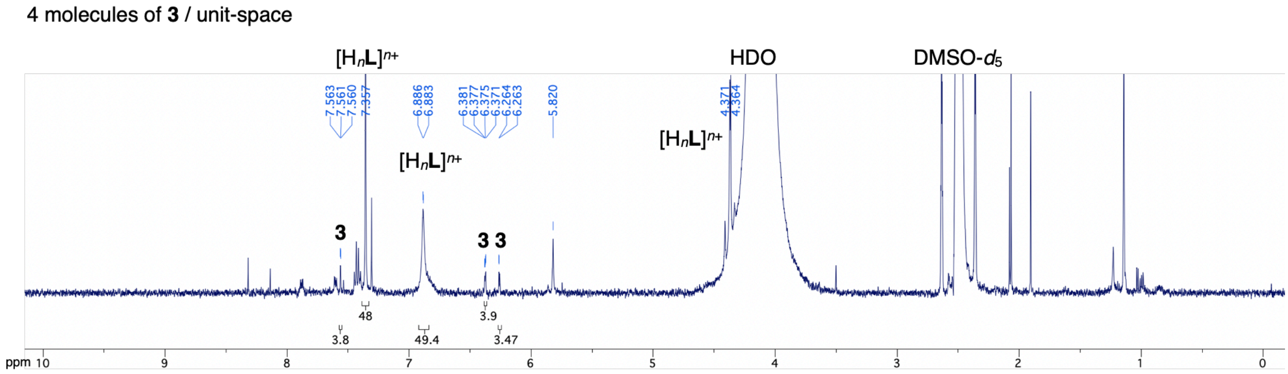

Figure S4. ^{1}H NMR spectrum (500 MHz, DMSO-d_6, 300 K) after dissolving **3**@MMF-1 crystals in DMSO-d_6/DCl-D_2O.

*5-Hydroxymethylfurfural (**4**)*

MMF-1 crystals were soaked in an acetonitrile solution of **4** (1.0 M) for 20 min at room temperature. The crystals were collected by filtration, washed with a small amount of acetonitrile, and then dissolved in DMSO-d_6 with DCl/D_2O to measure ^{1}H NMR spectroscopy.

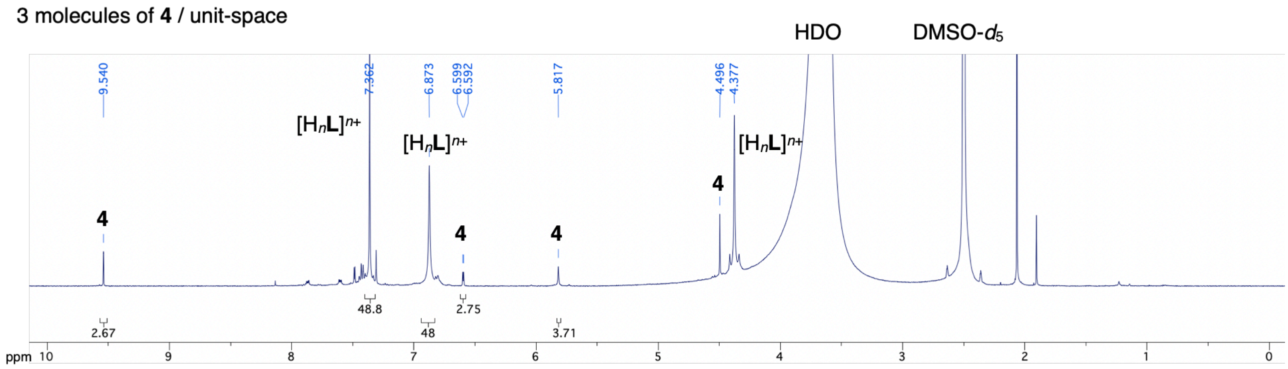

Figure S5. ^{1}H NMR spectrum (500 MHz, DMSO-d_6, 300 K) after dissolving **4**@MMF-1 crystals in DMSO-d_6/DCl-D_2O.

Crystallographic observation of the adsorption structure of furan derivatives

*2-Acetylfuran (**1**) in acetonitrile*

MMF-1 crystals were soaked in an acetonitrile solution of **1** (1.0 M) for 18 min at room temperature. The crystals were taken out on a glass plate, mixed with Paratone oil, and then analyzed by single-crystal X-ray diffraction at –180 °C.

Crystal data for $[Pd_3LCl_6]_2\cdot(\mathbf{1})_{0.632}\cdot(CH_3CN)_{3.535}\cdot(H_2O)_{4.452}$: $C_{94.86}H_{98.39}Cl_{12}N_{15.53}O_{5.71}Pd_6$, F_w = 2611.33, crystal dimensions 0.252 × 0.186 × 0.109 mm^3, monoclinic, space group $P2_1/c$, a = 19.57780(15), b = 52.1918(5), c = 14.27570(8) Å, β = 90.5101(6)°, V = 14586.3(2) Å^3, Z = 4, ρ_{calcd} = 1.189 g cm^{-3}, μ = 8.202 cm^{-1}, T = 93 K, λ(CuKα) = 1.54184 Å, $2\theta_{max}$ = 136.496°, 149590/26651 reflections collected/unique (R_{int} = 0.0977), R_1 = 0.1166 ($I > 2\sigma(I)$), wR_2 = 0.3135 (for all data), GOF = 1.095, largest diff. peak and hole 4.354/–2.437 eÅ^{-3}. CCDC deposit number 2252159.

The occupancy of the guest molecules was refined using free variables. The orientation of the furan ring was determined based on the repulsion between C-H or O of the furan ring and the MMF framework. If the orientation is reversed, the furan oxygen atom may cause electrostatic repulsion with a chloride ligand, and electrostatically favorable C-H···Cl hydrogen bonds is lost. The occupancy of solvent molecules was set to be 1, 0.75 0.5, 0.33, or 0.25 based on the *Ueq* value except for disordered solvents whose occupancies were refined using free variables. Hydrogen atoms of water molecules could not be located in the difference electron density maps.

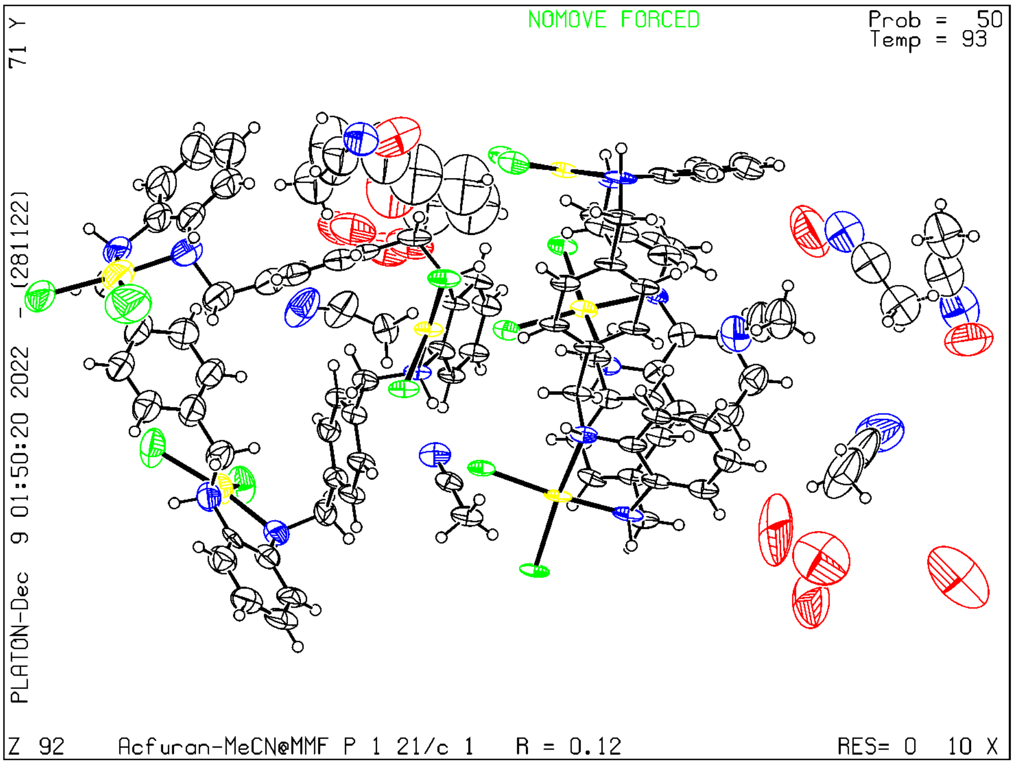

Figure S6. ORTEP drawing of the asymmetric unit of **1**@MMF-1 at the 50% probability level. Color: C grey, N blue, O red, Cl green, and Pd yellow.

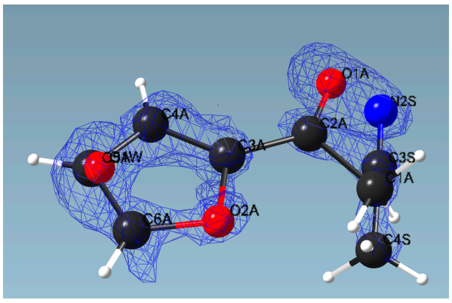

Figure S7. Electron density map of **1** and disordered solvents bound at the lower side of a bottom corner void (contour level: 0.63σ).

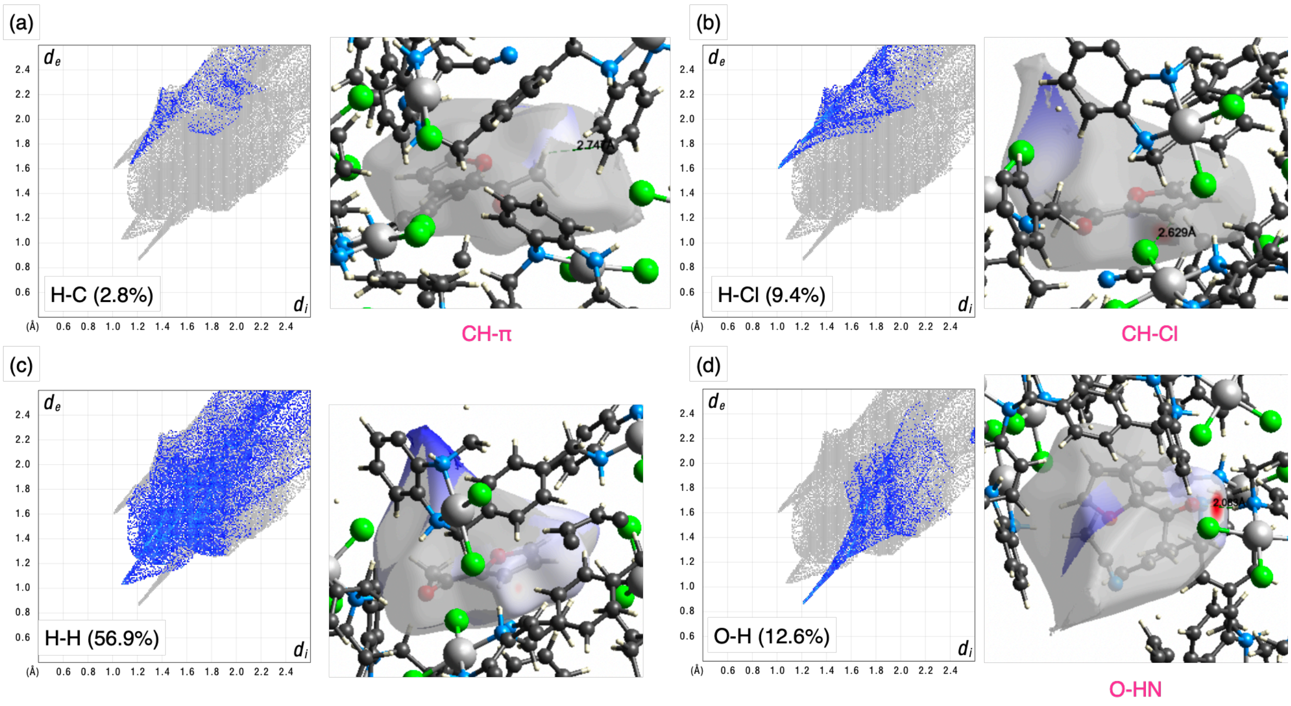

Figure S8. Three-dimensional Hirshfeld surfaces of **1** plotted over d_{norm} in the range –0.4005 to 5.6346 a.u. and fingerprint plots for the Hirshfeld surface. The percentage values indicate the contribution of the interactions to the Hirshfeld surface, and the d_i and d_e values are the closest internal and external distances (Å) from given points on the Hirshfeld surface. Fingerprint plots between (a) hydrogen and carbon, (b) hydrogen and chlorine, (c) hydrogen and hydrogen, and (d) oxygen and hydrogen atoms of **1** and other moieties including MMF-1, respectively.

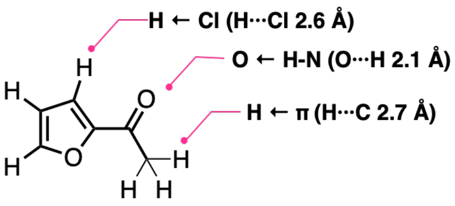

Figure S9. Summary of non-covalent interactions of **1** at MMF-1 in acetonitrile.

Furfural (2) in acetonitrile

MMF-1 crystals were soaked in an acetonitrile solution of **2** (1.0 M) for 1 h at room temperature. The crystals were taken out on a glass plate, mixed with Paratone oil, and then analyzed by single-crystal X-ray diffraction at –180 °C.

Crystal data for $[Pd_3LCl_6]_2\cdot(\mathbf{2})_{1.395}\cdot(CH_3CN)_{4.664}\cdot(H_2O)_{3.419}$: $C_{100.91}H_{100.18}Cl_{12}N_{16.66}O_{4.82}Pd_6$, F_w = 2687.19, crystal dimensions 0.295 × 0.209 × 0.042 mm^3, monoclinic, space group $P2_1/c$, a = 19.57830(15), b = 52.1082(5), c = 14.30530(9) Å, β = 90.8158(6)°, V = 14592.6(2) Å^3, Z = 4, ρ_{calcd} = 1.223 g cm^{-3}, μ = 8.211 cm^{-1}, T = 93 K, λ(CuKα) = 1.54184 Å, $2\theta_{max}$ = 136.498°, 150716/26697 reflections collected/unique (R_{int} = 0.0573), R_1 = 0.1016 ($I > 2\sigma(I)$), wR_2 = 0.2665 (for all data), GOF = 1.137, largest diff. peak and hole 2.665/–2.895 eÅ^{-3}. CCDC deposit number 2252160.

The occupancy of the guest molecules was refined using free variables. The structure of two of the three guests was partially assigned due to severe disorder. The orientation of the furan ring of them was not determined, so all the atoms of the five-membered rings were assigned as carbon atoms and their sp^2 hydrogen atoms were not assigned. In addition, the side chain of the third guest could not be assigned based on the electron density. The occupancy of disordered solvent molecules with the guests were refined using free variables, and that of other solvents were set to be 1, 0.75 0.5, 0.33, or 0.25 based on the *Ueq* value. Hydrogen atoms of water molecules could not be located in the difference electron density maps.

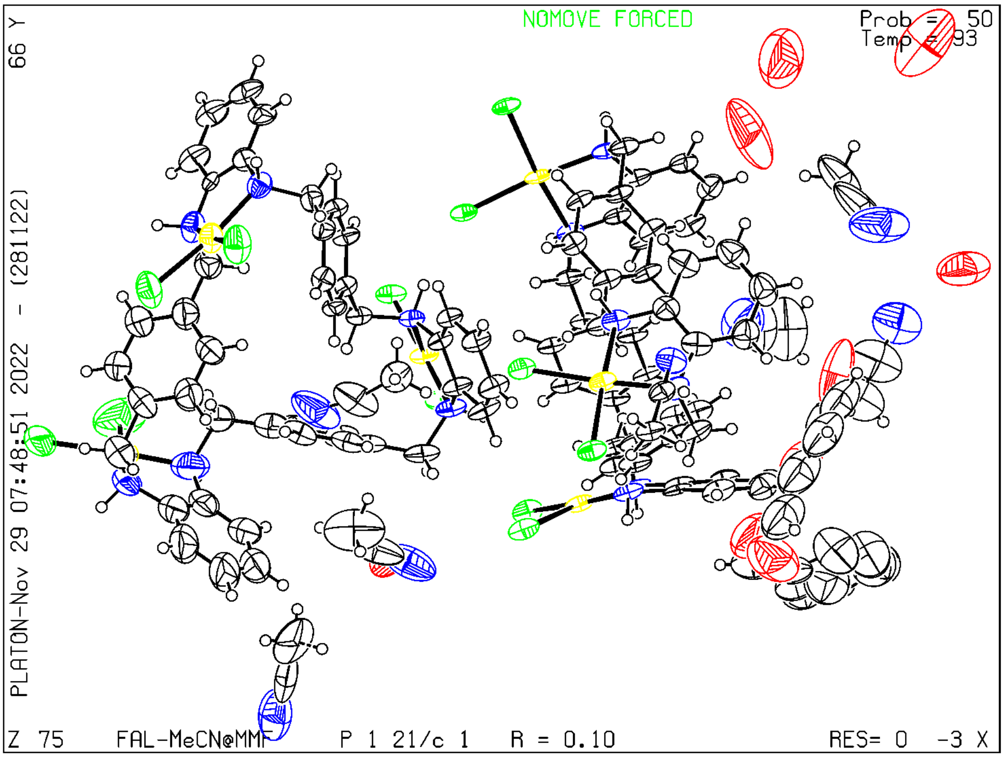

Figure S10. ORTEP drawing of the asymmetric unit of **2**@MMF-1 at the 50% probability level. Color: C grey, N blue, O red, Cl green, and Pd yellow.

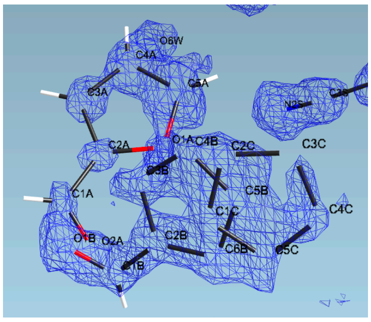

Figure S11. Electron density map of **2** disordered at three positions and acetonitrile bound at a bottom corner void (contour level: 0.63σ).

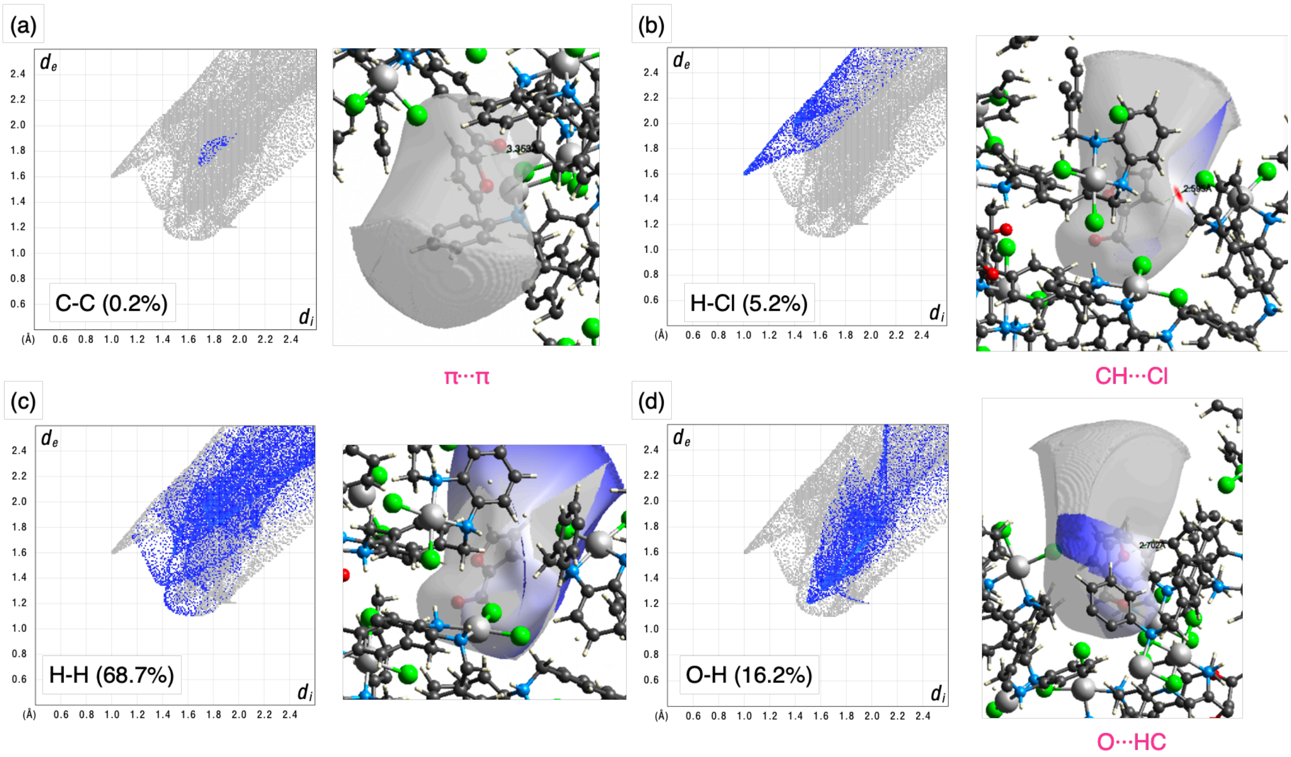

Figure S12. Three-dimensional Hirshfeld surfaces of $\mathbf{2}^{A}$ plotted over d_{norm} in the range –0.1693 to 6.6483 a.u. and fingerprint plots for the Hirshfeld surface. The percentage values indicate the contribution of the interactions to the Hirshfeld surface, and the d_i and d_e values are the closest internal and external distances (Å) from given points on the Hirshfeld surface. Fingerprint plots between (a) carbon and carbon, (b) hydrogen and chlorine, (c) hydrogen and hydrogen, and (d) oxygen and hydrogen atoms of $\mathbf{2}^{A}$ and other moieties including MMF-1, respectively.

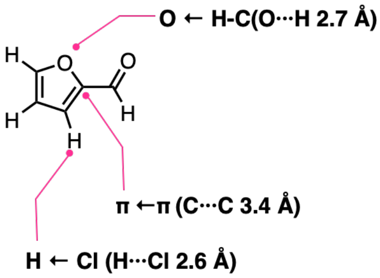

Figure S13. Summary of non-covalent interactions of $\mathbf{2}^{A}$ at MMF-1 in acetonitrile.

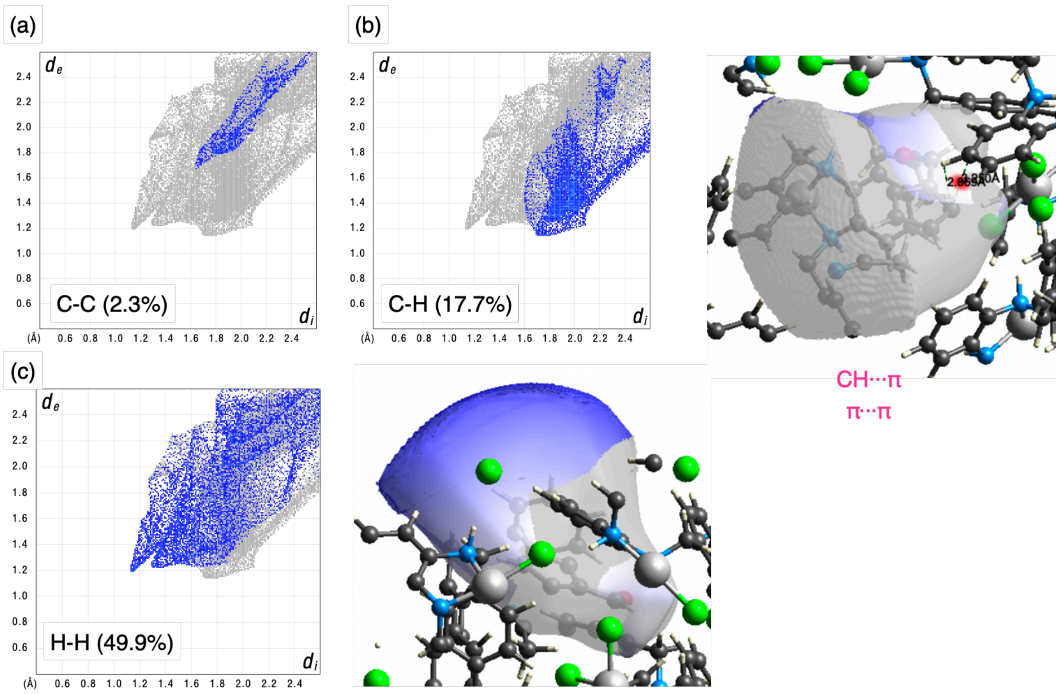

Figure S14. Three-dimensional Hirshfeld surfaces of **2**$^{\mathbf{B}}$ plotted over d_{norm} in the range –0.0871 to 6.7238 a.u. and fingerprint plots for the Hirshfeld surface. The percentage values indicate the contribution of the interactions to the Hirshfeld surface, and the d_i and d_e values are the closest internal and external distances (Å) from given points on the Hirshfeld surface. Fingerprint plots between (a) carbon and carbon, (b) carbon and hydrogen, and (c) hydrogen and hydrogen atoms of **2**$^{\mathbf{B}}$ and other moieties including MMF-1, respectively. Note that the orientation of this furan ring could not be determined.

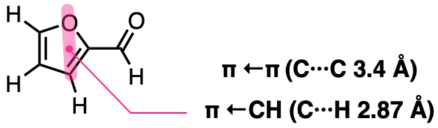

Figure S15. Summary of non-covalent interactions of **2**$^{\mathbf{B}}$ at MMF-1 in acetonitrile. The ambiguous interactions caused by disorder are not included in the count.

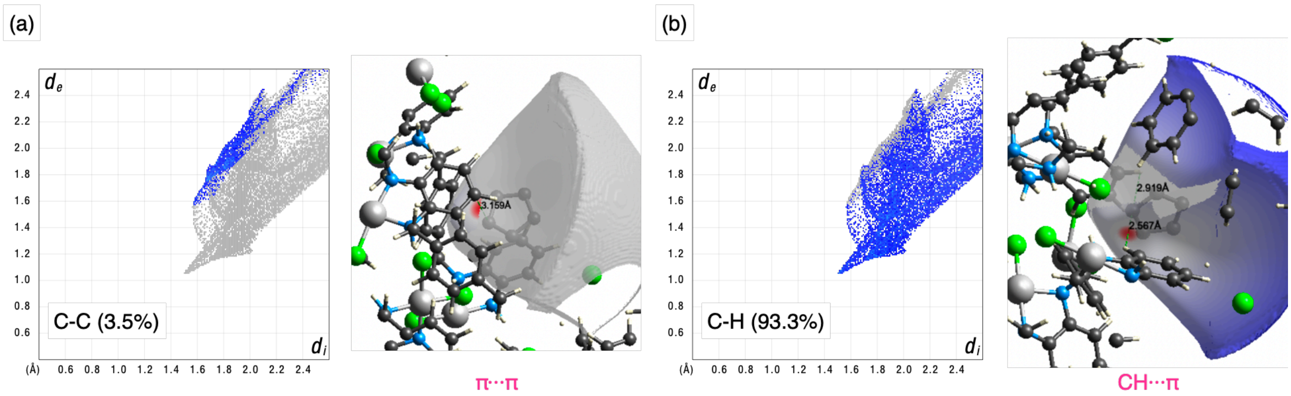

Figure S16. Three-dimensional Hirshfeld surfaces of **2**^{C} plotted over d_{norm} in the range –0.1429 to 7.0411 a.u. and fingerprint plots for the Hirshfeld surface. The percentage values indicate the contribution of the interactions to the Hirshfeld surface, and the d_i and d_e values are the closest internal and external distances (Å) from given points on the Hirshfeld surface. Fingerprint plots between (a) carbon and carbon, and (b) carbon and hydrogen atoms of **2**^{C} and other moieties including MMF-1, respectively. Note that the orientation of this furan ring and the position of the formyl group could not be determined.

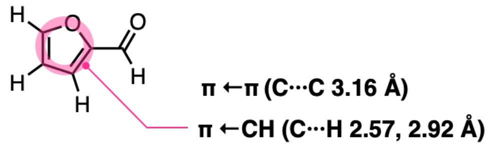

Figure S17. Summary of non-covalent interactions of **2**^{C} at MMF-1 in acetonitrile. The ambiguous interactions caused by disorder are not included in the count.

*Furfuryl alcohol (**3**) in acetonitrile*

MMF-1 crystals were soaked in an acetonitrile solution of **3** (1.0 M) for 20 min at room temperature. The crystals were taken out on a glass plate, mixed with Paratone oil, and then analyzed by single-crystal X-ray diffraction at –180 °C.

Crystal data for $[Pd_3LCl_6]_2\cdot(\mathbf{3})_{4.016}\cdot(CH_3CN)_{1.0}\cdot(H_2O)_{4.727}$: $C_{106.08}H_{104.66}Cl_{12}N_{13}O_{11.79}Pd_6$, F_w = 2814.12, crystal dimensions 0.326 × 0.173 × 0.069 mm^3, monoclinic, space group $P2_1/c$, a = 19.61990(12), b = 52.2723(4), c = 14.30240(9) Å, β = 91.5063(6)°, V = 14663.15(17) Å^3, Z = 4, ρ_{calcd} = 1.275 g cm^{-3}, μ = 8.225 cm^{-1}, T = 93 K, λ(CuKα) = 1.54184 Å, $2\theta_{max}$ = 136.498°, 155301/26837 reflections collected/unique (R_{int} = 0.1195), R_1 = 0.1011 ($I > 2\sigma(I)$), wR_2 = 0.2821 (for all data), GOF = 1.093, largest diff. peak and hole 3.515/–1.427 eÅ^{-3}. CCDC deposit number 2252161.

The occupancy of the guest molecules was refined using free variables. The structure of one guest molecule was partially assigned due to severe disorder, and its O and H atoms and side-chain could not be determined. Two guest molecules were assigned as a disordered model of two possible orientations of the furan ring. The orientation of the furan ring of the remaining guests was determined based on the contact manner with the MMF framework. Specifically, steric and/or electrostatic repulsion with the framework were taken into account for the determination of the orientation. The occupancy of solvent molecules disordered with the guests were refined using free variables, and that of other solvents were set to be 1, 0.75 0.5, 0.33, or 0.25 based on the *Ueq* value. Hydrogen atoms of water molecules and hydroxy groups could not be located in the difference electron density maps.

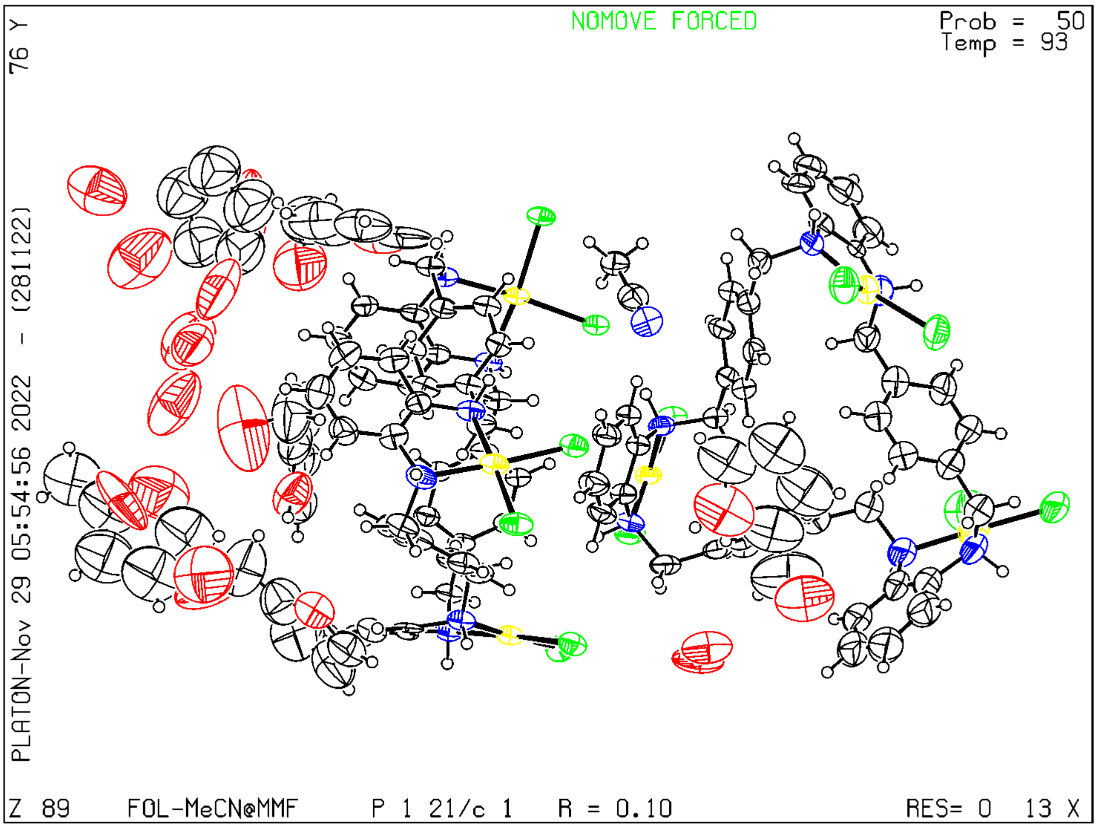

Figure S18. ORTEP drawing of the asymmetric unit of **3**@MMF-1 at the 50% probability level. Color: C grey, N blue, O red, Cl green, and Pd yellow.

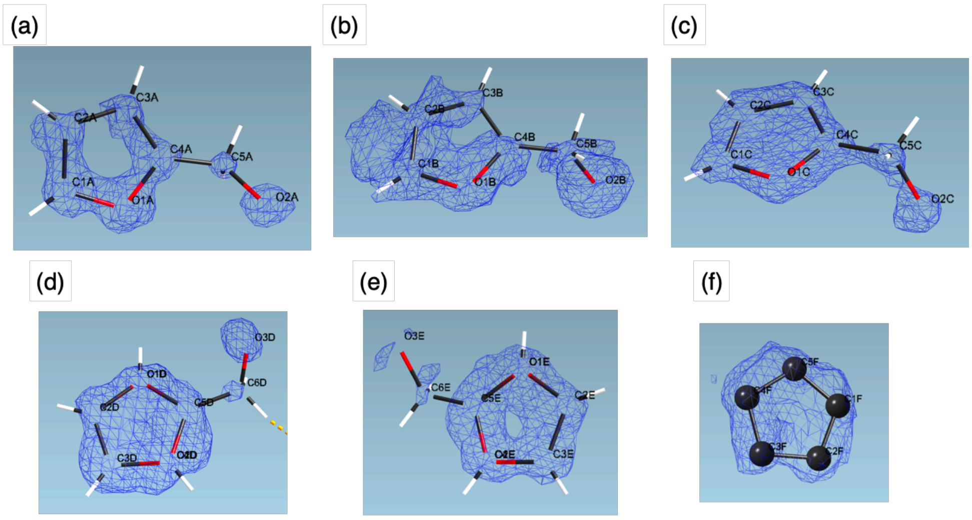

Figure S19. Electron density map of (a) $\mathbf{3}^{\mathbf{A}}$ at the lower side of a bottom corner void (contour level: 0.86σ), (b) $\mathbf{3}^{\mathbf{B}}$ at the ceiling (contour level: 0.58σ), (c) $\mathbf{3}^{\mathbf{C}}$ at the upper side of a bottom corner void (contour level: 0.84σ), (d) $\mathbf{3}^{\mathbf{D}}$ at a macrocyclic pocket on the bottom (contour level: 0.71σ), (e) $\mathbf{3}^{\mathbf{E}}$ at a lateral macrocyclic pocket (contour level: 0.79σ), and (f) $\mathbf{3}^{\mathbf{F}}$ at a macrocyclic pocket on the ceiling (contour level: 0.53σ).

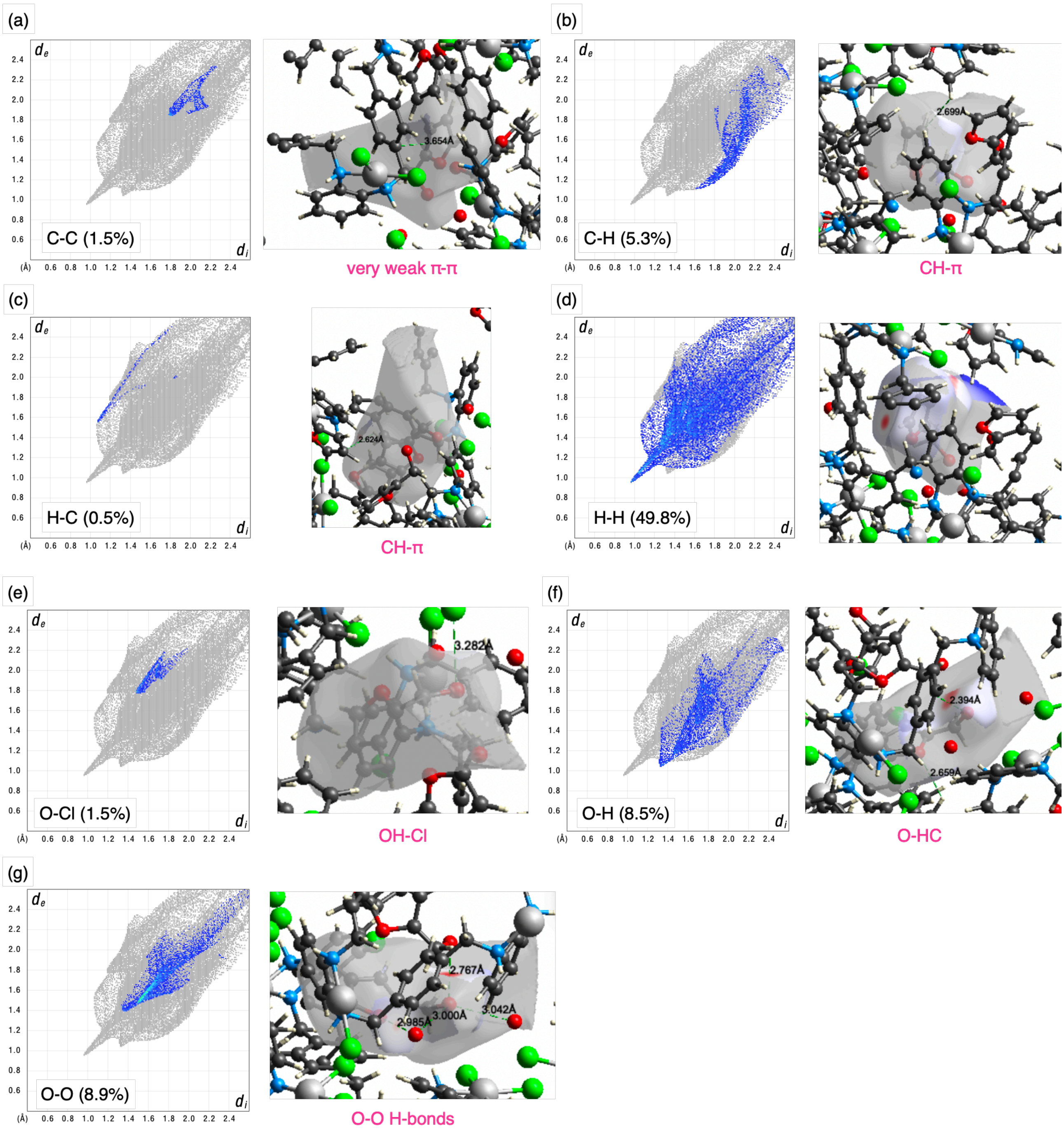

Figure S20. Three-dimensional Hirshfeld surfaces of $\mathbf{3^A}$ plotted over d_{norm} in the range –0.2218 to 5.9790 a.u. and fingerprint plots for the Hirshfeld surface. The percentage values indicate the contribution of the interactions to the Hirshfeld surface, and the d_i and d_e values are the closest internal and external distances (Å) from given points on the Hirshfeld surface. Fingerprint plots between (a) carbon and carbon, (b) carbon and hydrogen, (c) hydrogen and carbon, (d) hydrogen and hydrogen, (e) oxygen and chlorine, (f) oxygen and hydrogen, and (g) oxygen and oxygen atoms of $\mathbf{3^A}$ and other moieties including MMF-1, respectively.

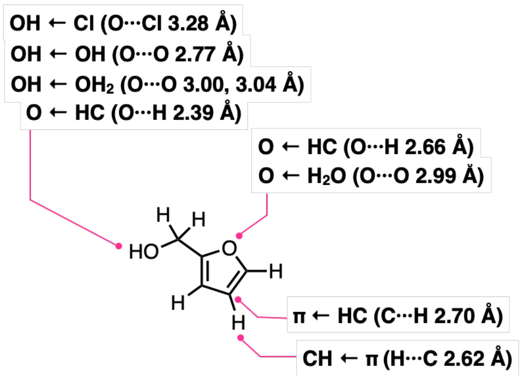

Figure S21. Summary of non-covalent interactions of **3**$^{\mathbf{A}}$ at MMF-1 in acetonitrile. Because the hydroxy group (ROH) makes a hydrogen bond with a chloride moiety, other hydrogen bonds of this moiety are attributed to lone-pair donating bonds (O:→HX).

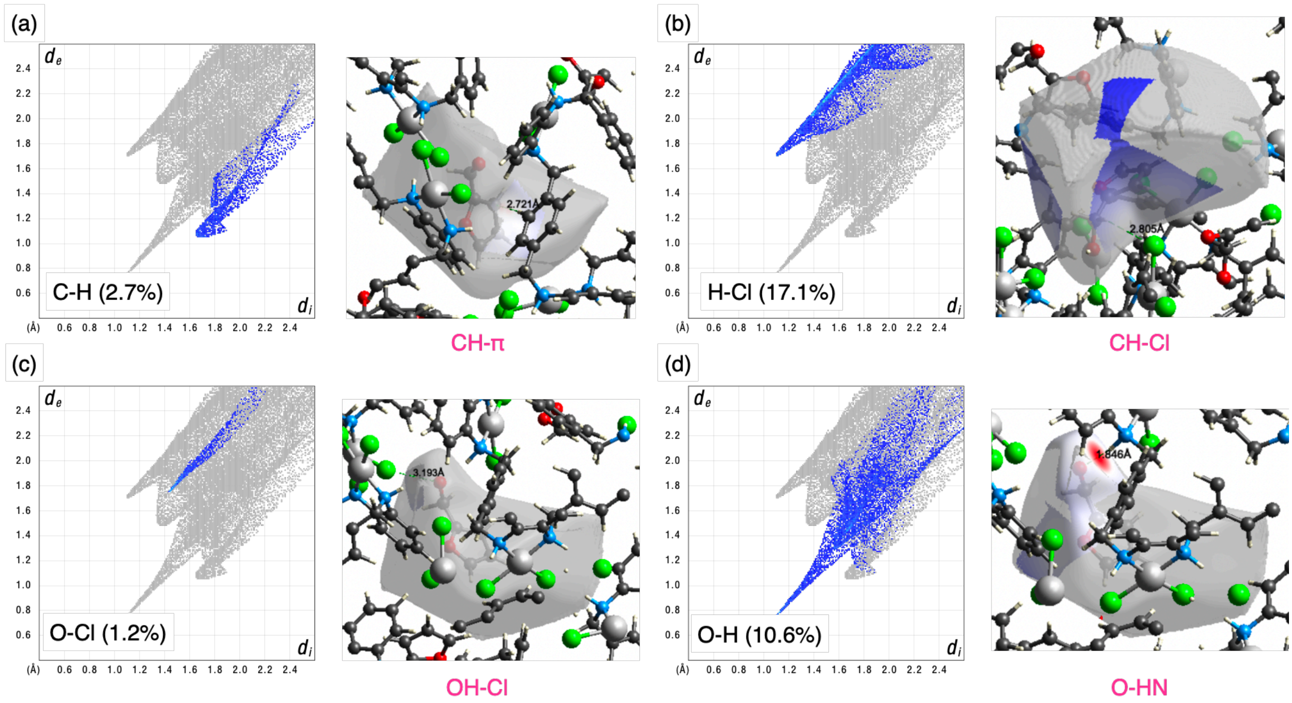

Figure S22. Three-dimensional Hirshfeld surfaces of **3**$^{\mathbf{B}}$ plotted over d_{norm} in the range –0.5883 to 6.5776 a.u. and fingerprint plots for the Hirshfeld surface. The percentage values indicate the contribution of the interactions to the Hirshfeld surface, and the d_i and d_e values are the closest internal and external distances (Å) from given points on the Hirshfeld surface. Fingerprint plots between (a) carbon and hydrogen, (b) hydrogen and chlorine, (c) oxygen and chlorine, and (d) oxygen and hydrogen atoms of **3**$^{\mathbf{B}}$ and other moieties including MMF-1, respectively.

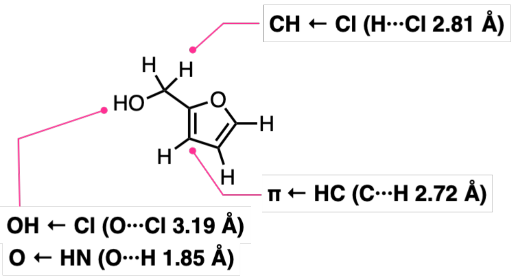

Figure S23. Summary of non-covalent interactions of $\mathbf{3^B}$ at MMF-1 in acetonitrile.

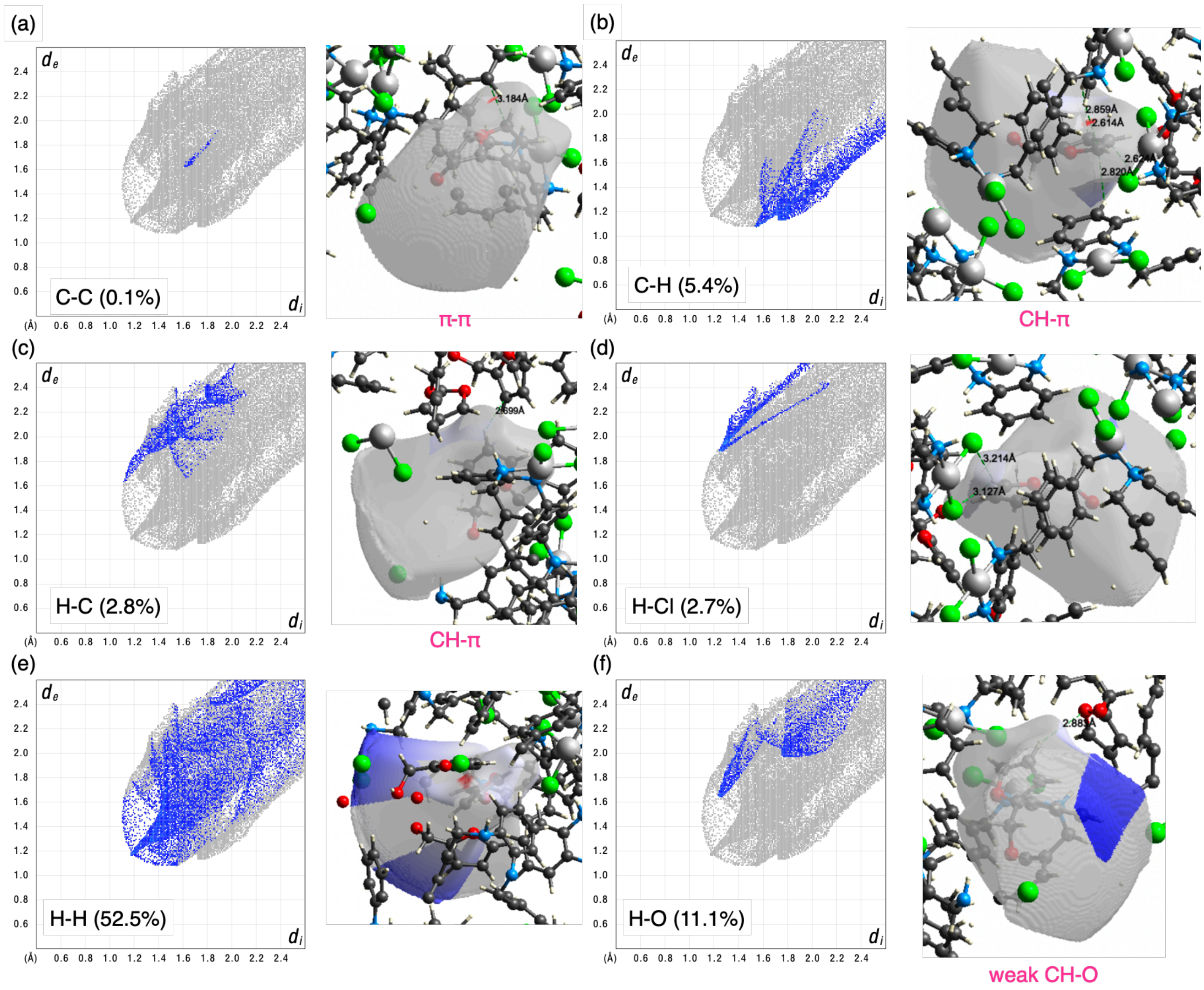

Figure S24. Three-dimensional Hirshfeld surfaces of $\mathbf{3^C}$ plotted over d_{norm} in the range –0.1262 to 7.1253 a.u. and fingerprint plots for the Hirshfeld surface. The percentage values indicate the contribution of the interactions to the Hirshfeld surface, and the d_i and d_e values are the closest internal and external distances (Å) from given points on the Hirshfeld surface. Fingerprint plots between (a) carbon and carbon, (b) carbon and hydrogen, (c) hydrogen and carbon, (d) hydrogen and chlorine, (e) hydrogen and hydrogen, and (f) hydrogen and oxygen atoms of $\mathbf{3^C}$ and other moieties including MMF-1, respectively.

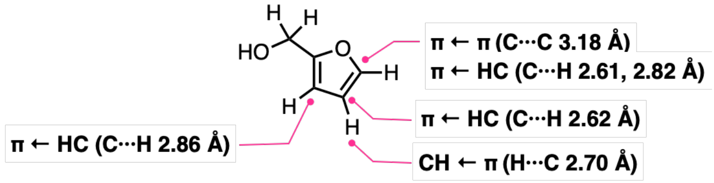

Figure S25. Summary of non-covalent interactions of $\mathbf{3^C}$ at MMF-1 in acetonitrile.

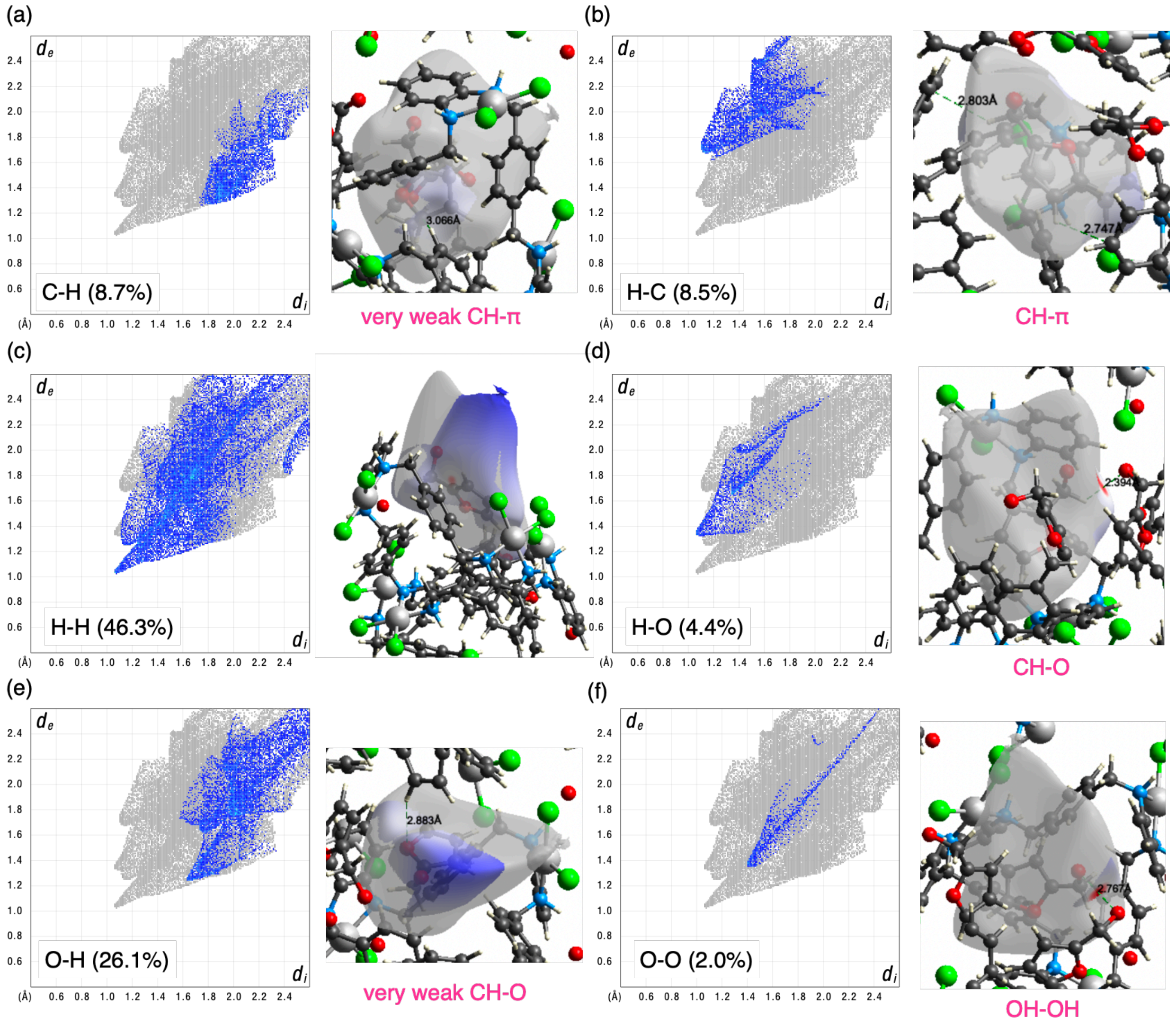

Figure S26. Three-dimensional Hirshfeld surfaces of $\mathbf{3^D}$ plotted over d_{norm} in the range –0.1783 to 5.5171 a.u. and fingerprint plots for the Hirshfeld surface. The percentage values indicate the contribution of the interactions to the Hirshfeld surface, and the d_i and d_e values are the closest internal and external distances (Å) from given points on the Hirshfeld surface. Fingerprint plots between (a) carbon and hydrogen, (b) hydrogen and carbon, (c) hydrogen and hydrogen, (d) hydrogen and oxygen, (e) oxygen and hydrogen, and (f) oxygen and oxygen atoms of $\mathbf{3^D}$ and other moieties including MMF-1, respectively. Note that disordered part of the furan ring was not taken into account for this analysis.

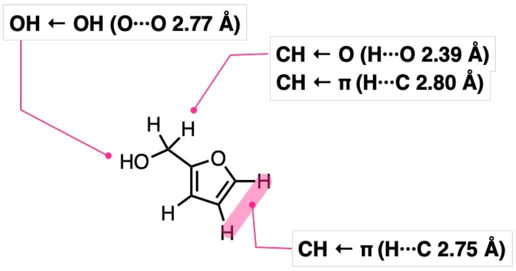

Figure S27. Summary of non-covalent interactions of 3^D at MMF-1 in acetonitrile. The ambiguous interactions caused by disorder are not included in the count.

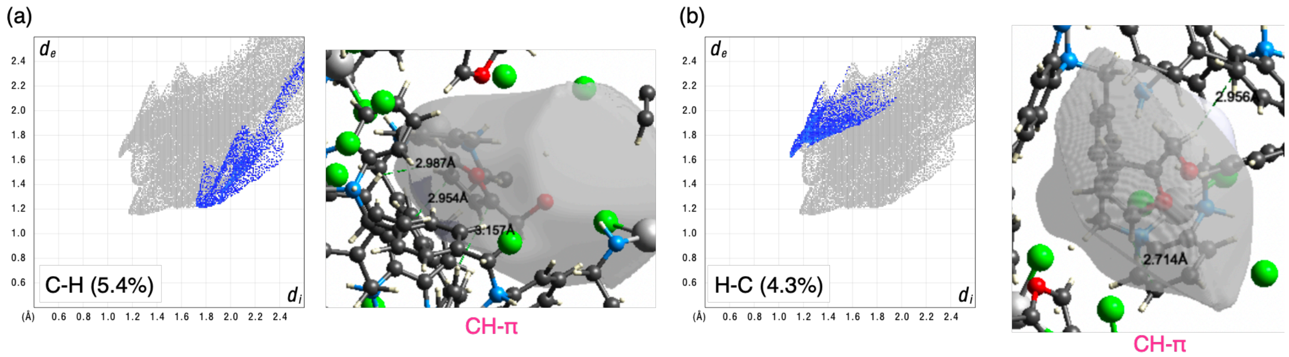

Figure S28. Three-dimensional Hirshfeld surfaces of 3^E plotted over d_{norm} in the range –0.0434 to 6.3868 a.u. and fingerprint plots for the Hirshfeld surface. The percentage values indicate the contribution of the interactions to the Hirshfeld surface, and the d_i and d_e values are the closest internal and external distances (Å) from given points on the Hirshfeld surface. Fingerprint plots between (a) carbon and hydrogen and (b) hydrogen and carbon atoms of 3^E and other moieties including MMF-1, respectively. Note that disordered part of the furan ring was not taken into account for this analysis.

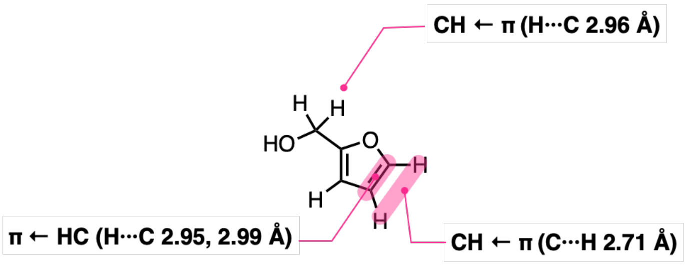

Figure S29. Summary of non-covalent interactions of 3^E at MMF-1 in acetonitrile. The ambiguous interactions caused by disorder are not included in the count.

The Hirshfeld surface analysis of guest 3^F was not conducted due to its highly disordered manner.

*5-Hydroxymethylfurfural (**4**) in acetonitrile*

MMF-1 crystals were soaked in an acetonitrile solution of **4** (1.0 M) for 19 min at room temperature. The crystals were taken out on a glass plate, mixed with Paratone oil, and then analyzed by single-crystal X-ray diffraction at –180 °C.

Crystal data for $[Pd_3LCl_6]_2\cdot(\mathbf{4})_{1.498}\cdot(CH_3CN)_{1.75}\cdot(H_2O)_{4.25}$: $C_{95.72}H_{93.71}Cl_{12}N_{13.75}O_{7.24}Pd_6$, F_w = 2616.44, crystal dimensions 0.277 × 0.195 × 0.088 mm^3, monoclinic, space group $P2_1/c$, a = 19.68940(11), b = 52.7043(4), c = 14.27080(7) Å, β = 90.9211(5)°, V = 14807.13(16) Å^3, Z = 4, ρ_{calcd} = 1.174 g cm^{-3}, μ = 8.086 cm^{-1}, T = 93 K, λ(CuKα) = 1.54184 Å, $2\theta_{max}$ = 136.498°, 163892/27086 reflections collected/unique (R_{int} = 0.0661), R_1 = 0.0859 ($I > 2\sigma(I)$), wR_2 = 0.2507 (for all data), GOF = 1.103, largest diff. peak and hole 2.787/–2.981 eÅ^{-3}. CCDC deposit number 2252162.

The occupancy of the guest molecules was refined using free variables. Their side chains were partly missing due to severe disorder. The observed side chains were assigned based on the dihedral angle of the C–C bonds. The orientation of the furan ring of the guests was determined based on the contact manner with the MMF framework. Although other very weak electron densities that may be assigned to furan rings were observed in the F_o-F_c map, they were not assigned to the guests, because their electron densities in F_o map after assignment were very unclear at the threshold of 1.0 e/Å^3. The occupancy of solvents molecules was set to be 1, 0.75 0.5, 0.33, or 0.25 based on the *Ueq* value. Hydrogen atoms of water molecules and hydroxy groups could not be located in the difference electron density maps.

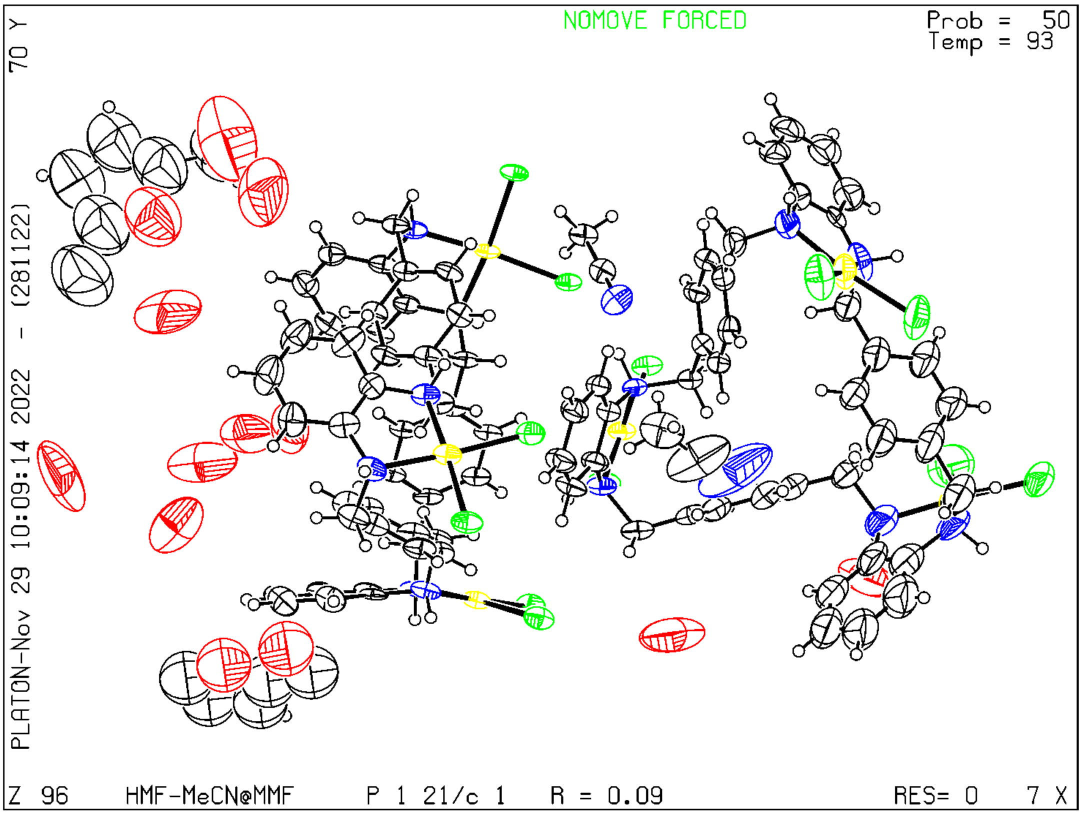

Figure S30. ORTEP drawing of the asymmetric unit of **4**@MMF-1 at the 50% probability level. Color: C grey, N blue, O red, Cl green, and Pd yellow.

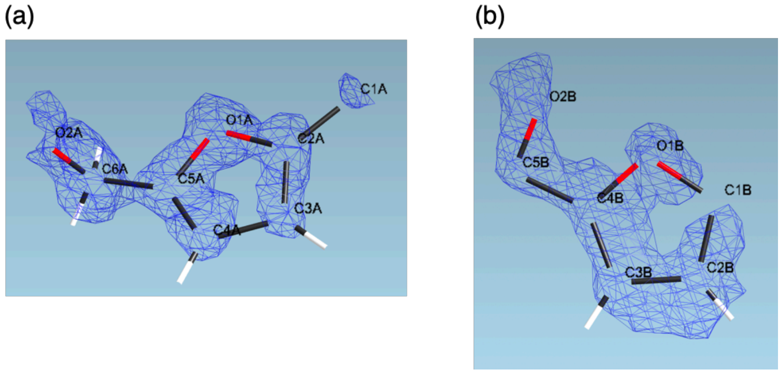

Figure S31. Electron density map of (a) $\mathbf{4}^{\mathbf{A}}$ at a macrocyclic pocket on the ceiling (contour level: 0.63σ) and (b) $\mathbf{4}^{\mathbf{B}}$ at the upper side of a bottom corner void (contour level: 0.74σ).

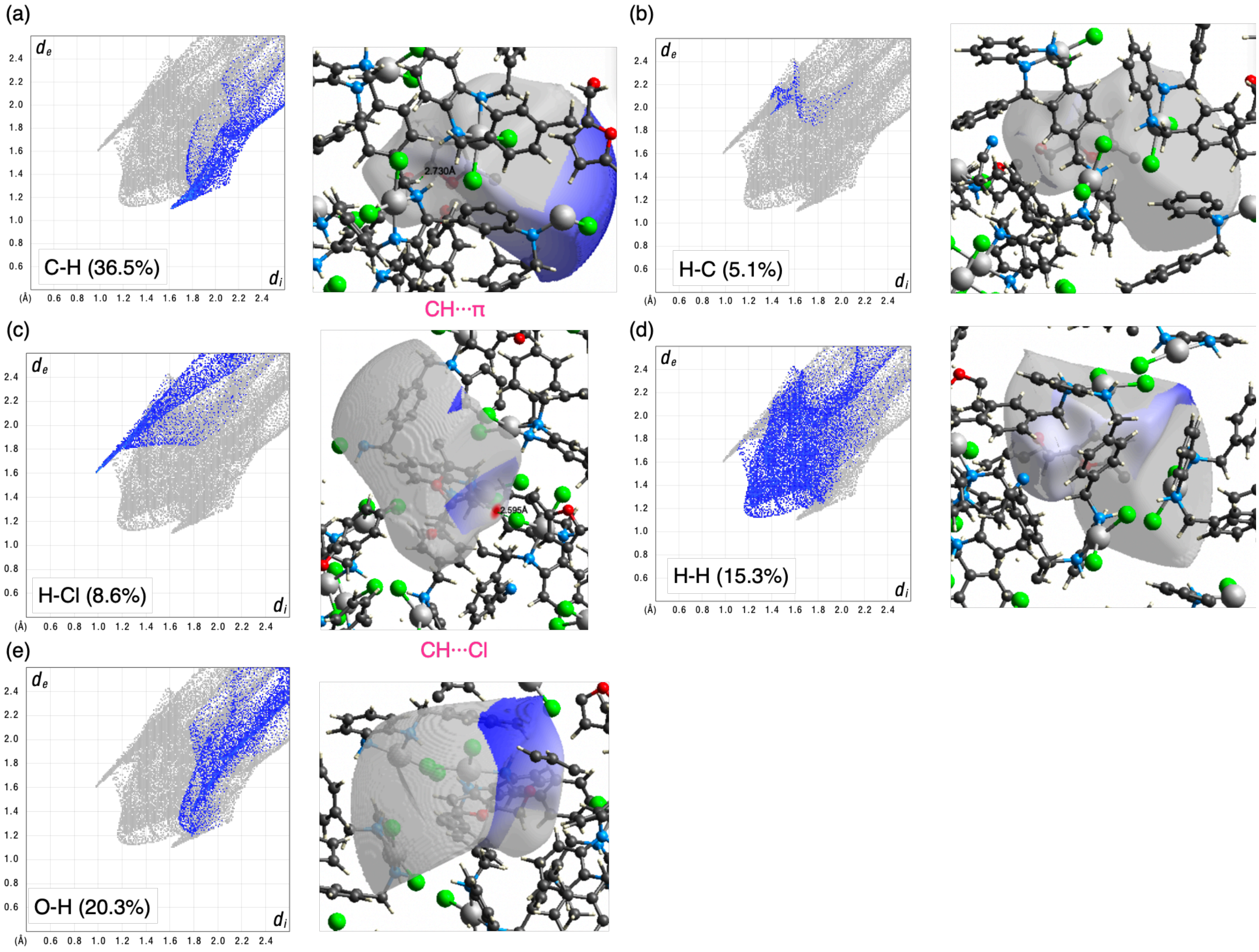

Figure S32. Three-dimensional Hirshfeld surfaces of **4^A** plotted over d_{norm} in the range –0.1733 to 6.9142 a.u. and fingerprint plots for the Hirshfeld surface. The percentage values indicate the contribution of the interactions to the Hirshfeld surface, and the d_i and d_e values are the closest internal and external distances (Å) from given points on the Hirshfeld surface. Fingerprint plots between (a) carbon and hydrogen, (b) hydrogen and carbon, (c) hydrogen and chlorine, (d) hydrogen and hydrogen, and (e) oxygen and hydrogen atoms of **4^A** and other moieties including MMF-1, respectively. Note that the oxygen atom of the formyl group could not be determined.

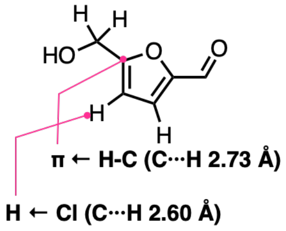

Figure S33. Summary of non-covalent interactions of **4^A** at MMF-1 in acetonitrile.

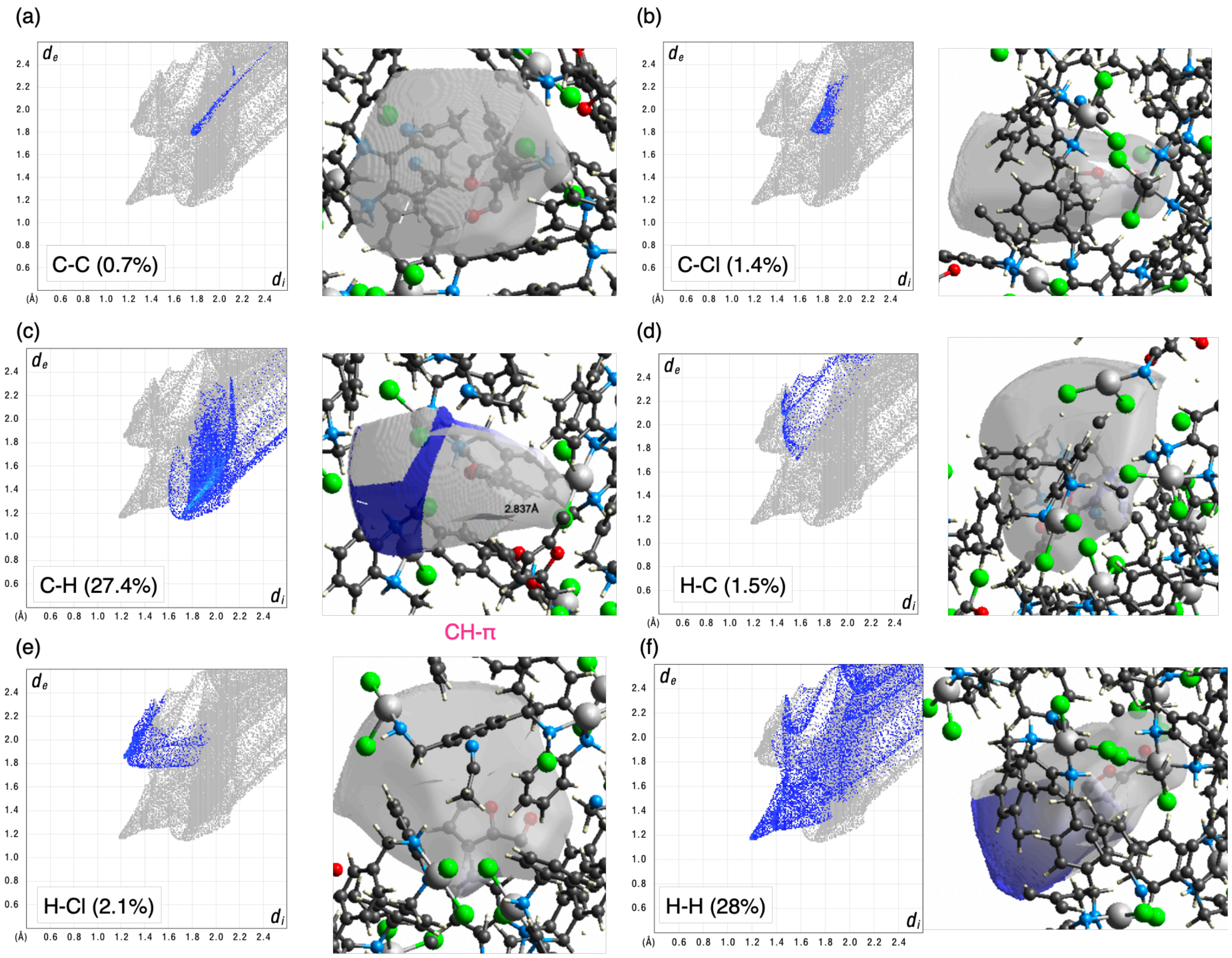

Figure S34. Three-dimensional Hirshfeld surfaces of **4**$^{\mathbf{B}}$ plotted over d_{norm} in the range –0.0775 to 6.6646 a.u. and fingerprint plots for the Hirshfeld surface. The percentage values indicate the contribution of the interactions to the Hirshfeld surface, and the d_i and d_e values are the closest internal and external distances (Å) from given points on the Hirshfeld surface. Fingerprint plots between (a) carbon and carbon, (b) carbon and chlorine, (c) carbon and hydrogen, (d) hydrogen and carbon, (e) hydrogen and chlorine, and (f) hydrogen and hydrogen atoms of **4**$^{\mathbf{B}}$ and other moieties including MMF-1, respectively. Note that the type of the functionality of one side chain and the position of the other side chain could not be determined.

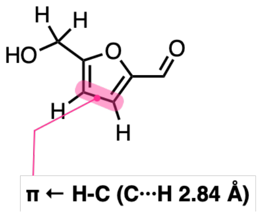

Figure S35. Summary of non-covalent interactions of **4**$^{\mathbf{B}}$ at MMF-1 in acetonitrile. The ambiguous interactions caused by disorder are not included in the count.

*Furan (**5**) in acetonitrile*

MMF-1 crystals were soaked in an acetonitrile solution of furan (**5**) (1.0 M) for 22 min at room temperature. The crystals were taken out on a glass plate, mixed with Paratone oil, and then analyzed by single-crystal X-ray diffraction at –180 °C.

Crystal data for $[Pd_3LCl_6]_2\cdot(CH_3CN)_{4.25}\cdot(H_2O)_{8.133}$: $C_{92.50}H_{96.75}Cl_{12}N_{16.25}O_{8.13}Pd_6$, $F_w = 2630.03$, crystal dimensions 0.38 × 0.229 × 0.086 mm^3, monoclinic, space group $P2_1/c$, $a = 19.57180(14)$, $b = 51.7499(5)$, $c = 14.25090(10)$ Å, $\beta = 90.8846(6)°$, $V = 14432.1(2)$ Å^3, $Z = 4$, $\rho_{calcd} = 1.210$ g cm^{-3}, $\mu = 8.308$ cm^{-1}, $T = 93$ K, λ(CuKα) = 1.54184 Å, $2\theta_{max} = 136.498°$, 154719/26292 reflections collected/unique ($R_{int} = 0.1064$), $R_1 = 0.1522$ ($I > 2\sigma(I)$), $wR_2 = 0.3792$ (for all data), GOF = 1.089, largest diff. peak and hole 5.269/–2.485 eÅ^{-3}. CCDC deposit number 2252165.

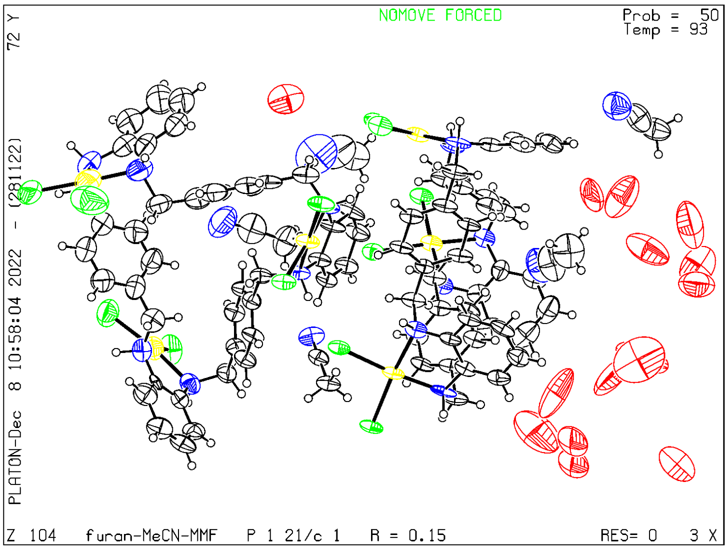

Figure S36. ORTEP drawing of the asymmetric unit at the 50% probability level. Color: C grey, N blue, O red, Cl green, and Pd yellow.

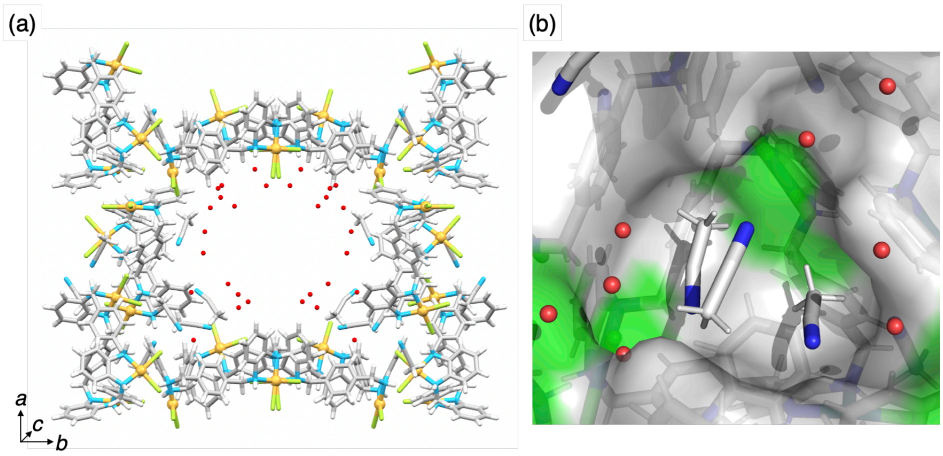

Figure S37. Crystal structure soaked in an acetonitrile solution of **5**. (a) MMF-1 nanochannel. (b) (*M*)-corner void with the MMF-1 surface and solvents represented as the stick model.

5-Hydroxymethylfurfural (4) in water

MMF-1 crystals were soaked in an aqueous solution of **4** (1.0 M) for 1 h at room temperature. The crystals were taken out on a glass plate, mixed with Paratone oil, and then analyzed by single-crystal X-ray diffraction at –180 °C.

Crystal data for $[Pd_3LCl_6]_2\cdot(\mathbf{4})_{1.0}\cdot(H_2O)_{4.25}$: $C_{89.71}H_{88.14}Cl_{12}N_{12}O_{10.22}Pd_6$, F_w = 2561.68, crystal dimensions 0.196 × 0.138 × 0.059 mm^3, monoclinic, space group $P2_1/c$, a = 19.65460(19), b = 52.6684(5), c = 14.16700(11) Å, β = 91.4550(8)°, V = 14660.6(2) Å^3, Z = 4, ρ_{calcd} = 1.161 g cm^{-3}, μ = 8.168 cm^{-1}, T = 93 K, λ(CuKα) = 1.54184 Å, $2\theta_{max}$ = 136.50°, 76665/26750 reflections collected/unique (R_{int} = 0.0408), R_1 = 0.0764 ($I > 2\sigma(I)$), wR_2 = 0.2380 (for all data), GOF = 1.049, largest diff. peak and hole 2.922/–2.520 eÅ^{-3}. CCDC deposit number 2252163.

The occupancy of the guest molecules was refined using free variables. The structure of one of the guests was partially assigned due to severe disorder, and the type of its side chain (hydroxymethyl or aldehyde) could not be determined due to its ambiguous electron density, so the hydrogen atoms of its carbon atom were not assigned. The orientation of the furan ring of the weaker guest was determined based on the contact manner with the MMF framework. Although other very weak electron densities that may be assigned to furan rings were observed in the F_o-F_c map, they were not assigned to the guests, because their electron densities in F_o map after assignment were very unclear at the threshold of 1.0 e/Å^3. The occupancy of water molecules disordered with the guests were refined using free variables, and that of other waters were set to be 1, 0.5, 0.33, or 0.25 based on the *Ueq* value. Hydrogen atoms of water molecules and hydroxy group could not be located in the difference electron density maps.

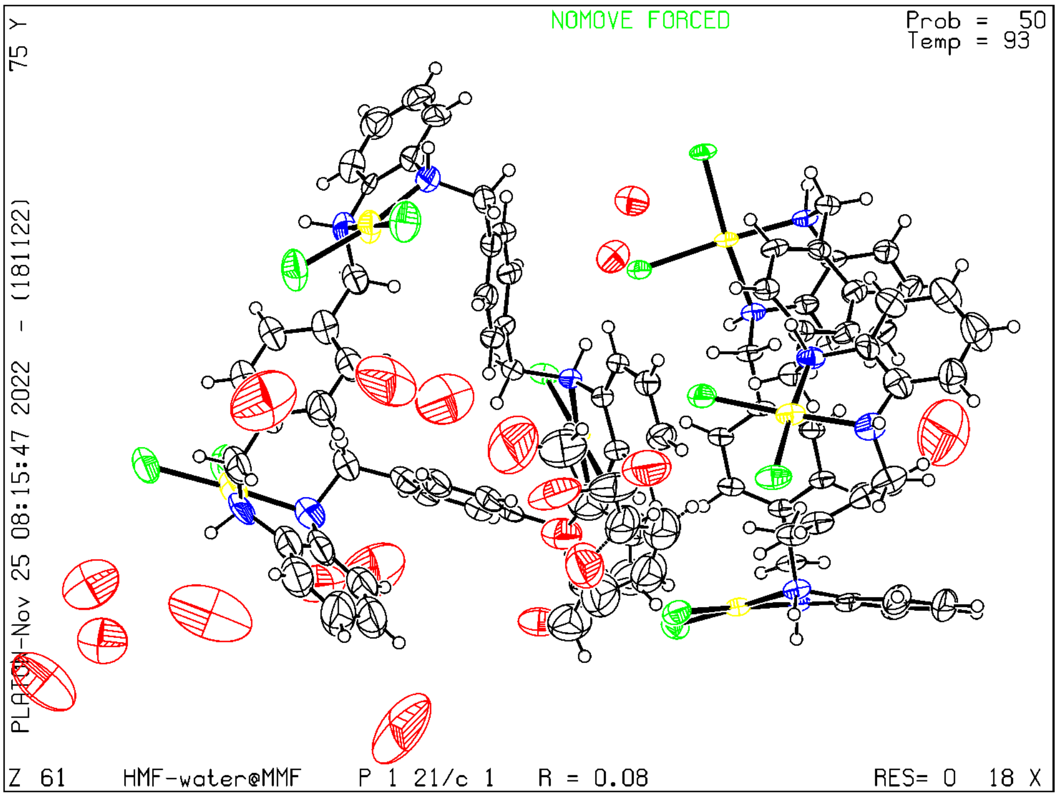

Figure S38. ORTEP drawing of the asymmetric unit of **4**@MMF-1 at the 50% probability level. Color: C grey, N blue, O red, Cl green, and Pd yellow.

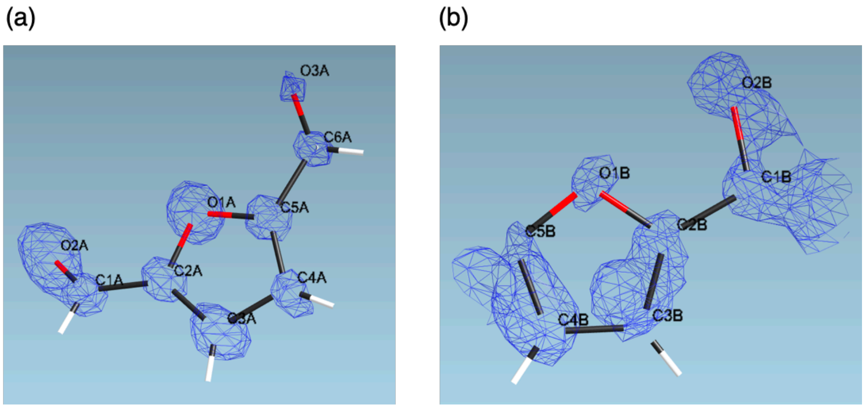

Figure S39. Electron density map of (a) $\mathbf{4^A}$ at the lower side of a bottom corner void (contour level: 1.0σ) and (b) $\mathbf{4^B}$ at the upper side of a bottom corner void (contour level: 0.6σ).

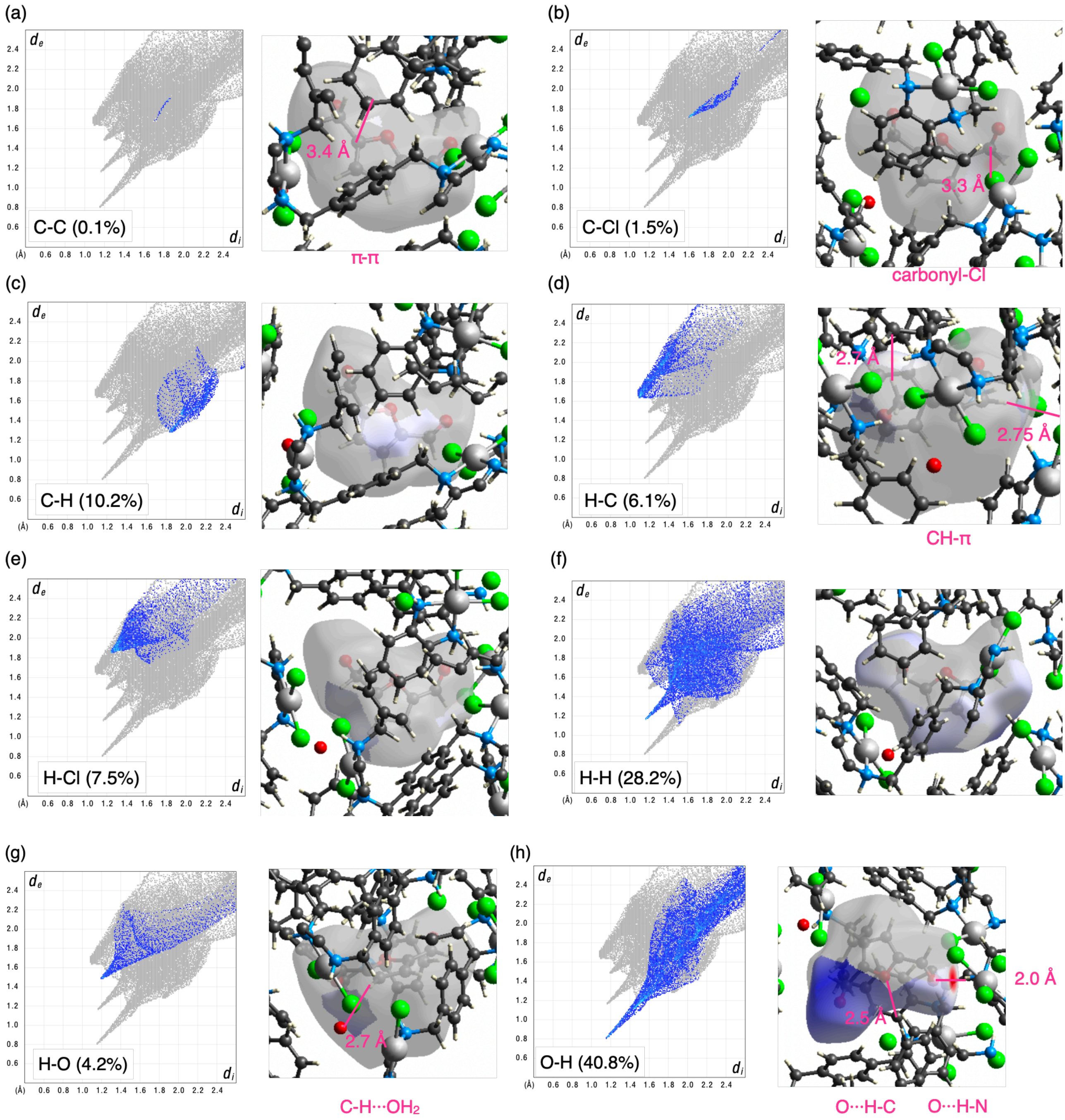

Figure S40. Three-dimensional Hirshfeld surfaces of $\mathbf{4}^{\mathbf{A}}$ plotted over d_{norm} in the range –0.4970 to 4.8828 a.u. and fingerprint plots for the Hirshfeld surface. The percentage values indicate the contribution of the interactions to the Hirshfeld surface, and the d_i and d_e values are the closest internal and external distances (Å) from given points on the Hirshfeld surface. Fingerprint plots between (a) carbon and carbon, (b) carbon and chlorine, (c) carbon and hydrogen, (d) hydrogen and carbon, (e) hydrogen and chlorine, (f) hydrogen and hydrogen, (g) hydrogen and oxygen, and (h) oxygen and hydrogen atoms of $\mathbf{4}^{\mathbf{A}}$ and other moieties including MMF-1, respectively.

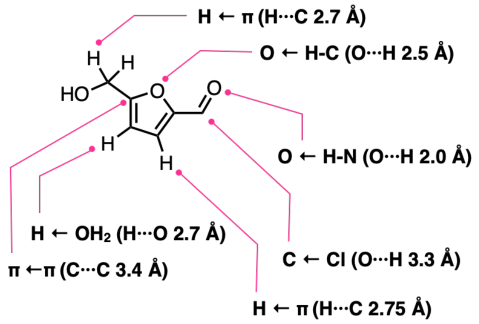

Figure S41. Summary of non-covalent interactions of $\mathbf{4^A}$ at MMF-1 in water.

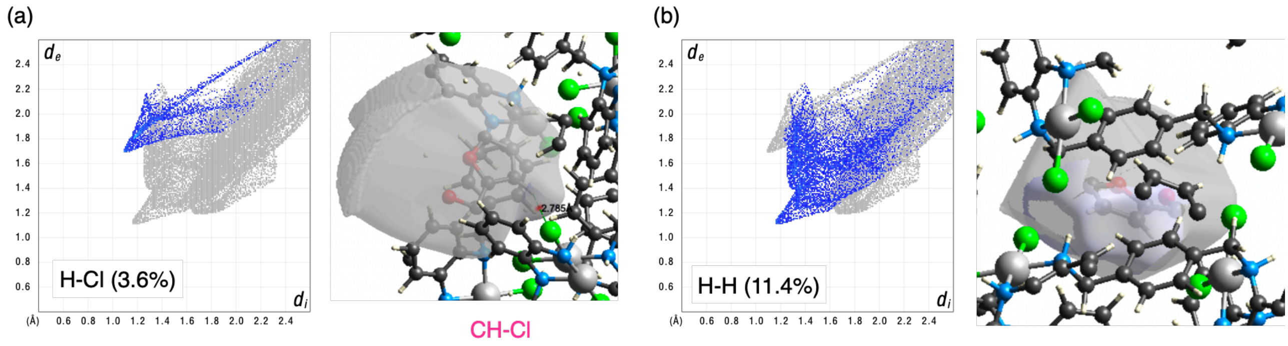

Figure S42. Three-dimensional Hirshfeld surfaces of $\mathbf{4^B}$ plotted over d_{norm} in the range –0.0331 to 5.9292 a.u. and fingerprint plots for the Hirshfeld surface. The percentage values indicate the contribution of the interactions to the Hirshfeld surface, and the d_i and d_e values are the closest internal and external distances (Å) from given points on the Hirshfeld surface. Fingerprint plots between (a) hydrogen and chlorine and (b) hydrogen and hydrogen atoms of $\mathbf{4^B}$ and other moieties including MMF-1. Note that the type of the side chains could not be determined.

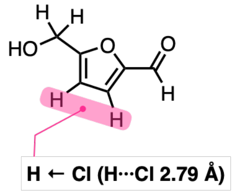

Figure S43. Summary of non-covalent interactions of $\mathbf{4^B}$ at MMF-1 in water. The ambiguous interactions caused by disorder are not included in the count.

5-Hydroxymethylfurfural (4) in chloroform

MMF-1 crystals were soaked in a chloroform solution of **4** (1.0 M) for 35 min at room temperature. The crystals were taken out on a glass plate, mixed with Paratone oil, and then analyzed by single-crystal X-ray diffraction at –180 °C.

Crystal data for $[Pd_3LCl_6]_2\cdot(\mathbf{4})_{0.616}\cdot(CHCl_3)_{0.583}\cdot(CH_3CN)_{0.672}\cdot(H_2O)_{4.791}$: $C_{89.56}H_{87.79}Cl_{13.75}N_{12.66}O_{6.64}Pd_6$, F_w = 2573.63, crystal dimensions 0.336 × 0.255 × 0.099 mm^3, monoclinic, space group $P2_1/c$, a = 19.6237(9), b = 52.6924(15), c = 14.2250(3) Å, β = 91.251(3)°, V = 14705.4(9) Å^3, Z = 4, ρ_{calcd} = 1.162 g cm^{-3}, μ = 8.414 cm^{-1}, T = 93 K, λ(CuKα) = 1.54184 Å, $2\theta_{max}$ = 136.498°, 110788/26752 reflections collected/unique (R_{int} = 0.1881), R_1 = 0.1694 ($I > 2\sigma(I)$), wR_2 = 0.4651 (for all data), GOF = 1.529, largest diff. peak and hole 3.218/–2.269 eÅ^{-3}. CCDC deposit number 2252164.

The occupancies of the guest molecules were refined using free variables. Since the structural analysis could not determine which of the side chains was the hydroxymethyl group, the hydrogen atoms on the side chains were not located. The occupancies of solvent molecules were set to be 1, 0.75 0.5, 0.33, or 0.25 based on the *Ueq* value except for disordered solvents whose occupancies were refined using free variables. Hydrogen atoms of water molecules could not be located in the difference electron density maps.

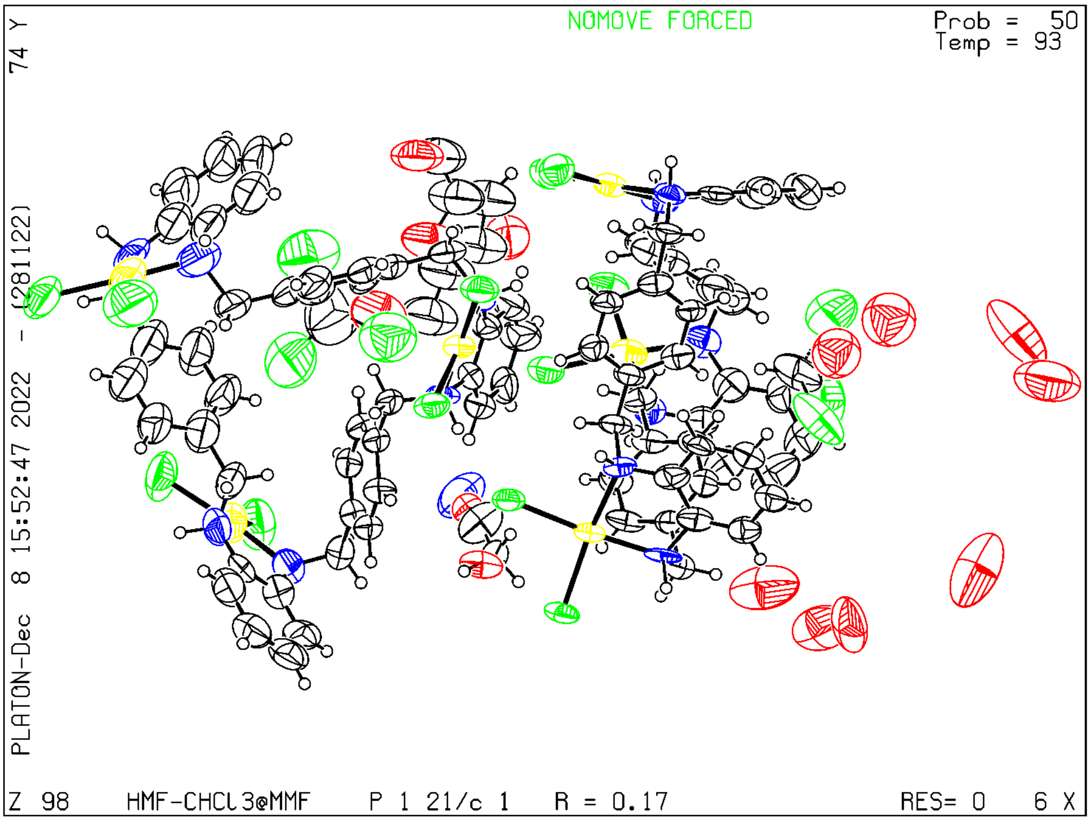

Figure S44. ORTEP drawing of the asymmetric unit of **4**@MMF-1 at the 50% probability level. Color: C grey, N blue, O red, Cl green, and Pd yellow.

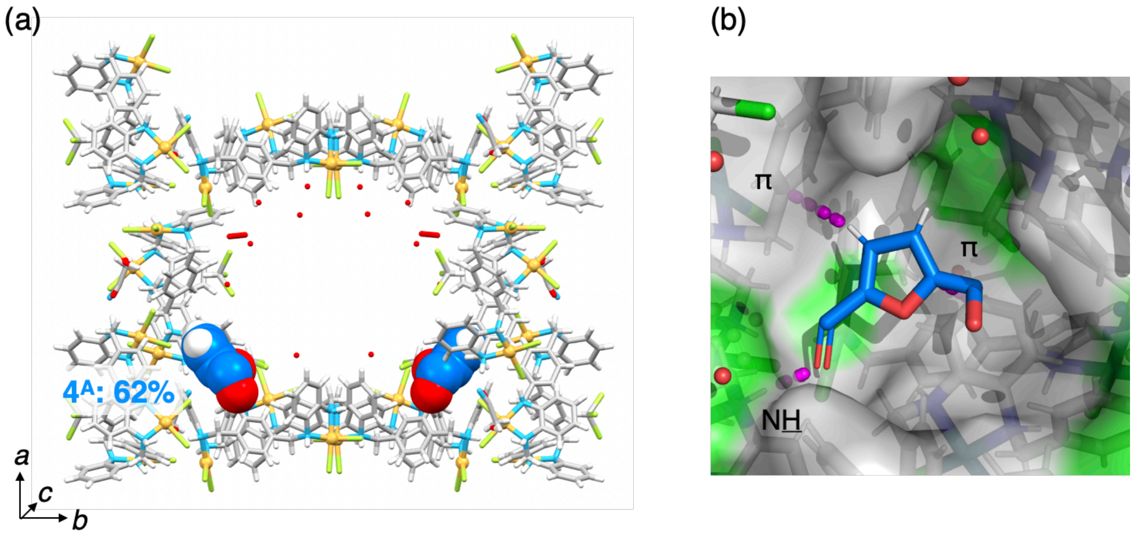

Figure S45. Crystal structure soaked in a chloroform solution of **4**. (a) MMF-1 nanochannel. (b) (*M*)-corner void with the MMF-1 surface and **4** represented as the stick model.

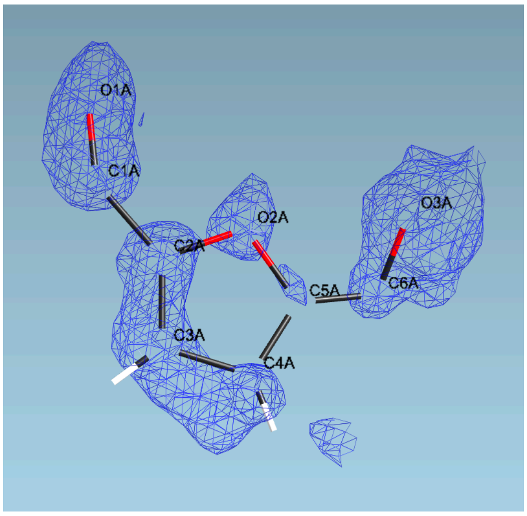

Figure S46. Electron density map of **4** at the lower side of a bottom corner void (contour level: 0.69σ).

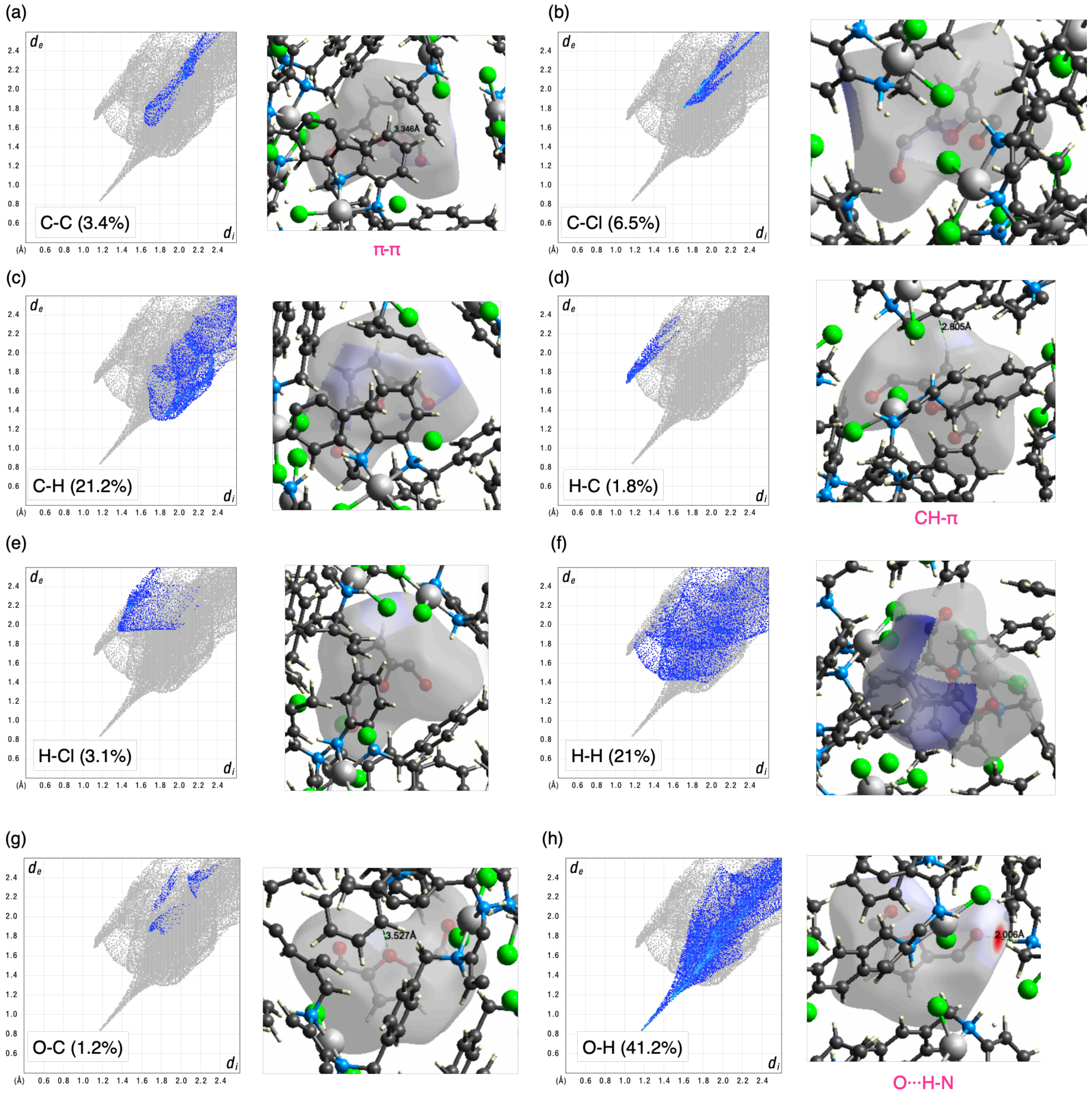

Figure S47. Three-dimensional Hirshfeld surfaces of **4** plotted over d_{norm} in the range –0.4619 to 4.8997 a.u. and fingerprint plots for the Hirshfeld surface. The percentage values indicate the contribution of the interactions to the Hirshfeld surface, and the d_i and d_e values are the closest internal and external distances (Å) from given points on the Hirshfeld surface. Fingerprint plots between (a) carbon and carbon, (b) carbon and chlorine, (c) carbon and hydrogen, (d) hydrogen and carbon, (e) hydrogen and chlorine, (f) hydrogen and hydrogen, (g) oxygen and carbon, and (h) oxygen and hydrogen atoms of **4** and other moieties including MMF-1, respectively. Note that the type of the side chains could not be determined, and the classical hydrogen bond of hydroxy or formyl group was not included in the analysis.

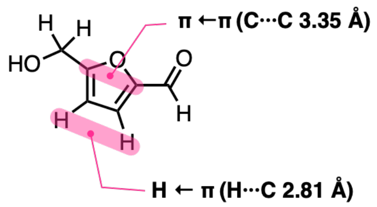

Figure S48. Summary of non-covalent interactions of **4** at MMF-1 in chloroform. The ambiguous interactions caused by disorder are not included in the count.

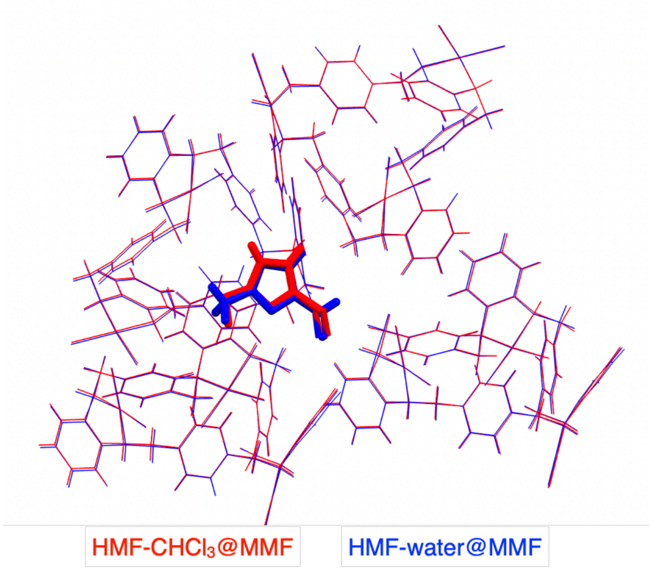

Figure S49. Overlayed binding structure of **4** at a bottom corner void in water (blue) and in chloroform (red).

Detailed binding structures at the ceiling sites

Since detailed binding structures at the corner voids are compared in the main text (Figure 2 and 3), the remaining binding structures at the ceiling sites are summarized here to clarify the differences in adsorption positions and configurations of all the guest molecules.

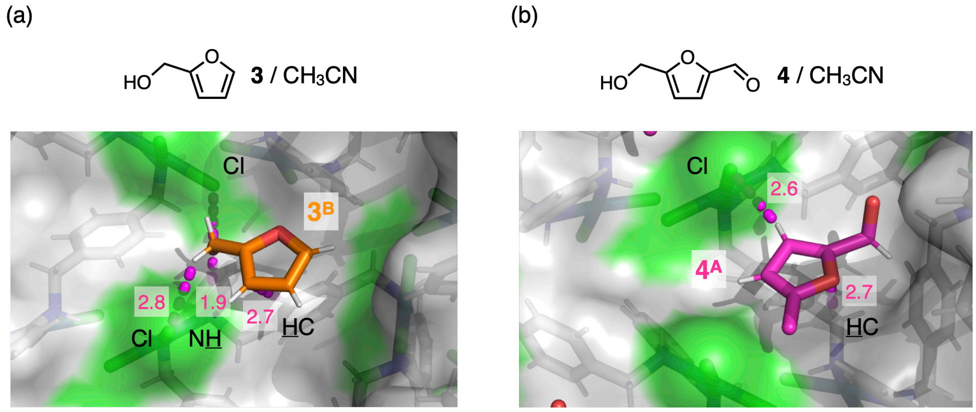

Figure S50. Binding structures of (a) **3** and (b) **4** at the ceiling sites of MMF-1 soaked in their acetonitrile solution. Representation of the molecules, colors, and notations are identical to those of Figure 2 in this manuscript.

Full Interaction Maps analysis of furanic compounds

The atomic coordinates of the furanic compounds were generated by DFT calculation at the B3LYP/6-31G* level in gas state, which was conducted with the Spartan 16 program.[8] The Full Interaction Maps analysis[9] was carried out on the Mercury[4] program.

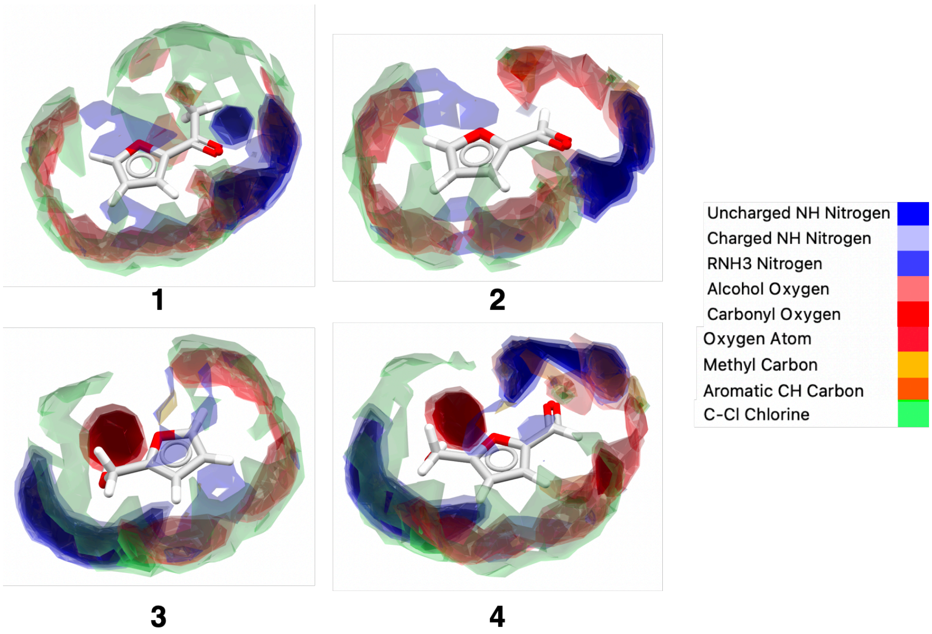

Figure S51. Full Interaction Maps of **1**, **2**, **3**, and **4** based on the Cambridge Structural Database.

Separation of HMF from decomposed cellulose using MMF-1

Decomposition of cellulose by microwave heating

Microcrystalline cellulose (117 mg) dispersed in deionized water (5.0 mL) was heated by microwave irradiation at 210 °C for 100 min. The resultant slurry (Figure S52a) was removed by filtration, and the filtrate was concentrated to about eight times its original concentration. A portion of the concentrate was dried and analyzed by ^{1}H NMR spectroscopy (Figure S52b).

Extraction from the mixture of decomposed cellulose using MMF-1

MMF-1 crystals were soaked into the concentrate at 30 °C for 3 h, collected, and then resoaked into pure D_2O for 1 h to extract compounds adsorbed to MMF-1. The extract was analyzed by ^{1}H NMR spectroscopy (Figure S52c).

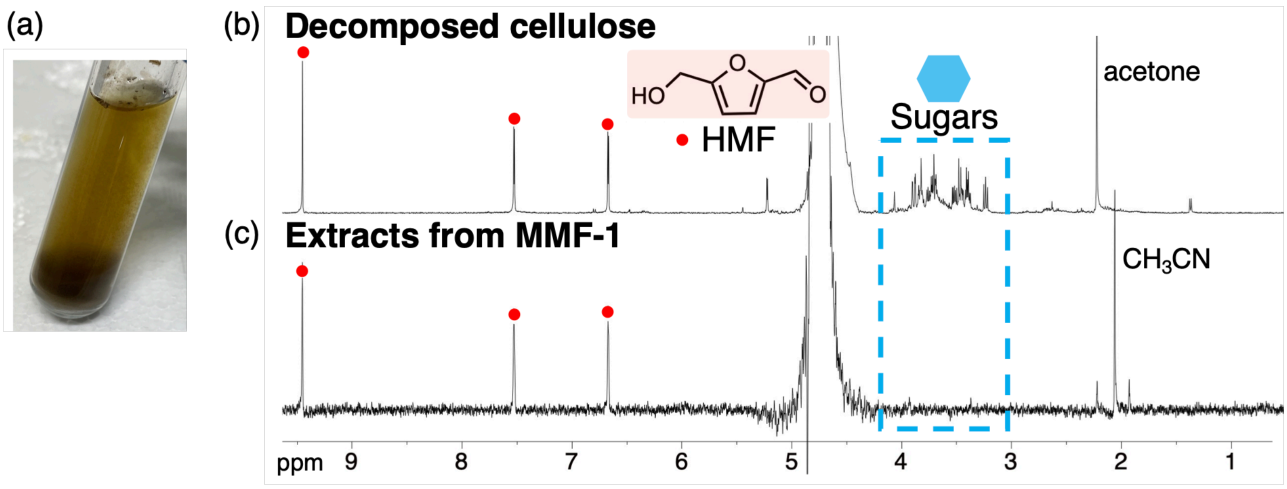

Figure S52. (a) Photograph of decomposed cellulose with slurry. (b),(c) ^{1}H NMR spectra (500 MHz, D_2O, 300 K) of decomposed cellulose (b) and the extract from MMF-1 (c).

References

[1] a) S. Tashiro, R. Kubota, M. Shionoya, *J. Am. Chem. Soc.* **2012**, *134*, 2461–2464; b) S. Tashiro, M. Shionoya, *Bull. Chem. Soc. Jpn.* **2014**, *87*, 643–654; c) S. Tashiro, M. Shionoya, *Acc. Chem. Res.* **2020**, *53*, 632–643; d) R. Hayashi, S. Tashiro, M. Asakura, S. Mitsui, M. Shionoya, *Nat. Commun.* **2023**, *14*, 4490.

[2] O. V. Dolomanov, L. J. Bourhis, R. J. Gildea, J. A. K. Howard, H. Puschmann, *J. Appl. Cryst.* **2009**, *42*, 339–341.

[3] G. M. Sheldrick, *Acta Cryst*. **2015**, *C71*, 3–8.

[4] C. F. Macrae, I. Sovago, S. J. Cottrell, P. T. A. Galek, P. MaCabe, E. Pidcock, M. Platings, G. P. Shields, J. S. Stevens, M. Towler, P. A. Wood, *J. Appl. Cryst.* **2020**, *53*, 226–235.

[5] The PyMOL Molecular Graphics System, Version 2.5.2 Schrödinger, LLC.

[6] C. B. Hübschle, G. M. Sheldrick, B. Dittrich, *J. Appl. Cryst.* **2011**, *44*, 1281–1284.

[7] M. A. Spackman, D. Jayatilaka, *CrystEngComm* **2009**, *11*, 19–32.

[8] Spartan'16 Wavefunction, Inc., Irvine, CA.

[9] P. A. Wood, T. S. G. Olsson, J. C. Cole, S. J. Cottrell, N. Feeder, P. T. A. Galek, C. R. Groom, E. Pidcock, *CrystEngComm* **2013**, *15*, 65–72.
