# Supplementary material for: Porous Supramolecular Crystalline Probe that Detects Non‐Covalent Interactions Involved in Molecular Recognition of Furanic Compounds
Source: Small. 2024 Jul 30;20(49):2405507. doi: 10.1002/smll.202405507 (PMC11618713; doi:10.1002/smll.202405507)

checkCIF/PLATON report

Structure factors have been supplied for datablock(s) HMF-CHCl3@MMF

THIS REPORT IS FOR GUIDANCE ONLY. IF USED AS PART OF A REVIEW PROCEDURE FOR PUBLICATION, IT SHOULD NOT REPLACE THE EXPERTISE OF AN EXPERIENCED CRYSTALLOGRAPHIC REFEREE.

No syntax errors found. CIF dictionary Interpreting this report

Datablock: HMF-CHCl3@MMF

Bond precision:	C-C = 0.0172 A	Wavelength=1.54184	
Cell:	a=19.6237(9)	b=52.6924(15)	c=14.2250(3)
	alpha=90	beta=91.251(3)	gamma=90
Temperature:	93 K		

	Calculated	Reported
Volume	14705.4(9)	14705.4(9)
Space group	P 21/c	P 1 21/c 1
Hall group	-P 2ybc	-P 2ybc
Moiety formula	C42 H42 Cl6 N6 Pd3, 0.31(C6 H2 O3), 0.292(C H Cl3), 0.335(C2 H3	2(C42 H42 Cl6 N6 Pd3), 4.791(O), 0.616(C6 H2 O3), 0.583(C H Cl3
Sum formula	C44.82 H43.92 Cl6.87 N6.34 O3.33 Pd3	C89.56 H87.79 Cl13.75 N12.66 O6.64 Pd6
Mr	1287.49	2573.63
Dx,g cm-3	1.163	1.162
Z	8	4
Mu (mm-1)	8.414	8.414
F000	5109.5	5107.0
F000'	5143.09	
h,k,lmax	23,63,17	23,63,17
Nref	26910	26752
Tmin,Tmax	0.114,0.435	0.363,1.000
Tmin'	0.039	

Correction method= # Reported T Limits: Tmin=0.363 Tmax=1.000
AbsCorr = MULTI-SCAN

Data completeness= 0.994 Theta(max)= 68.249

R(reflections)= 0.1694(16053) wR2(reflections)= 0.4651(26752)

S = 1.529 Npar= 1165

The following ALERTS were generated. Each ALERT has the format
test-name_ALERT_alert-type_alert-level.
Click on the hyperlinks for more details of the test.

Alert level A

PLAT084_ALERT_3_A High wR2 Value (i.e. > 0.25) .... 0.47 Report

Author Response: Some solvents and guests in the large pore could not be located due to severe disordering. Therefore, the data quality was not high enough.

PLAT602_ALERT_2_A Solvent Accessible VOID(S) in Structure .... ! Check

Author Response: Some solvents in the large pore could not be located due to severe disordering.

PLAT973_ALERT_2_A Check Calcd Positive Resid. Density on Pd1 2.99 eA-3

Author Response: The atom type is correct and there is no evidence of twinning. The large residual density on Pd atoms may be Due to an anomalous dispersion effect and has no chemical significance.

Alert level B

RINTA01_ALERT_3_B The value of Rint is greater than 0.18
Rint given 0.188

Author Response: Some solvents and guests in the large pore could not be located due to severe disordering. Therefore, the data quality was not high enough.

PLAT020_ALERT_3_B The Value of Rint is Greater Than 0.12 .... 0.188 Report

Author Response: Some solvents and guests in the large pore could not be located due to severe disordering. Therefore, the data quality was not high enough.

PLAT082_ALERT_2_B High R1 Value .... 0.17 Report

Author Response: Some solvents and guests in the large pore could not be located due to severe disordering. Therefore, the data quality was not high enough.

PLAT306_ALERT_2_B Isolated Oxygen Atom (H-atoms Missing ?) .... O8W Check

Author Response: Hydrogen atoms of water molecules could not be located in the difference electron density maps.

PLAT971_ALERT_2_B Check Calcd Resid. Dens. 1.04Ang From Pd1 3.33 eA-3

Author Response: The atom type is correct and there is no evidence of twinning.

PLAT971_ALERT_2_B Check Calcd Resid. Dens. 1.06Ang From Pd1 3.10 eA-3

Author Response: The atom type is correct and there is no evidence of twinning.

PLAT971_ALERT_2_B Check Calcd Resid. Dens. 0.62Ang From Pd1 3.10 eA-3

Author Response: The atom type is correct and there is no evidence of twinning.

PLAT971_ALERT_2_B Check Calcd Resid. Dens. 0.61Ang From Pd1 3.08 eA-3

Author Response: The atom type is correct and there is no evidence of twinning.

PLAT971_ALERT_2_B Check Calcd Resid. Dens. 0.91Ang From Pd1 2.88 eA-3

Author Response: The atom type is correct and there is no evidence of twinning.

PLAT971_ALERT_2_B Check Calcd Resid. Dens. 1.02Ang From N1 2.84 eA-3

Author Response: The atom type is correct and there is no evidence of twinning.

PLAT971_ALERT_2_B Check Calcd Resid. Dens. 1.13Ang From Pd4 2.77 eA-3

Author Response: The atom type is correct and there is no evidence of twinning.

PLAT971_ALERT_2_B Check Calcd Resid. Dens. 0.72Ang From Pd2 2.71 eA-3

Author Response: The atom type is correct and there is no evidence of twinning.

```
PLAT971_ALERT_2_B Check Calcd Resid. Dens.  0.98Ang From Pd4            2.61 eA-3
```

Author Response: The atom type is correct and there is no evidence of twinning.

```
PLAT971_ALERT_2_B Check Calcd Resid. Dens.  0.95Ang From Pd1            2.58 eA-3
```

Author Response: The atom type is correct and there is no evidence of twinning.

Alert level C

```
PLAT041_ALERT_1_C Calc. and Reported SumFormula    Strings  Differ     Please Check
PLAT068_ALERT_1_C Reported F000 Differs from Calcd (or Missing)...     Please Check
PLAT077_ALERT_4_C Unitcell Contains Non-integer Number of Atoms ..     Please Check
PLAT213_ALERT_2_C Atom N1              has ADP max/min Ratio .....        3.3 oblate
PLAT213_ALERT_2_C Atom C15             has ADP max/min Ratio .....        3.6 oblate
PLAT213_ALERT_2_C Atom N11             has ADP max/min Ratio .....        3.3 prolat
PLAT220_ALERT_2_C NonSolvent   Resd 2  C   Ueq(max)/Ueq(min) Range        3.3 Ratio
PLAT220_ALERT_2_C NonSolvent   Resd 2 Cl   Ueq(max)/Ueq(min) Range        3.1 Ratio
PLAT230_ALERT_2_C Hirshfeld Test Diff for    C15      --C20      .        5.5 s.u.
PLAT230_ALERT_2_C Hirshfeld Test Diff for    N7       --C43      .        5.3 s.u.
PLAT234_ALERT_4_C Large Hirshfeld Difference Pd1      --N1       .       0.18 Ang.
PLAT234_ALERT_4_C Large Hirshfeld Difference Pd2      --N3       .       0.16 Ang.
PLAT234_ALERT_4_C Large Hirshfeld Difference N1       --C42      .       0.18 Ang.
PLAT234_ALERT_4_C Large Hirshfeld Difference N3       --C15      .       0.21 Ang.
PLAT234_ALERT_4_C Large Hirshfeld Difference N4       --C20      .       0.19 Ang.
PLAT234_ALERT_4_C Large Hirshfeld Difference N5       --C29      .       0.19 Ang.
PLAT234_ALERT_4_C Large Hirshfeld Difference C5       --C6       .       0.23 Ang.
PLAT234_ALERT_4_C Large Hirshfeld Difference C9       --C10      .       0.18 Ang.
PLAT234_ALERT_4_C Large Hirshfeld Difference C16      --C17      .       0.23 Ang.
PLAT234_ALERT_4_C Large Hirshfeld Difference C19      --C20      .       0.22 Ang.
PLAT234_ALERT_4_C Large Hirshfeld Difference Pd6      --N12      .       0.19 Ang.
PLAT241_ALERT_2_C High   'MainMol' Ueq as Compared to Neighbors of         C3 Check
PLAT241_ALERT_2_C High   'MainMol' Ueq as Compared to Neighbors of        C14 Check
PLAT241_ALERT_2_C High   'MainMol' Ueq as Compared to Neighbors of        C15 Check
PLAT241_ALERT_2_C High   'MainMol' Ueq as Compared to Neighbors of        C18 Check
PLAT241_ALERT_2_C High   'MainMol' Ueq as Compared to Neighbors of        C32 Check
PLAT241_ALERT_2_C High   'MainMol' Ueq as Compared to Neighbors of        C45 Check
PLAT242_ALERT_2_C Low    'MainMol' Ueq as Compared to Neighbors of         N3 Check
PLAT242_ALERT_2_C Low    'MainMol' Ueq as Compared to Neighbors of        C20 Check
PLAT242_ALERT_2_C Low    'MainMol' Ueq as Compared to Neighbors of        Pd5 Check
PLAT242_ALERT_2_C Low    'MainMol' Ueq as Compared to Neighbors of        Pd6 Check
PLAT242_ALERT_2_C Low    'MainMol' Ueq as Compared to Neighbors of        C43 Check
PLAT242_ALERT_2_C Low    'MainMol' Ueq as Compared to Neighbors of        C62 Check
PLAT242_ALERT_2_C Low    'MainMol' Ueq as Compared to Neighbors of        C77 Check
PLAT250_ALERT_2_C Large U3/U1 Ratio for Average U(i,j) Tensor ....        2.6 Note
PLAT250_ALERT_2_C Large U3/U1 Ratio for Average U(i,j) Tensor ....        2.7 Note
PLAT260_ALERT_2_C Large Average Ueq of Residue Including       Pd4      0.123 Check
PLAT260_ALERT_2_C Large Average Ueq of Residue Including       O1A      0.197 Check
PLAT260_ALERT_2_C Large Average Ueq of Residue Including      Cl1S      0.180 Check
PLAT260_ALERT_2_C Large Average Ueq of Residue Including      Cl4S      0.249 Check
PLAT260_ALERT_2_C Large Average Ueq of Residue Including       N1S      0.160 Check
PLAT260_ALERT_2_C Large Average Ueq of Residue Including       O4W      0.229 Check
PLAT260_ALERT_2_C Large Average Ueq of Residue Including       O5W      0.231 Check
```

```
PLAT260_ALERT_2_C Large Average Ueq of Residue Including       O6W      0.178 Check
PLAT260_ALERT_2_C Large Average Ueq of Residue Including       O8W      0.265 Check
PLAT260_ALERT_2_C Large Average Ueq of Residue Including       O3W      0.150 Check
PLAT260_ALERT_2_C Large Average Ueq of Residue Including       O7W      0.256 Check
PLAT260_ALERT_2_C Large Average Ueq of Residue Including       O9W      0.210 Check
PLAT260_ALERT_2_C Large Average Ueq of Residue Including      O10W      0.177 Check
PLAT260_ALERT_2_C Large Average Ueq of Residue Including      O11W      0.257 Check
PLAT309_ALERT_2_C Single Bonded Oxygen (C-O > 1.3 Ang) ...........        O1A Check
PLAT309_ALERT_2_C Single Bonded Oxygen (C-O > 1.3 Ang) ...........        O3A Check
PLAT336_ALERT_2_C Long Bond Distance for ..... C1S      -Cl1S          1.910 Ang.
PLAT336_ALERT_2_C Long Bond Distance for ..... C1S      -Cl2S          1.890 Ang.
PLAT336_ALERT_2_C Long Bond Distance for ..... C1S      -Cl3S          1.890 Ang.
PLAT342_ALERT_3_C Low Bond Precision on  C-C Bonds ...............     0.0172 Ang.
PLAT420_ALERT_2_C D-H Bond Without Acceptor  N7       --H7       .     Please Check
PLAT767_ALERT_4_C INS Embedded LIST 6 Instruction Should be LIST 4     Please Check
PLAT906_ALERT_3_C Large K Value in the Analysis of Variance ......     20.063 Check
PLAT906_ALERT_3_C Large K Value in the Analysis of Variance ......      4.189 Check
PLAT906_ALERT_3_C Large K Value in the Analysis of Variance ......      2.135 Check
PLAT911_ALERT_3_C Missing FCF Refl Between Thmin & STh/L=    0.600        134 Report
PLAT918_ALERT_3_C Reflection(s) with I(obs) much Smaller I(calc) .          4 Check
PLAT971_ALERT_2_C Check Calcd Resid. Dens.  0.95Ang From N5            2.50 eA-3
```

Author Response: The atom type is correct and there is no evidence of twinning.

```
PLAT971_ALERT_2_C Check Calcd Resid. Dens.  1.15Ang From Pd2            2.42 eA-3
```

Author Response: The atom type is correct and there is no evidence of twinning.

```
PLAT971_ALERT_2_C Check Calcd Resid. Dens.  0.92Ang From Pd4            2.39 eA-3
```

Author Response: The atom type is correct and there is no evidence of twinning.

```
PLAT971_ALERT_2_C Check Calcd Resid. Dens.  1.08Ang From Pd4            2.37 eA-3
```

Author Response: The atom type is correct and there is no evidence of twinning.

```
PLAT971_ALERT_2_C Check Calcd Resid. Dens.  1.12Ang From Pd2            2.37 eA-3
```

Author Response: The atom type is correct and there is no evidence of twinning.

```
PLAT971_ALERT_2_C Check Calcd Resid. Dens.  0.61Ang From C62            2.29 eA-3
```

Author Response: The atom type is correct and there is no evidence of twinning.

```
PLAT971_ALERT_2_C Check Calcd Resid. Dens.  1.01Ang From Pd3            2.27 eA-3
```

Author Response: The atom type is correct and there is no evidence of twinning.

```
PLAT971_ALERT_2_C Check Calcd Resid. Dens.  1.11Ang From Pd3            2.24 eA-3
```

Author Response: The atom type is correct and there is no evidence of twinning.

```
PLAT971_ALERT_2_C Check Calcd Resid. Dens.  0.70Ang From Pd4            2.24 eA-3
```

Author Response: The atom type is correct and there is no evidence of twinning.

```
PLAT971_ALERT_2_C Check Calcd Resid. Dens.  0.78Ang From Pd3            2.23 eA-3
```

Author Response: The atom type is correct and there is no evidence of twinning.

```
PLAT971_ALERT_2_C Check Calcd Resid. Dens.  0.82Ang From Pd4            2.15 eA-3
```

Author Response: The atom type is correct and there is no evidence of twinning.

```
PLAT971_ALERT_2_C Check Calcd Resid. Dens.  1.06Ang From Pd5            2.05 eA-3
```

Author Response: The atom type is correct and there is no evidence of twinning.

```
PLAT971_ALERT_2_C Check Calcd Resid. Dens.  0.65Ang From Pd3            2.04 eA-3
```

Author Response: The atom type is correct and there is no evidence of twinning.

```
PLAT971_ALERT_2_C Check Calcd Resid. Dens.  1.05Ang From Pd6            1.76 eA-3
```

Author Response: The atom type is correct and there is no evidence of twinning.

```
PLAT972_ALERT_2_C Check Calcd Resid. Dens.  1.04Ang From Pd1           -2.24 eA-3
PLAT972_ALERT_2_C Check Calcd Resid. Dens.  1.16Ang From Pd1           -2.11 eA-3
PLAT972_ALERT_2_C Check Calcd Resid. Dens.  1.10Ang From Pd1           -2.00 eA-3
PLAT972_ALERT_2_C Check Calcd Resid. Dens.  0.95Ang From Pd5           -1.94 eA-3
PLAT972_ALERT_2_C Check Calcd Resid. Dens.  0.92Ang From Pd4           -1.91 eA-3
PLAT972_ALERT_2_C Check Calcd Resid. Dens.  1.07Ang From Pd2           -1.87 eA-3
PLAT972_ALERT_2_C Check Calcd Resid. Dens.  1.03Ang From Pd3           -1.81 eA-3
PLAT972_ALERT_2_C Check Calcd Resid. Dens.  0.95Ang From Pd2           -1.79 eA-3
PLAT972_ALERT_2_C Check Calcd Resid. Dens.  0.94Ang From Pd4           -1.71 eA-3
PLAT972_ALERT_2_C Check Calcd Resid. Dens.  1.00Ang From Pd4           -1.68 eA-3
PLAT972_ALERT_2_C Check Calcd Resid. Dens.  1.01Ang From Pd2           -1.60 eA-3
PLAT972_ALERT_2_C Check Calcd Resid. Dens.  1.00Ang From Pd1           -1.59 eA-3
PLAT972_ALERT_2_C Check Calcd Resid. Dens.  1.10Ang From Pd5           -1.59 eA-3
PLAT972_ALERT_2_C Check Calcd Resid. Dens.  1.84Ang From Pd1           -1.51 eA-3
PLAT977_ALERT_2_C Check Negative Difference Density on H31       .     -0.36 eA-3
PLAT977_ALERT_2_C Check Negative Difference Density on H38       .     -0.40 eA-3
```

Alert level G

```
FORMU01_ALERT_1_G  There is a discrepancy between the atom counts in the
            _chemical_formula_sum and _chemical_formula_moiety. This is
            usually due to the moiety formula being in the wrong format.
            Atom count from _chemical_formula_sum:   C89.55999 H87.78999 Cl13.75 N
            Atom count from _chemical_formula_moiety:C89.62299 H85.815 Cl13.74900
FORMU01_ALERT_2_G  There is a discrepancy between the atom counts in the
            _chemical_formula_sum and the formula from the _atom_site* data.
            Atom count from _chemical_formula_sum:C89.55999 H87.78999 Cl13.75 N12.
            Atom count from the _atom_site data:  C89.64330 H87.83329 Cl13.74989 N
CELLZ01_ALERT_1_G Difference between formula and atom_site contents detected.
CELLZ01_ALERT_1_G ALERT: Large difference may be due to a
            symmetry error - see SYMMG tests
           From the CIF: _cell_formula_units_Z    4
           From the CIF: _chemical_formula_sum  C89.56 H87.79 Cl13.75 N12.66 O6.64
           TEST: Compare cell contents of formula and atom_site data

           atom    Z*formula  cif sites diff
           C        358.24    358.57   -0.33
           H        351.16    351.33   -0.17
           Cl        55.00     55.00    0.00
           N         50.64     50.68   -0.04
           O         26.56     26.62   -0.06
           Pd        24.00     24.00    0.00
PLAT002_ALERT_2_G Number of Distance or Angle Restraints on AtSite         22 Note
PLAT003_ALERT_2_G Number of Uiso or Uij Restrained non-H Atoms ...         36 Report
PLAT007_ALERT_5_G Number of Unrefined Donor-H Atoms .............         12 Report
PLAT045_ALERT_1_G Calculated and Reported Z Differ by a Factor ...          2 Check
PLAT072_ALERT_2_G SHELXL First  Parameter in WGHT  Unusually Large       0.20 Report
PLAT172_ALERT_4_G The CIF-Embedded .res File Contains DFIX Records         27 Report
PLAT174_ALERT_4_G The CIF-Embedded .res File Contains FLAT Records         20 Report
PLAT178_ALERT_4_G The CIF-Embedded .res File Contains SIMU Records          6 Report
PLAT186_ALERT_4_G The CIF-Embedded .res File Contains ISOR Records          6 Report
PLAT187_ALERT_4_G The CIF-Embedded .res File Contains RIGU Records          8 Report
PLAT188_ALERT_3_G A Non-default SIMU Restraint Value has been used     0.0200 Report
PLAT188_ALERT_3_G A Non-default SIMU Restraint Value has been used     0.0200 Report
PLAT188_ALERT_3_G A Non-default SIMU Restraint Value has been used     0.0200 Report
PLAT188_ALERT_3_G A Non-default SIMU Restraint Value has been used     0.0200 Report
PLAT190_ALERT_3_G A Non-default RIGU Restraint Value for First Par     0.0020 Report
PLAT190_ALERT_3_G A Non-default RIGU Restraint Value for SecondPar     0.0020 Report
PLAT190_ALERT_3_G A Non-default RIGU Restraint Value for First Par     0.0020 Report
PLAT190_ALERT_3_G A Non-default RIGU Restraint Value for SecondPar     0.0020 Report
PLAT190_ALERT_3_G A Non-default RIGU Restraint Value for First Par     0.0020 Report
PLAT190_ALERT_3_G A Non-default RIGU Restraint Value for SecondPar     0.0020 Report
PLAT190_ALERT_3_G A Non-default RIGU Restraint Value for First Par     0.0020 Report
PLAT190_ALERT_3_G A Non-default RIGU Restraint Value for SecondPar     0.0020 Report
PLAT190_ALERT_3_G A Non-default RIGU Restraint Value for First Par     0.0020 Report
PLAT190_ALERT_3_G A Non-default RIGU Restraint Value for SecondPar     0.0020 Report
PLAT190_ALERT_3_G A Non-default RIGU Restraint Value for First Par     0.0020 Report
PLAT190_ALERT_3_G A Non-default RIGU Restraint Value for SecondPar     0.0020 Report
PLAT190_ALERT_3_G A Non-default RIGU Restraint Value for First Par     0.0020 Report
PLAT190_ALERT_3_G A Non-default RIGU Restraint Value for SecondPar     0.0020 Report
PLAT190_ALERT_3_G A Non-default RIGU Restraint Value for First Par     0.0020 Report
PLAT190_ALERT_3_G A Non-default RIGU Restraint Value for SecondPar     0.0020 Report
PLAT300_ALERT_4_G Atom Site Occupancy of Cl1S       Constrained at       0.25 Check
PLAT300_ALERT_4_G Atom Site Occupancy of Cl2S       Constrained at       0.25 Check
PLAT300_ALERT_4_G Atom Site Occupancy of Cl3S       Constrained at       0.25 Check
PLAT300_ALERT_4_G Atom Site Occupancy of C1S        Constrained at       0.25 Check
```

```
PLAT300_ALERT_4_G Atom Site Occupancy of H1S        Constrained at       0.25 Check
PLAT300_ALERT_4_G Atom Site Occupancy of Cl4S       Constrained at     0.3333 Check
PLAT300_ALERT_4_G Atom Site Occupancy of Cl5S       Constrained at     0.3333 Check
PLAT300_ALERT_4_G Atom Site Occupancy of Cl6S       Constrained at     0.3333 Check
PLAT300_ALERT_4_G Atom Site Occupancy of C2S        Constrained at     0.3333 Check
PLAT300_ALERT_4_G Atom Site Occupancy of H2S        Constrained at     0.3333 Check
PLAT300_ALERT_4_G Atom Site Occupancy of O4W        Constrained at        0.5 Check
PLAT300_ALERT_4_G Atom Site Occupancy of O5W        Constrained at        0.5 Check
PLAT300_ALERT_4_G Atom Site Occupancy of O6W        Constrained at        0.5 Check
PLAT300_ALERT_4_G Atom Site Occupancy of O7W        Constrained at     0.3333 Check
PLAT300_ALERT_4_G Atom Site Occupancy of O11W       Constrained at     0.3333 Check
PLAT302_ALERT_4_G Anion/Solvent/Minor-Residue Disorder (Resd  3  )       100% Note
PLAT302_ALERT_4_G Anion/Solvent/Minor-Residue Disorder (Resd  4  )       100% Note
PLAT302_ALERT_4_G Anion/Solvent/Minor-Residue Disorder (Resd  5  )       100% Note
PLAT302_ALERT_4_G Anion/Solvent/Minor-Residue Disorder (Resd  6  )       100% Note
PLAT302_ALERT_4_G Anion/Solvent/Minor-Residue Disorder (Resd  7  )       100% Note
PLAT302_ALERT_4_G Anion/Solvent/Minor-Residue Disorder (Resd  8  )       100% Note
PLAT302_ALERT_4_G Anion/Solvent/Minor-Residue Disorder (Resd  9  )       100% Note
PLAT302_ALERT_4_G Anion/Solvent/Minor-Residue Disorder (Resd 11  )       100% Note
PLAT302_ALERT_4_G Anion/Solvent/Minor-Residue Disorder (Resd 12  )       100% Note
PLAT302_ALERT_4_G Anion/Solvent/Minor-Residue Disorder (Resd 13  )       100% Note
PLAT302_ALERT_4_G Anion/Solvent/Minor-Residue Disorder (Resd 14  )       100% Note
PLAT302_ALERT_4_G Anion/Solvent/Minor-Residue Disorder (Resd 15  )       100% Note
PLAT302_ALERT_4_G Anion/Solvent/Minor-Residue Disorder (Resd 16  )       100% Note
PLAT302_ALERT_4_G Anion/Solvent/Minor-Residue Disorder (Resd 17  )       100% Note
PLAT304_ALERT_4_G Non-Integer Number of Atoms in ..... (Resd  3  )       6.82 Check
PLAT304_ALERT_4_G Non-Integer Number of Atoms in ..... (Resd  4  )       1.25 Check
PLAT304_ALERT_4_G Non-Integer Number of Atoms in ..... (Resd  5  )       1.67 Check
PLAT304_ALERT_4_G Non-Integer Number of Atoms in ..... (Resd  6  )       4.02 Check
PLAT304_ALERT_4_G Non-Integer Number of Atoms in ..... (Resd  7  )       0.50 Check
PLAT304_ALERT_4_G Non-Integer Number of Atoms in ..... (Resd  8  )       0.50 Check
PLAT304_ALERT_4_G Non-Integer Number of Atoms in ..... (Resd  9  )       0.50 Check
PLAT304_ALERT_4_G Non-Integer Number of Atoms in ..... (Resd 11  )       0.33 Check
PLAT304_ALERT_4_G Non-Integer Number of Atoms in ..... (Resd 12  )       0.33 Check
PLAT304_ALERT_4_G Non-Integer Number of Atoms in ..... (Resd 13  )       0.27 Check
PLAT304_ALERT_4_G Non-Integer Number of Atoms in ..... (Resd 14  )       0.33 Check
PLAT304_ALERT_4_G Non-Integer Number of Atoms in ..... (Resd 15  )       0.35 Check
PLAT304_ALERT_4_G Non-Integer Number of Atoms in ..... (Resd 16  )       0.35 Check
PLAT304_ALERT_4_G Non-Integer Number of Atoms in ..... (Resd 17  )       0.33 Check
PLAT311_ALERT_2_G Isolated Disordered Oxygen Atom (No H's ?) .....        O4W Check
PLAT311_ALERT_2_G Isolated Disordered Oxygen Atom (No H's ?) .....        O5W Check
PLAT311_ALERT_2_G Isolated Disordered Oxygen Atom (No H's ?) .....        O6W Check
PLAT311_ALERT_2_G Isolated Disordered Oxygen Atom (No H's ?) .....        O1W Check
PLAT311_ALERT_2_G Isolated Disordered Oxygen Atom (No H's ?) .....        O2W Check
PLAT311_ALERT_2_G Isolated Disordered Oxygen Atom (No H's ?) .....        O3W Check
PLAT311_ALERT_2_G Isolated Disordered Oxygen Atom (No H's ?) .....        O7W Check
PLAT311_ALERT_2_G Isolated Disordered Oxygen Atom (No H's ?) .....        O9W Check
PLAT311_ALERT_2_G Isolated Disordered Oxygen Atom (No H's ?) .....       O10W Check
PLAT311_ALERT_2_G Isolated Disordered Oxygen Atom (No H's ?) .....       O11W Check
PLAT398_ALERT_2_G Deviating  C-O-C   Angle From 120 for O2A      .      106.4 Degree
PLAT720_ALERT_4_G Number of Unusual/Non-Standard Labels .........           5 Note
PLAT793_ALERT_4_G Model has Chirality at N1          (Centro SPGR)          S Verify
PLAT793_ALERT_4_G Model has Chirality at N2          (Centro SPGR)          R Verify
PLAT793_ALERT_4_G Model has Chirality at N3          (Centro SPGR)          S Verify
PLAT793_ALERT_4_G Model has Chirality at N4          (Centro SPGR)          R Verify
PLAT793_ALERT_4_G Model has Chirality at N5          (Centro SPGR)          S Verify
PLAT793_ALERT_4_G Model has Chirality at N6          (Centro SPGR)          R Verify
```

```
PLAT793_ALERT_4_G Model has Chirality at N7          (Centro SPGR)          R Verify
PLAT793_ALERT_4_G Model has Chirality at N8          (Centro SPGR)          S Verify
PLAT793_ALERT_4_G Model has Chirality at N9          (Centro SPGR)          S Verify
PLAT793_ALERT_4_G Model has Chirality at N10         (Centro SPGR)          R Verify
PLAT793_ALERT_4_G Model has Chirality at N11         (Centro SPGR)          R Verify
PLAT793_ALERT_4_G Model has Chirality at N12         (Centro SPGR)          S Verify
PLAT794_ALERT_5_G Tentative Bond Valency for Pd3       (II)      .       2.16 Info
PLAT794_ALERT_5_G Tentative Bond Valency for Pd4       (II)      .       1.91 Info
PLAT794_ALERT_5_G Tentative Bond Valency for Pd5       (II)      .       2.27 Info
PLAT794_ALERT_5_G Tentative Bond Valency for Pd6       (II)      .       2.39 Info
PLAT860_ALERT_3_G Number of Least-Squares Restraints .............        594 Note
PLAT883_ALERT_1_G No Info/Value for _atom_sites_solution_primary .     Please Do !
PLAT910_ALERT_3_G Missing # of FCF Reflection(s) Below Theta(Min).          4 Note
PLAT912_ALERT_4_G Missing # of FCF Reflections Above STh/L=  0.600         21 Note
PLAT913_ALERT_3_G Missing # of Very Strong Reflections in FCF ....          3 Note
PLAT933_ALERT_2_G Number of HKL-OMIT Records in Embedded .res File         13 Note
PLAT941_ALERT_3_G Average HKL Measurement Multiplicity ...........        4.1 Low
PLAT978_ALERT_2_G Number C-C Bonds with Positive Residual Density.          0 Info
```

---

3 **ALERT level A** = Most likely a serious problem - resolve or explain
14 **ALERT level B** = A potentially serious problem, consider carefully
93 **ALERT level C** = Check. Ensure it is not caused by an omission or oversight
113 **ALERT level G** = General information/check it is not something unexpected

7 ALERT type 1 CIF construction/syntax error, inconsistent or missing data
103 ALERT type 2 Indicator that the structure model may be wrong or deficient
33 ALERT type 3 Indicator that the structure quality may be low
75 ALERT type 4 Improvement, methodology, query or suggestion
5 ALERT type 5 Informative message, check

It is advisable to attempt to resolve as many as possible of the alerts in all categories. Often the minor alerts point to easily fixed oversights, errors and omissions in your CIF or refinement strategy, so attention to these fine details can be worthwhile. In order to resolve some of the more serious problems it may be necessary to carry out additional measurements or structure refinements. However, the purpose of your study may justify the reported deviations and the more serious of these should normally be commented upon in the discussion or experimental section of a paper or in the "special_details" fields of the CIF. checkCIF was carefully designed to identify outliers and unusual parameters, but every test has its limitations and alerts that are not important in a particular case may appear. Conversely, the absence of alerts does not guarantee there are no aspects of the results needing attention. It is up to the individual to critically assess their own results and, if necessary, seek expert advice.

Publication of your CIF in IUCr journals

A basic structural check has been run on your CIF. These basic checks will be run on all CIFs submitted for publication in IUCr journals (*Acta Crystallographica*, *Journal of Applied Crystallography*, *Journal of Synchrotron Radiation*); however, if you intend to submit to *Acta Crystallographica Section C* or *E* or *IUCrData*, you should make sure that full publication checks are run on the final version of your CIF prior to submission.

Publication of your CIF in other journals

Please refer to the *Notes for Authors* of the relevant journal for any special instructions relating to CIF submission.

PLATON version of 28/11/2022; check.def file version of 28/11/2022

Datablock HMF-CHCl3@MMF - ellipsoid plot

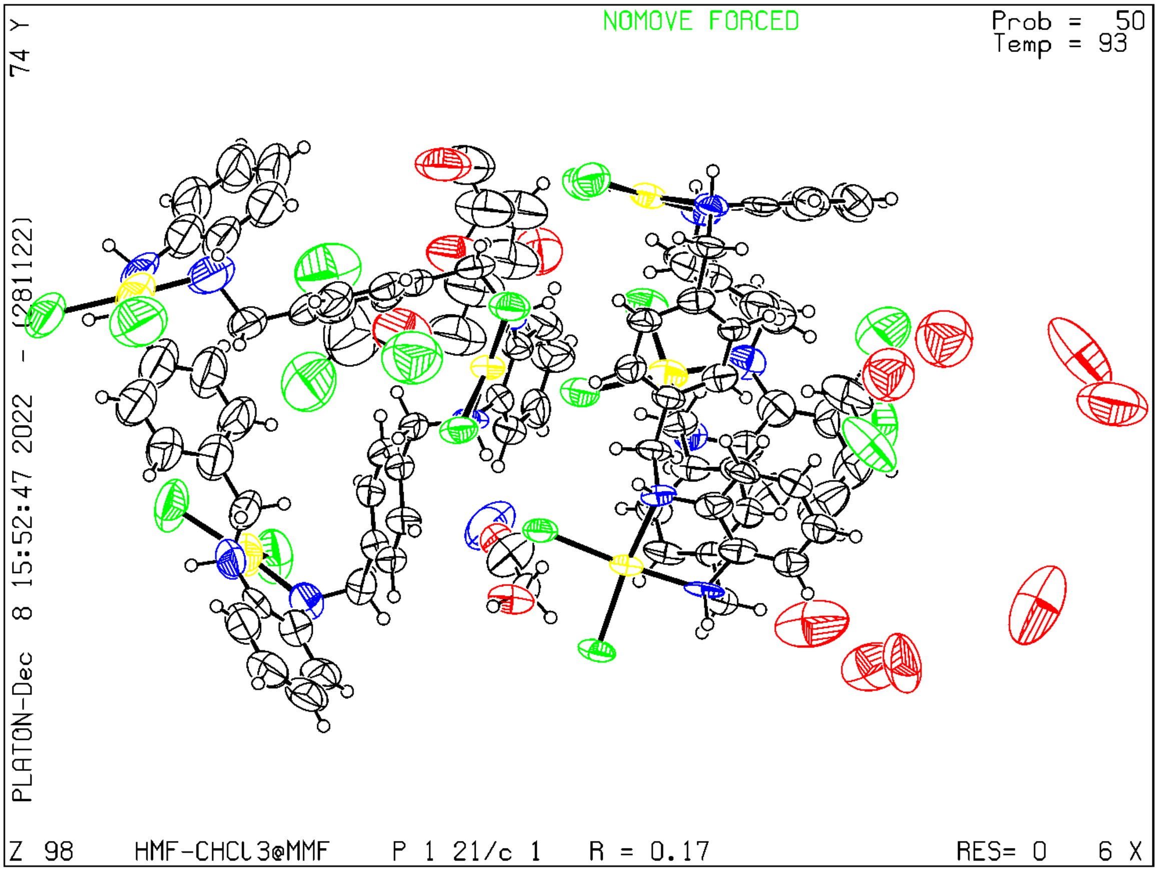

Supplement: Supplementary file 2 — Supporting Information [file SMLL-20-2405507-s001.zip › HMF-CHCl3@MMF_checkcif.pdf]
