# Supplementary material for: Porous Supramolecular Crystalline Probe that Detects Non‐Covalent Interactions Involved in Molecular Recognition of Furanic Compounds
Source: Small. 2024 Jul 30;20(49):2405507. doi: 10.1002/smll.202405507 (PMC11618713; doi:10.1002/smll.202405507)

checkCIF/PLATON report

Structure factors have been supplied for datablock(s) HMF-MeCN@MMF

THIS REPORT IS FOR GUIDANCE ONLY. IF USED AS PART OF A REVIEW PROCEDURE FOR PUBLICATION, IT SHOULD NOT REPLACE THE EXPERTISE OF AN EXPERIENCED CRYSTALLOGRAPHIC REFEREE.

No syntax errors found. CIF dictionary Interpreting this report

Datablock: HMF-MeCN@MMF

```
Bond precision:   C-C = 0.0092 A           Wavelength=1.54184

Cell:            a=19.68940(11)    b=52.7043(4)       c=14.27080(7)
                 alpha=90          beta=90.9211(5)    gamma=90
Temperature:     93 K
```

	Calculated	Reported
Volume	14807.12(16)	14807.13(16)
Space group	P 21/c	P 1 21/c 1
Hall group	-P 2ybc	-P 2ybc
Moiety formula	8(C42 H42 Cl6 N6 Pd3), 2.932(C6 H4 O2), 3.06(C5 H2 O2), 7(C2 H3	2(C42 H42 Cl6 N6 Pd3), 1.75(C2 H3 N), 4.25(O), 0.765(C5 H2 O2),
Sum formula	C382.89 H374.85 Cl48 N55 O28.98 Pd24	C95.72 H93.71 Cl12 N13.75 O7.24 Pd6
Mr	10465.89	2616.44
Dx,g cm-3	1.174	1.174
Z	1	4
Mu (mm-1)	8.086	8.086
F000	5209.1	5209.0
F000’	5240.62	
h,k,lmax	23,63,17	23,63,17
Nref	27124	27086
Tmin,Tmax	0.188,0.491	0.234,1.000
Tmin’	0.085	

```
Correction method= # Reported T Limits: Tmin=0.234 Tmax=1.000
AbsCorr = MULTI-SCAN

Data completeness= 0.999           Theta(max)= 68.249
```

R(reflections)= 0.0859(23955) wR2(reflections)= 0.2507(27086)

S = 1.103 Npar= 1157

The following ALERTS were generated. Each ALERT has the format
test-name_ALERT_alert-type_alert-level.
Click on the hyperlinks for more details of the test.

Alert level A

PLAT602_ALERT_2_A Solvent Accessible VOID(S) in Structure .... ! Check

Author Response: Some solvents in the large pore could not be located due to severe disordering.

Alert level B

PLAT306_ALERT_2_B Isolated Oxygen Atom (H-atoms Missing ?) .... O2W Check

Author Response: Hydrogen atoms of water molecules and OH groups of the guests could not be located in the difference electron density maps.

PLAT971_ALERT_2_B Check Calcd Resid. Dens. 0.51Ang From C20 2.83 eA-3

Author Response: The atom type is correct and there is no evidence of twinning.

PLAT971_ALERT_2_B Check Calcd Resid. Dens. 0.73Ang From C62 2.73 eA-3

Author Response: The atom type is correct and there is no evidence of twinning.

PLAT971_ALERT_2_B Check Calcd Resid. Dens. 0.99Ang From N11 2.55 eA-3

Author Response: The atom type is correct and there is no evidence of twinning.

PLAT972_ALERT_2_B Check Calcd Resid. Dens. 0.82Ang From Pd6 -3.03 eA-3

Author Response: The atom type is correct and there is no evidence of twinning. The large residual density on Pd atoms may be Due to an anomalous dispersion effect and has no chemical significance.

PLAT972_ALERT_2_B Check Calcd Resid. Dens. 0.48Ang From Pd6 -2.81 eA-3

Author Response: The atom type is correct and there is no evidence of twinning. The large residual density on Pd atoms may be Due to an anomalous dispersion effect and has no chemical significance.

```
PLAT972_ALERT_2_B Check Calcd Resid. Dens.  0.88Ang From Pd6           -2.58 eA-3
```

Author Response: The atom type is correct and there is no evidence of twinning. The large residual density on Pd atoms may be Due to an anomalous dispersion effect and has no chemical significance.

```
PLAT973_ALERT_2_B Check Calcd Positive Resid. Density on       Pd1       1.53 eA-3
```

Author Response: The atom type is correct and there is no evidence of twinning. The large residual density on Pd atoms may be Due to an anomalous dispersion effect and has no chemical significance.

Alert level C

```
PLAT041_ALERT_1_C Calc. and Reported SumFormula    Strings  Differ     Please Check
PLAT213_ALERT_2_C Atom N11             has ADP max/min Ratio .....         3.4 prolat
PLAT220_ALERT_2_C NonSolvent   Resd 2  C   Ueq(max)/Ueq(min) Range         4.7 Ratio
PLAT220_ALERT_2_C NonSolvent   Resd 2 Cl   Ueq(max)/Ueq(min) Range         3.5 Ratio
PLAT220_ALERT_2_C NonSolvent   Resd 2  N   Ueq(max)/Ueq(min) Range         3.7 Ratio
PLAT220_ALERT_2_C NonSolvent   Resd 2 Pd   Ueq(max)/Ueq(min) Range         3.1 Ratio
PLAT222_ALERT_3_C NonSolvent Resd 2  H   Uiso(max)/Uiso(min) Range         4.8 Ratio
PLAT230_ALERT_2_C Hirshfeld Test Diff for    N10      --C62      .         6.4 s.u.
PLAT234_ALERT_4_C Large Hirshfeld Difference Pd6      --N11      .        0.20 Ang.
PLAT241_ALERT_2_C High   'MainMol' Ueq as Compared to Neighbors of         C31 Check
PLAT242_ALERT_2_C Low    'MainMol' Ueq as Compared to Neighbors of         Pd6 Check
PLAT242_ALERT_2_C Low    'MainMol' Ueq as Compared to Neighbors of         C62 Check
PLAT250_ALERT_2_C Large U3/U1 Ratio for Average U(i,j) Tensor ....         2.3 Note
PLAT250_ALERT_2_C Large U3/U1 Ratio for Average U(i,j) Tensor ....         2.5 Note
PLAT260_ALERT_2_C Large Average Ueq of Residue Including       O1A       0.266 Check
PLAT260_ALERT_2_C Large Average Ueq of Residue Including       O1B       0.254 Check
PLAT260_ALERT_2_C Large Average Ueq of Residue Including       N2S       0.190 Check
PLAT260_ALERT_2_C Large Average Ueq of Residue Including       O1W       0.240 Check
PLAT260_ALERT_2_C Large Average Ueq of Residue Including       O2W       0.216 Check
PLAT260_ALERT_2_C Large Average Ueq of Residue Including       O5W       0.289 Check
PLAT260_ALERT_2_C Large Average Ueq of Residue Including       O9W       0.179 Check
PLAT260_ALERT_2_C Large Average Ueq of Residue Including       O3W       0.264 Check
PLAT260_ALERT_2_C Large Average Ueq of Residue Including       O4W       0.240 Check
PLAT260_ALERT_2_C Large Average Ueq of Residue Including       O6W       0.186 Check
PLAT260_ALERT_2_C Large Average Ueq of Residue Including       O7W       0.252 Check
PLAT260_ALERT_2_C Large Average Ueq of Residue Including       O8W       0.281 Check
PLAT309_ALERT_2_C Single Bonded Oxygen (C-O > 1.3 Ang) ..........      O2A Check
PLAT342_ALERT_3_C Low Bond Precision on  C-C Bonds ..............    0.00925 Ang.
PLAT420_ALERT_2_C D-H Bond Without Acceptor  N7       --H7       .     Please Check
PLAT767_ALERT_4_C INS Embedded LIST 6 Instruction Should be LIST 4     Please Check
```

```
PLAT906_ALERT_3_C Large K Value in the Analysis of Variance ......      5.888 Check
PLAT911_ALERT_3_C Missing FCF Refl Between Thmin & STh/L=    0.600         27 Report
PLAT918_ALERT_3_C Reflection(s) with I(obs) much Smaller I(calc) .          7 Check
PLAT971_ALERT_2_C Check Calcd Resid. Dens.  0.77Ang From N11             2.09 eA-3
```

Author Response: The atom type is correct and there is no evidence of twinning.

```
PLAT971_ALERT_2_C Check Calcd Resid. Dens.  1.05Ang From Pd6             2.05 eA-3
```

Author Response: The atom type is correct and there is no evidence of twinning.

```
PLAT971_ALERT_2_C Check Calcd Resid. Dens.  0.87Ang From Pd4             1.79 eA-3
```

Author Response: The atom type is correct and there is no evidence of twinning.

```
PLAT971_ALERT_2_C Check Calcd Resid. Dens.  1.07Ang From Pd1             1.77 eA-3
```

Author Response: The atom type is correct and there is no evidence of twinning.

```
PLAT971_ALERT_2_C Check Calcd Resid. Dens.  1.11Ang From Pd2             1.66 eA-3
```

Author Response: The atom type is correct and there is no evidence of twinning.

```
PLAT972_ALERT_2_C Check Calcd Resid. Dens.  0.73Ang From Pd5            -2.09 eA-3
```

Author Response: The atom type is correct and there is no evidence of twinning. The large residual density on Pd atoms may be Due to an anomalous dispersion effect and has no chemical significance.

Alert level G

```
FORMU01_ALERT_1_G  There is a discrepancy between the atom counts in the
            _chemical_formula_sum and _chemical_formula_moiety. This is
            usually due to the moiety formula being in the wrong format.
            Atom count from _chemical_formula_sum:   C95.72 H93.71 Cl12 N13.75 O7.
            Atom count from _chemical_formula_moiety:C95.723 H93.71199 Cl12 N13.75
PLAT002_ALERT_2_G Number of Distance or Angle Restraints on AtSite         15 Note
PLAT003_ALERT_2_G Number of Uiso or Uij Restrained non-H Atoms ...         29 Report
PLAT007_ALERT_5_G Number of Unrefined Donor-H Atoms .............          12 Report
PLAT045_ALERT_1_G Calculated and Reported Z Differ by a Factor ...      0.250 Check
PLAT072_ALERT_2_G SHELXL First  Parameter in WGHT  Unusually Large       0.12 Report
PLAT083_ALERT_2_G SHELXL Second Parameter in WGHT  Unusually Large     122.00 Why ?
PLAT142_ALERT_4_G s.u. on b - Axis Small or Missing .............    0.00040 Ang.
PLAT143_ALERT_4_G s.u. on c - Axis Small or Missing .............    0.00007 Ang.
PLAT172_ALERT_4_G The CIF-Embedded .res File Contains DFIX Records         27 Report
PLAT174_ALERT_4_G The CIF-Embedded .res File Contains FLAT Records         12 Report
PLAT178_ALERT_4_G The CIF-Embedded .res File Contains SIMU Records          9 Report
```

```
PLAT186_ALERT_4_G The CIF-Embedded .res File Contains ISOR Records          9 Report
PLAT187_ALERT_4_G The CIF-Embedded .res File Contains RIGU Records         12 Report
PLAT188_ALERT_3_G A Non-default SIMU Restraint Value has been used     0.0200 Report
PLAT188_ALERT_3_G A Non-default SIMU Restraint Value has been used     0.0050 Report
PLAT188_ALERT_3_G A Non-default SIMU Restraint Value has been used     0.0050 Report
PLAT188_ALERT_3_G A Non-default SIMU Restraint Value has been used     0.0200 Report
PLAT188_ALERT_3_G A Non-default SIMU Restraint Value has been used     0.0050 Report
PLAT190_ALERT_3_G A Non-default RIGU Restraint Value for First Par     0.0020 Report
PLAT190_ALERT_3_G A Non-default RIGU Restraint Value for SecondPar     0.0020 Report
PLAT190_ALERT_3_G A Non-default RIGU Restraint Value for First Par     0.0005 Report
PLAT190_ALERT_3_G A Non-default RIGU Restraint Value for SecondPar     0.0005 Report
PLAT190_ALERT_3_G A Non-default RIGU Restraint Value for First Par     0.0005 Report
PLAT190_ALERT_3_G A Non-default RIGU Restraint Value for SecondPar     0.0005 Report
PLAT190_ALERT_3_G A Non-default RIGU Restraint Value for First Par     0.0020 Report
PLAT190_ALERT_3_G A Non-default RIGU Restraint Value for SecondPar     0.0020 Report
PLAT190_ALERT_3_G A Non-default RIGU Restraint Value for First Par     0.0005 Report
PLAT190_ALERT_3_G A Non-default RIGU Restraint Value for SecondPar     0.0005 Report
PLAT190_ALERT_3_G A Non-default RIGU Restraint Value for First Par     0.0020 Report
PLAT190_ALERT_3_G A Non-default RIGU Restraint Value for SecondPar     0.0020 Report
PLAT190_ALERT_3_G A Non-default RIGU Restraint Value for First Par     0.0020 Report
PLAT190_ALERT_3_G A Non-default RIGU Restraint Value for SecondPar     0.0020 Report
PLAT190_ALERT_3_G A Non-default RIGU Restraint Value for First Par     0.0020 Report
PLAT190_ALERT_3_G A Non-default RIGU Restraint Value for SecondPar     0.0020 Report
PLAT300_ALERT_4_G Atom Site Occupancy of N2S       Constrained at        0.75 Check
PLAT300_ALERT_4_G Atom Site Occupancy of C3S       Constrained at        0.75 Check
PLAT300_ALERT_4_G Atom Site Occupancy of C4S       Constrained at        0.75 Check
PLAT300_ALERT_4_G Atom Site Occupancy of H4SA      Constrained at        0.75 Check
PLAT300_ALERT_4_G Atom Site Occupancy of H4SB      Constrained at        0.75 Check
PLAT300_ALERT_4_G Atom Site Occupancy of H4SC      Constrained at        0.75 Check
PLAT300_ALERT_4_G Atom Site Occupancy of O1W       Constrained at        0.75 Check
PLAT300_ALERT_4_G Atom Site Occupancy of O5W       Constrained at         0.5 Check
PLAT300_ALERT_4_G Atom Site Occupancy of O9W       Constrained at         0.5 Check
PLAT300_ALERT_4_G Atom Site Occupancy of O3W       Constrained at      0.3333 Check
PLAT300_ALERT_4_G Atom Site Occupancy of O4W       Constrained at      0.3333 Check
PLAT300_ALERT_4_G Atom Site Occupancy of O6W       Constrained at        0.25 Check
PLAT300_ALERT_4_G Atom Site Occupancy of O7W       Constrained at        0.25 Check
PLAT300_ALERT_4_G Atom Site Occupancy of O8W       Constrained at      0.3333 Check
PLAT302_ALERT_4_G Anion/Solvent/Minor-Residue Disorder (Resd  3  )       100% Note
PLAT302_ALERT_4_G Anion/Solvent/Minor-Residue Disorder (Resd  4  )       100% Note
PLAT302_ALERT_4_G Anion/Solvent/Minor-Residue Disorder (Resd  6  )       100% Note
PLAT302_ALERT_4_G Anion/Solvent/Minor-Residue Disorder (Resd  7  )       100% Note
PLAT302_ALERT_4_G Anion/Solvent/Minor-Residue Disorder (Resd  9  )       100% Note
PLAT302_ALERT_4_G Anion/Solvent/Minor-Residue Disorder (Resd 10  )       100% Note
PLAT302_ALERT_4_G Anion/Solvent/Minor-Residue Disorder (Resd 11  )       100% Note
PLAT302_ALERT_4_G Anion/Solvent/Minor-Residue Disorder (Resd 12  )       100% Note
PLAT302_ALERT_4_G Anion/Solvent/Minor-Residue Disorder (Resd 13  )       100% Note
PLAT302_ALERT_4_G Anion/Solvent/Minor-Residue Disorder (Resd 14  )       100% Note
PLAT302_ALERT_4_G Anion/Solvent/Minor-Residue Disorder (Resd 15  )       100% Note
PLAT304_ALERT_4_G Non-Integer Number of Atoms in ..... (Resd  3  )       8.80 Check
PLAT304_ALERT_4_G Non-Integer Number of Atoms in ..... (Resd  4  )       6.88 Check
PLAT304_ALERT_4_G Non-Integer Number of Atoms in ..... (Resd  6  )       4.50 Check
PLAT304_ALERT_4_G Non-Integer Number of Atoms in ..... (Resd  7  )       0.75 Check
PLAT304_ALERT_4_G Non-Integer Number of Atoms in ..... (Resd  9  )       0.50 Check
PLAT304_ALERT_4_G Non-Integer Number of Atoms in ..... (Resd 10  )       0.50 Check
PLAT304_ALERT_4_G Non-Integer Number of Atoms in ..... (Resd 11  )       0.33 Check
PLAT304_ALERT_4_G Non-Integer Number of Atoms in ..... (Resd 12  )       0.33 Check
PLAT304_ALERT_4_G Non-Integer Number of Atoms in ..... (Resd 13  )       0.25 Check
```

```
PLAT304_ALERT_4_G Non-Integer Number of Atoms in ..... (Resd 14  )       0.25 Check
PLAT304_ALERT_4_G Non-Integer Number of Atoms in ..... (Resd 15  )       0.33 Check
PLAT311_ALERT_2_G Isolated Disordered Oxygen Atom (No H's ?) .....        O1W Check
PLAT311_ALERT_2_G Isolated Disordered Oxygen Atom (No H's ?) .....        O5W Check
PLAT311_ALERT_2_G Isolated Disordered Oxygen Atom (No H's ?) .....        O9W Check
PLAT311_ALERT_2_G Isolated Disordered Oxygen Atom (No H's ?) .....        O3W Check
PLAT311_ALERT_2_G Isolated Disordered Oxygen Atom (No H's ?) .....        O4W Check
PLAT311_ALERT_2_G Isolated Disordered Oxygen Atom (No H's ?) .....        O6W Check
PLAT311_ALERT_2_G Isolated Disordered Oxygen Atom (No H's ?) .....        O7W Check
PLAT311_ALERT_2_G Isolated Disordered Oxygen Atom (No H's ?) .....        O8W Check
PLAT315_ALERT_2_G Singly Bonded Carbon Detected (H-atoms Missing).        C1A Check
PLAT398_ALERT_2_G Deviating  C-O-C   Angle From 120 for O1A      .      105.9 Degree
PLAT398_ALERT_2_G Deviating  C-O-C   Angle From 120 for O1B      .      105.0 Degree
PLAT720_ALERT_4_G Number of Unusual/Non-Standard Labels .........          10 Note
PLAT790_ALERT_4_G Centre of Gravity not Within Unit Cell: Resd.  #          4 Note
              C5 H2 O2
PLAT790_ALERT_4_G Centre of Gravity not Within Unit Cell: Resd.  #         13 Note
              O
PLAT793_ALERT_4_G Model has Chirality at N1          (Centro SPGR)          R Verify
PLAT793_ALERT_4_G Model has Chirality at N2          (Centro SPGR)          S Verify
PLAT793_ALERT_4_G Model has Chirality at N3          (Centro SPGR)          R Verify
PLAT793_ALERT_4_G Model has Chirality at N4          (Centro SPGR)          S Verify
PLAT793_ALERT_4_G Model has Chirality at N5          (Centro SPGR)          R Verify
PLAT793_ALERT_4_G Model has Chirality at N6          (Centro SPGR)          S Verify
PLAT793_ALERT_4_G Model has Chirality at N7          (Centro SPGR)          S Verify
PLAT793_ALERT_4_G Model has Chirality at N8          (Centro SPGR)          R Verify
PLAT793_ALERT_4_G Model has Chirality at N9          (Centro SPGR)          R Verify
PLAT793_ALERT_4_G Model has Chirality at N10         (Centro SPGR)          S Verify
PLAT793_ALERT_4_G Model has Chirality at N11         (Centro SPGR)          S Verify
PLAT793_ALERT_4_G Model has Chirality at N12         (Centro SPGR)          R Verify
PLAT794_ALERT_5_G Tentative Bond Valency for Pd1       (II)      .       2.06 Info
PLAT794_ALERT_5_G Tentative Bond Valency for Pd2       (II)      .       2.13 Info
PLAT794_ALERT_5_G Tentative Bond Valency for Pd3       (II)      .       2.08 Info
PLAT794_ALERT_5_G Tentative Bond Valency for Pd4       (II)      .       2.05 Info
PLAT794_ALERT_5_G Tentative Bond Valency for Pd5       (II)      .       2.16 Info
PLAT794_ALERT_5_G Tentative Bond Valency for Pd6       (II)      .       2.47 Info
PLAT860_ALERT_3_G Number of Least-Squares Restraints ............         569 Note
PLAT883_ALERT_1_G No Info/Value for _atom_sites_solution_primary .     Please Do !
PLAT910_ALERT_3_G Missing # of FCF Reflection(s) Below Theta(Min).          3 Note
PLAT912_ALERT_4_G Missing # of FCF Reflections Above STh/L=  0.600          8 Note
PLAT913_ALERT_3_G Missing # of Very Strong Reflections in FCF ....          2 Note
PLAT933_ALERT_2_G Number of HKL-OMIT Records in Embedded .res File         18 Note
PLAT978_ALERT_2_G Number C-C Bonds with Positive Residual Density.          0 Info
```

---

1 **ALERT level A** = Most likely a serious problem - resolve or explain
8 **ALERT level B** = A potentially serious problem, consider carefully
39 **ALERT level C** = Check. Ensure it is not caused by an omission or oversight
110 **ALERT level G** = General information/check it is not something unexpected

4 ALERT type 1 CIF construction/syntax error, inconsistent or missing data
57 ALERT type 2 Indicator that the structure model may be wrong or deficient
29 ALERT type 3 Indicator that the structure quality may be low
61 ALERT type 4 Improvement, methodology, query or suggestion
7 ALERT type 5 Informative message, check

It is advisable to attempt to resolve as many as possible of the alerts in all categories. Often the minor alerts point to easily fixed oversights, errors and omissions in your CIF or refinement strategy, so attention to these fine details can be worthwhile. In order to resolve some of the more serious problems it may be necessary to carry out additional measurements or structure refinements. However, the purpose of your study may justify the reported deviations and the more serious of these should normally be commented upon in the discussion or experimental section of a paper or in the "special_details" fields of the CIF. checkCIF was carefully designed to identify outliers and unusual parameters, but every test has its limitations and alerts that are not important in a particular case may appear. Conversely, the absence of alerts does not guarantee there are no aspects of the results needing attention. It is up to the individual to critically assess their own results and, if necessary, seek expert advice.

Publication of your CIF in IUCr journals

A basic structural check has been run on your CIF. These basic checks will be run on all CIFs submitted for publication in IUCr journals (*Acta Crystallographica*, *Journal of Applied Crystallography*, *Journal of Synchrotron Radiation*); however, if you intend to submit to *Acta Crystallographica Section C* or *E* or *IUCrData*, you should make sure that full publication checks are run on the final version of your CIF prior to submission.

Publication of your CIF in other journals

Please refer to the *Notes for Authors* of the relevant journal for any special instructions relating to CIF submission.

PLATON version of 28/11/2022; check.def file version of 28/11/2022

Datablock HMF-MeCN@MMF - ellipsoid plot

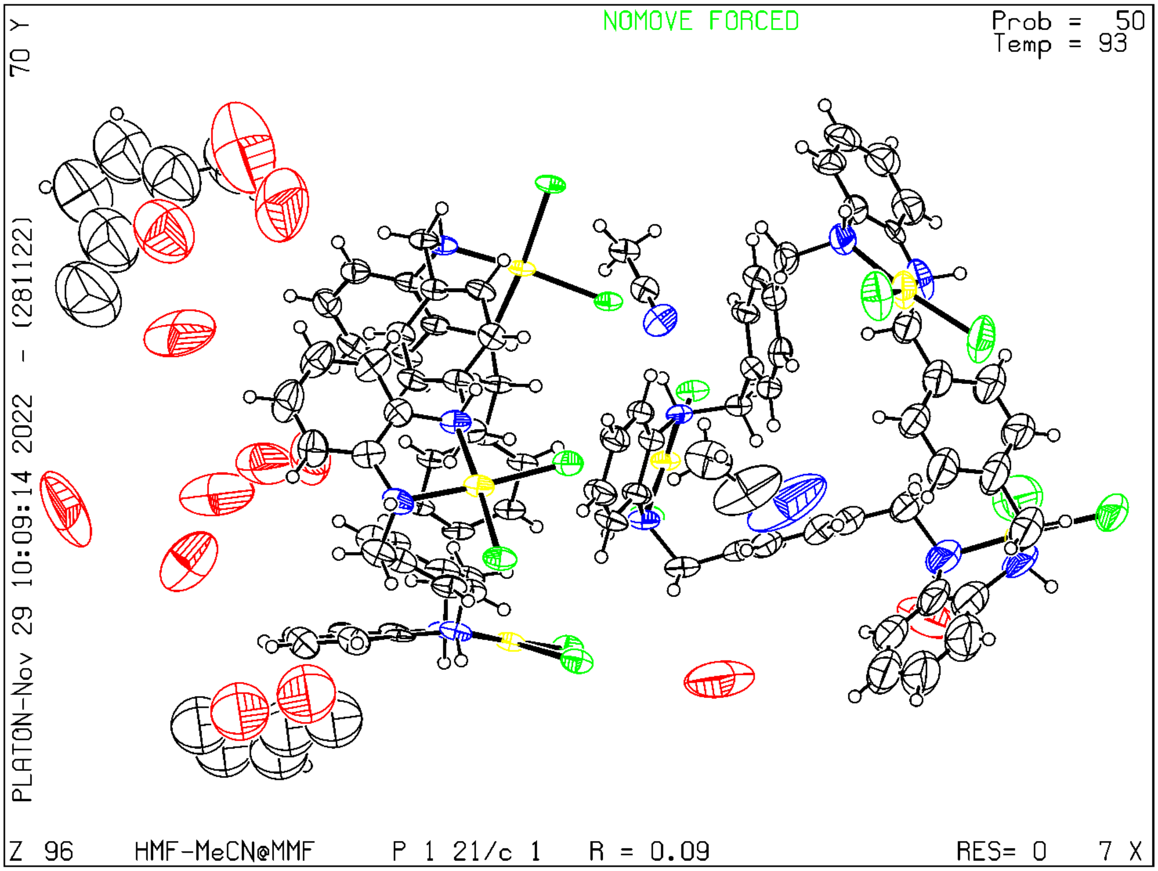

Supplement: Supplementary file 2 — Supporting Information [file SMLL-20-2405507-s001.zip › HMF-MeCN@MMF_checkcif.pdf]
