# Supplementary material for: Porous Supramolecular Crystalline Probe that Detects Non‐Covalent Interactions Involved in Molecular Recognition of Furanic Compounds
Source: Small. 2024 Jul 30;20(49):2405507. doi: 10.1002/smll.202405507 (PMC11618713; doi:10.1002/smll.202405507)

checkCIF/PLATON report

Structure factors have been supplied for datablock(s) HMF-water@MMF

THIS REPORT IS FOR GUIDANCE ONLY. IF USED AS PART OF A REVIEW PROCEDURE FOR PUBLICATION, IT SHOULD NOT REPLACE THE EXPERTISE OF AN EXPERIENCED CRYSTALLOGRAPHIC REFEREE.

No syntax errors found. CIF dictionary Interpreting this report

Datablock: HMF-water@MMF

Bond precision: C-C = 0.0080 A Wavelength=1.54184

Cell:	a=19.65460(19)	b=52.6684(5)	c=14.16700(11)
	alpha=90	beta=91.4550(8)	gamma=90
Temperature:	93 K		

	Calculated	Reported
Volume	14660.6(2)	14660.6(2)
Space group	P 21/c	P 1 21/c 1
Hall group	-P 2ybc	-P 2ybc
Moiety formula	C42 H42 Cl6 N6 Pd3, 0.357(C6 H5 O3), 0.142(C5 H2 O2), 3.75(O)	2(C42 H42 Cl6 N6 Pd3), 0.715(C6 H5 O3), 7.5(O), 0.285(C5 H2 O2)
Sum formula	C44.86 H44.07 Cl6 N6 O5.11 Pd3	C89.71 H88.14 Cl12 N12 O10.22 Pd6
Mr	1280.85	2561.68
Dx,g cm-3	1.161	1.161
Z	8	4
Mu (mm-1)	8.168	8.168
F000	5088.6	5089.0
F000'	5120.20	
h,k,lmax	23,63,17	23,63,17
Nref	26839	26750
Tmin,Tmax	0.297,0.618	0.503,1.000
Tmin'	0.175	

Correction method= # Reported T Limits: Tmin=0.503 Tmax=1.000
AbsCorr = MULTI-SCAN

Data completeness= 0.997 Theta(max)= 68.250

R(reflections)= 0.0764(21783) wR2(reflections)= 0.2380(26750)

S = 1.049 Npar= 1145

The following ALERTS were generated. Each ALERT has the format
test-name_ALERT_alert-type_alert-level.
Click on the hyperlinks for more details of the test.

Alert level A

PLAT602_ALERT_2_A Solvent Accessible VOID(S) in Structure .... ! Check

Author Response: Some solvents in the large pore could not be located due to severe disordering.

Alert level B

PLAT306_ALERT_2_B Isolated Oxygen Atom (H-atoms Missing ?) .... O1W Check

Author Response: Hydrogen atoms of water molecules and OH groups of the guests could not be located in the difference electron density maps.

PLAT306_ALERT_2_B Isolated Oxygen Atom (H-atoms Missing ?) .... O2W Check

Author Response: Hydrogen atoms of water molecules and OH groups of the guests could not be located in the difference electron density maps.

PLAT306_ALERT_2_B Isolated Oxygen Atom (H-atoms Missing ?) .... O3W Check

Author Response: Hydrogen atoms of water molecules and OH groups of the guests could not be located in the difference electron density maps.

PLAT430_ALERT_2_B Short Inter D...A Contact O1W ..O2W . 2.71 Ang.
x,y,z = 1_555 Check

Author Response: The short contacts come from H2O...H2O, though hydrogen atoms of water molecules could not be located in the difference electron density maps. Therefore, these contacts can be assigned to hydrogen bonding between water molecules.

PLAT430_ALERT_2_B Short Inter D...A Contact O3W ..O4W . 2.72 Ang.
x,y,z = 1_555 Check

Author Response: The short contacts come from H2O...H2O, though hydrogen atoms of water molecules could not be located in the difference electron density maps. Therefore, these contacts can be assigned to hydrogen bonding between water molecules.

```
PLAT430_ALERT_2_B Short Inter D...A Contact  O5W       ..O6W      .       2.81 Ang.
                                                      x,y,z  =      1_555 Check
```

Author Response: The short contacts come from H2O...H2O, though hydrogen atoms of water molecules could not be located in the difference electron density maps. Therefore, these contacts can be assigned to hydrogen bonding between water molecules.

```
PLAT971_ALERT_2_B Check Calcd Resid. Dens.  0.73Ang From C62            2.96 eA-3
```

Author Response: The atom type is correct and there is no evidence of twinning.

```
PLAT971_ALERT_2_B Check Calcd Resid. Dens.  0.44Ang From C20            2.70 eA-3
```

Author Response: The atom type is correct and there is no evidence of twinning.

```
PLAT971_ALERT_2_B Check Calcd Resid. Dens.  1.04Ang From N11            2.53 eA-3
```

Author Response: The atom type is correct and there is no evidence of twinning.

```
PLAT972_ALERT_2_B Check Calcd Resid. Dens.  0.74Ang From Pd6           -2.76 eA-3
```

Author Response: The atom type is correct and there is no evidence of twinning. The large residual density on Pd atoms may be Due to an anomalous dispersion effect and has no chemical significance.

```
PLAT973_ALERT_2_B Check Calcd Positive Resid. Density on       Pd1       1.61 eA-3
```

Author Response: The atom type is correct and there is no evidence of twinning. The large residual density on Pd atoms may be Due to an anomalous dispersion effect and has no chemical significance.

Alert level C

```
PLAT041_ALERT_1_C Calc. and Reported SumFormula    Strings  Differ     Please Check
PLAT042_ALERT_1_C Calc. and Reported MoietyFormula Strings  Differ     Please Check
PLAT213_ALERT_2_C Atom N11             has ADP max/min Ratio .....        3.6 prolat
PLAT220_ALERT_2_C NonSolvent   Resd 2  C   Ueq(max)/Ueq(min) Range        3.7 Ratio
PLAT220_ALERT_2_C NonSolvent   Resd 2  N   Ueq(max)/Ueq(min) Range        3.1 Ratio
```

```
PLAT234_ALERT_4_C Large Hirshfeld Difference C17      --C18      .       0.16 Ang.
PLAT241_ALERT_2_C High   'MainMol' Ueq as Compared to Neighbors of        C18 Check
PLAT241_ALERT_2_C High   'MainMol' Ueq as Compared to Neighbors of        C73 Check
PLAT242_ALERT_2_C Low    'MainMol' Ueq as Compared to Neighbors of        C20 Check
PLAT242_ALERT_2_C Low    'MainMol' Ueq as Compared to Neighbors of        Pd6 Check
PLAT250_ALERT_2_C Large U3/U1 Ratio for Average U(i,j) Tensor ....        2.1 Note
PLAT250_ALERT_2_C Large U3/U1 Ratio for Average U(i,j) Tensor ....        2.2 Note
PLAT260_ALERT_2_C Large Average Ueq of Residue Including       O1A      0.120 Check
PLAT260_ALERT_2_C Large Average Ueq of Residue Including       O1B      0.111 Check
PLAT260_ALERT_2_C Large Average Ueq of Residue Including       O3W      0.157 Check
PLAT260_ALERT_2_C Large Average Ueq of Residue Including       O5W      0.251 Check
PLAT260_ALERT_2_C Large Average Ueq of Residue Including       O8W      0.223 Check
PLAT260_ALERT_2_C Large Average Ueq of Residue Including       O9W      0.255 Check
PLAT260_ALERT_2_C Large Average Ueq of Residue Including      O13W      0.169 Check
PLAT260_ALERT_2_C Large Average Ueq of Residue Including       O14      0.279 Check
PLAT260_ALERT_2_C Large Average Ueq of Residue Including       O4W      0.249 Check
PLAT260_ALERT_2_C Large Average Ueq of Residue Including       O6W      0.140 Check
PLAT260_ALERT_2_C Large Average Ueq of Residue Including       O7W      0.157 Check
PLAT260_ALERT_2_C Large Average Ueq of Residue Including      O10W      0.233 Check
PLAT260_ALERT_2_C Large Average Ueq of Residue Including      O11W      0.197 Check
PLAT260_ALERT_2_C Large Average Ueq of Residue Including      O12W      0.174 Check
PLAT309_ALERT_2_C Single Bonded Oxygen (C-O > 1.3 Ang) ..........         O2B Check
PLAT411_ALERT_2_C Short Inter H...H Contact  H58      ..H84A     .       2.06 Ang.
                                                   x,y,1+z  =      1_556 Check
PLAT420_ALERT_2_C D-H Bond Without Acceptor  N7       --H7       .    Please Check
PLAT767_ALERT_4_C INS Embedded LIST 6 Instruction Should be LIST 4     Please Check
PLAT906_ALERT_3_C Large K Value in the Analysis of Variance ......      4.505 Check
PLAT911_ALERT_3_C Missing FCF Refl Between Thmin & STh/L=    0.600         68 Report
PLAT918_ALERT_3_C Reflection(s) with I(obs) much Smaller I(calc) .          7 Check
PLAT971_ALERT_2_C Check Calcd Resid. Dens.  1.03Ang From Pd6           2.28 eA-3
```

Author Response: The atom type is correct and there is no evidence of twinning.

```
PLAT971_ALERT_2_C Check Calcd Resid. Dens.  0.56Ang From C34            1.75 eA-3
```

Author Response: The atom type is correct and there is no evidence of twinning.

```
PLAT972_ALERT_2_C Check Calcd Resid. Dens.  0.53Ang From Pd6           -2.12 eA-3
```

Author Response: The atom type is correct and there is no evidence of twinning. The large residual density on Pd atoms may be Due to an anomalous dispersion effect and has no chemical significance.

```
PLAT972_ALERT_2_C Check Calcd Resid. Dens.  0.33Ang From Pd6           -1.83 eA-3
```

Author Response: The atom type is correct and there is no evidence of twinning. The large residual density on Pd atoms may be Due to an anomalous dispersion effect and has no chemical significance.

```
PLAT972_ALERT_2_C Check Calcd Resid. Dens.  0.47Ang From Pd6           -1.73 eA-3
```

Author Response: The atom type is correct and there is no evidence of twinning. The large residual density on Pd atoms may be Due to an anomalous dispersion effect and has no chemical significance.

```
PLAT972_ALERT_2_C Check Calcd Resid. Dens.  0.78Ang From Pd5           -1.69 eA-3
```

Author Response: The atom type is correct and there is no evidence of twinning. The large residual density on Pd atoms may be Due to an anomalous dispersion effect and has no chemical significance.

```
PLAT975_ALERT_2_C Check Calcd Resid. Dens.  0.99Ang From O8W     .      0.93 eA-3
```

Alert level G

```
CELLZ01_ALERT_1_G Difference between formula and atom_site contents detected.
CELLZ01_ALERT_1_G ALERT: check formula stoichiometry or atom site occupancies.
           From the CIF: _cell_formula_units_Z    4
           From the CIF: _chemical_formula_sum  C89.71 H88.14 Cl12 N12 O10.22 Pd6
           TEST: Compare cell contents of formula and atom_site data

           atom    Z*formula  cif sites diff
           C        358.84    358.86   -0.02
           H        352.56    352.58   -0.02
           Cl        48.00     48.00    0.00
           N         48.00     48.00    0.00
           O         40.88     40.86    0.02
           Pd        24.00     24.00    0.00
PLAT002_ALERT_2_G Number of Distance or Angle Restraints on AtSite          9 Note
PLAT003_ALERT_2_G Number of Uiso or Uij Restrained non-H Atoms ...         25 Report
PLAT007_ALERT_5_G Number of Unrefined Donor-H Atoms .............         12 Report
PLAT045_ALERT_1_G Calculated and Reported Z Differ by a Factor ...          2 Check
PLAT068_ALERT_1_G Reported F000 Differs from Calcd (or Missing)...     Please Check
PLAT072_ALERT_2_G SHELXL First  Parameter in WGHT  Unusually Large       0.14 Report
PLAT083_ALERT_2_G SHELXL Second Parameter in WGHT  Unusually Large      85.32 Why ?
PLAT142_ALERT_4_G s.u. on b - Axis Small or Missing .............    0.00050 Ang.
PLAT143_ALERT_4_G s.u. on c - Axis Small or Missing .............    0.00011 Ang.
PLAT172_ALERT_4_G The CIF-Embedded .res File Contains DFIX Records          8 Report
PLAT174_ALERT_4_G The CIF-Embedded .res File Contains FLAT Records          1 Report
PLAT178_ALERT_4_G The CIF-Embedded .res File Contains SIMU Records          4 Report
PLAT180_ALERT_4_G Check Cell Rounding: # of Values Ending with 0 =          3 Note
PLAT186_ALERT_4_G The CIF-Embedded .res File Contains ISOR Records         13 Report
PLAT187_ALERT_4_G The CIF-Embedded .res File Contains RIGU Records         10 Report
PLAT188_ALERT_3_G A non-default SIMU restraint value has been used     0.0100 Report
PLAT190_ALERT_3_G A non-default RIGU restraint value for First Par     0.0020 Report
PLAT190_ALERT_3_G A non-default RIGU restraint value for SecondPar     0.0020 Report
PLAT190_ALERT_3_G A non-default RIGU restraint value for First Par     0.0010 Report
PLAT190_ALERT_3_G A non-default RIGU restraint value for SecondPar     0.0010 Report
PLAT190_ALERT_3_G A non-default RIGU restraint value for First Par     0.0020 Report
PLAT190_ALERT_3_G A non-default RIGU restraint value for SecondPar     0.0020 Report
PLAT190_ALERT_3_G A non-default RIGU restraint value for First Par     0.0020 Report
PLAT190_ALERT_3_G A non-default RIGU restraint value for SecondPar     0.0020 Report
```

```
PLAT190_ALERT_3_G A non-default RIGU restraint value for First Par     0.0020 Report
PLAT190_ALERT_3_G A non-default RIGU restraint value for SecondPar     0.0020 Report
PLAT190_ALERT_3_G A non-default RIGU restraint value for First Par     0.0020 Report
PLAT190_ALERT_3_G A non-default RIGU restraint value for SecondPar     0.0020 Report
PLAT190_ALERT_3_G A non-default RIGU restraint value for First Par     0.0020 Report
PLAT190_ALERT_3_G A non-default RIGU restraint value for SecondPar     0.0020 Report
PLAT232_ALERT_2_G Hirshfeld Test Diff (M-X)  Pd6      --N11      .        9.9 s.u.
PLAT300_ALERT_4_G Atom Site Occupancy of O5W       Constrained at        0.5 Check
PLAT300_ALERT_4_G Atom Site Occupancy of O8W       Constrained at        0.5 Check
PLAT300_ALERT_4_G Atom Site Occupancy of O9W       Constrained at        0.5 Check
PLAT300_ALERT_4_G Atom Site Occupancy of O14       Constrained at        0.5 Check
PLAT300_ALERT_4_G Atom Site Occupancy of O4W       Constrained at     0.3333 Check
PLAT300_ALERT_4_G Atom Site Occupancy of O6W       Constrained at       0.25 Check
PLAT300_ALERT_4_G Atom Site Occupancy of O7W       Constrained at     0.3333 Check
PLAT300_ALERT_4_G Atom Site Occupancy of O10W      Constrained at       0.25 Check
PLAT300_ALERT_4_G Atom Site Occupancy of O11W      Constrained at     0.3333 Check
PLAT302_ALERT_4_G Anion/Solvent/Minor-Residue Disorder (Resd  3  )       100% Note
PLAT302_ALERT_4_G Anion/Solvent/Minor-Residue Disorder (Resd  4  )       100% Note
PLAT302_ALERT_4_G Anion/Solvent/Minor-Residue Disorder (Resd  8  )       100% Note
PLAT302_ALERT_4_G Anion/Solvent/Minor-Residue Disorder (Resd  9  )       100% Note
PLAT302_ALERT_4_G Anion/Solvent/Minor-Residue Disorder (Resd 10  )       100% Note
PLAT302_ALERT_4_G Anion/Solvent/Minor-Residue Disorder (Resd 11  )       100% Note
PLAT302_ALERT_4_G Anion/Solvent/Minor-Residue Disorder (Resd 12  )       100% Note
PLAT302_ALERT_4_G Anion/Solvent/Minor-Residue Disorder (Resd 13  )       100% Note
PLAT302_ALERT_4_G Anion/Solvent/Minor-Residue Disorder (Resd 14  )       100% Note
PLAT302_ALERT_4_G Anion/Solvent/Minor-Residue Disorder (Resd 15  )       100% Note
PLAT302_ALERT_4_G Anion/Solvent/Minor-Residue Disorder (Resd 16  )       100% Note
PLAT302_ALERT_4_G Anion/Solvent/Minor-Residue Disorder (Resd 17  )       100% Note
PLAT302_ALERT_4_G Anion/Solvent/Minor-Residue Disorder (Resd 18  )       100% Note
PLAT304_ALERT_4_G Non-Integer Number of Atoms in ..... (Resd  3  )      10.01 Check
PLAT304_ALERT_4_G Non-Integer Number of Atoms in ..... (Resd  4  )       2.57 Check
PLAT304_ALERT_4_G Non-Integer Number of Atoms in ..... (Resd  8  )       0.50 Check
PLAT304_ALERT_4_G Non-Integer Number of Atoms in ..... (Resd  9  )       0.50 Check
PLAT304_ALERT_4_G Non-Integer Number of Atoms in ..... (Resd 10  )       0.50 Check
PLAT304_ALERT_4_G Non-Integer Number of Atoms in ..... (Resd 11  )       0.71 Check
PLAT304_ALERT_4_G Non-Integer Number of Atoms in ..... (Resd 12  )       0.50 Check
PLAT304_ALERT_4_G Non-Integer Number of Atoms in ..... (Resd 13  )       0.33 Check
PLAT304_ALERT_4_G Non-Integer Number of Atoms in ..... (Resd 14  )       0.25 Check
PLAT304_ALERT_4_G Non-Integer Number of Atoms in ..... (Resd 15  )       0.33 Check
PLAT304_ALERT_4_G Non-Integer Number of Atoms in ..... (Resd 16  )       0.25 Check
PLAT304_ALERT_4_G Non-Integer Number of Atoms in ..... (Resd 17  )       0.33 Check
PLAT304_ALERT_4_G Non-Integer Number of Atoms in ..... (Resd 18  )       0.28 Check
PLAT311_ALERT_2_G Isolated Disordered Oxygen Atom (No H's ?) .....        O5W Check
PLAT311_ALERT_2_G Isolated Disordered Oxygen Atom (No H's ?) .....        O8W Check
PLAT311_ALERT_2_G Isolated Disordered Oxygen Atom (No H's ?) .....        O9W Check
PLAT311_ALERT_2_G Isolated Disordered Oxygen Atom (No H's ?) .....       O13W Check
PLAT311_ALERT_2_G Isolated Disordered Oxygen Atom (No H's ?) .....        O14 Check
PLAT311_ALERT_2_G Isolated Disordered Oxygen Atom (No H's ?) .....        O4W Check
PLAT311_ALERT_2_G Isolated Disordered Oxygen Atom (No H's ?) .....        O6W Check
PLAT311_ALERT_2_G Isolated Disordered Oxygen Atom (No H's ?) .....        O7W Check
PLAT311_ALERT_2_G Isolated Disordered Oxygen Atom (No H's ?) .....       O10W Check
PLAT311_ALERT_2_G Isolated Disordered Oxygen Atom (No H's ?) .....       O11W Check
PLAT311_ALERT_2_G Isolated Disordered Oxygen Atom (No H's ?) .....       O12W Check
PLAT398_ALERT_2_G Deviating  C-O-C   Angle From 120 for O1A      .      105.0 Degree
PLAT398_ALERT_2_G Deviating  C-O-C   Angle From 120 for O1B      .      108.0 Degree
PLAT720_ALERT_4_G Number of Unusual/Non-Standard Labels ..........          4 Note
PLAT790_ALERT_4_G Centre of Gravity not Within Unit Cell: Resd.  #         11 Note
```

```
              O
PLAT790_ALERT_4_G Centre of Gravity not Within Unit Cell: Resd.  #         12 Note
              O
PLAT793_ALERT_4_G Model has Chirality at N1          (Centro SPGR)          S Verify
PLAT793_ALERT_4_G Model has Chirality at N2          (Centro SPGR)          R Verify
PLAT793_ALERT_4_G Model has Chirality at N3          (Centro SPGR)          S Verify
PLAT793_ALERT_4_G Model has Chirality at N4          (Centro SPGR)          R Verify
PLAT793_ALERT_4_G Model has Chirality at N5          (Centro SPGR)          S Verify
PLAT793_ALERT_4_G Model has Chirality at N6          (Centro SPGR)          R Verify
PLAT793_ALERT_4_G Model has Chirality at N7          (Centro SPGR)          R Verify
PLAT793_ALERT_4_G Model has Chirality at N8          (Centro SPGR)          S Verify
PLAT793_ALERT_4_G Model has Chirality at N9          (Centro SPGR)          S Verify
PLAT793_ALERT_4_G Model has Chirality at N10         (Centro SPGR)          R Verify
PLAT793_ALERT_4_G Model has Chirality at N11         (Centro SPGR)          R Verify
PLAT793_ALERT_4_G Model has Chirality at N12         (Centro SPGR)          S Verify
PLAT794_ALERT_5_G Tentative Bond Valency for Pd1       (II)      .       2.03 Info
PLAT794_ALERT_5_G Tentative Bond Valency for Pd2       (II)      .       2.08 Info
PLAT794_ALERT_5_G Tentative Bond Valency for Pd3       (II)      .       2.07 Info
PLAT794_ALERT_5_G Tentative Bond Valency for Pd4       (II)      .       2.03 Info
PLAT794_ALERT_5_G Tentative Bond Valency for Pd5       (II)      .       2.11 Info
PLAT794_ALERT_5_G Tentative Bond Valency for Pd6       (II)      .       2.36 Info
PLAT860_ALERT_3_G Number of Least-Squares Restraints ............         441 Note
PLAT883_ALERT_1_G No Info/Value for _atom_sites_solution_primary .     Please Do !
PLAT910_ALERT_3_G Missing # of FCF Reflection(s) Below Theta(Min).          4 Note
PLAT912_ALERT_4_G Missing # of FCF Reflections Above STh/L=  0.600         17 Note
PLAT913_ALERT_3_G Missing # of Very Strong Reflections in FCF ....          2 Note
PLAT933_ALERT_2_G Number of HKL-OMIT Records in Embedded .res File         21 Note
PLAT941_ALERT_3_G Average HKL Measurement Multiplicity ..........         2.9 Low
PLAT978_ALERT_2_G Number C-C Bonds with Positive Residual Density.          0 Info
```

---

1 **ALERT level A** = Most likely a serious problem - resolve or explain
11 **ALERT level B** = A potentially serious problem, consider carefully
40 **ALERT level C** = Check. Ensure it is not caused by an omission or oversight
110 **ALERT level G** = General information/check it is not something unexpected

7 ALERT type 1 CIF construction/syntax error, inconsistent or missing data
65 ALERT type 2 Indicator that the structure model may be wrong or deficient
22 ALERT type 3 Indicator that the structure quality may be low
61 ALERT type 4 Improvement, methodology, query or suggestion
7 ALERT type 5 Informative message, check

It is advisable to attempt to resolve as many as possible of the alerts in all categories. Often the minor alerts point to easily fixed oversights, errors and omissions in your CIF or refinement strategy, so attention to these fine details can be worthwhile. In order to resolve some of the more serious problems it may be necessary to carry out additional measurements or structure refinements. However, the purpose of your study may justify the reported deviations and the more serious of these should normally be commented upon in the discussion or experimental section of a paper or in the "special_details" fields of the CIF. checkCIF was carefully designed to identify outliers and unusual parameters, but every test has its limitations and alerts that are not important in a particular case may appear. Conversely, the absence of alerts does not guarantee there are no aspects of the results needing attention. It is up to the individual to critically assess their own results and, if necessary, seek expert advice.

Publication of your CIF in IUCr journals

A basic structural check has been run on your CIF. These basic checks will be run on all CIFs submitted for publication in IUCr journals (*Acta Crystallographica*, *Journal of Applied Crystallography*, *Journal of Synchrotron Radiation*); however, if you intend to submit to *Acta Crystallographica Section C* or *E* or *IUCrData*, you should make sure that full publication checks are run on the final version of your CIF prior to submission.

Publication of your CIF in other journals

Please refer to the *Notes for Authors* of the relevant journal for any special instructions relating to CIF submission.

PLATON version of 18/11/2022; check.def file version of 18/11/2022

Datablock HMF-water@MMF - ellipsoid plot

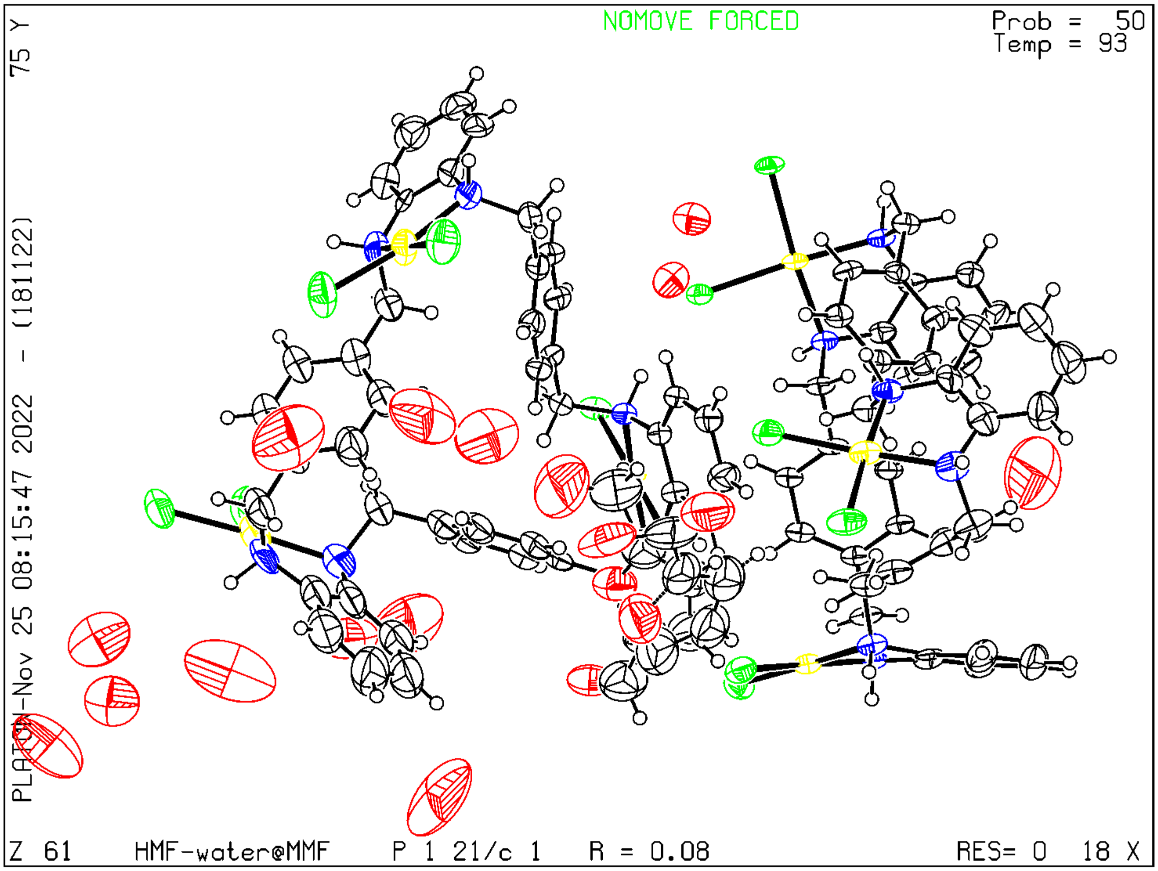

Supplement: Supplementary file 2 — Supporting Information [file SMLL-20-2405507-s001.zip › HMF-water@MMF_checkcif.pdf]
