# Supplementary material for: Porous Supramolecular Crystalline Probe that Detects Non‐Covalent Interactions Involved in Molecular Recognition of Furanic Compounds
Source: Small. 2024 Jul 30;20(49):2405507. doi: 10.1002/smll.202405507 (PMC11618713; doi:10.1002/smll.202405507)

checkCIF/PLATON report

Structure factors have been supplied for datablock(s) Acfuran-MeCN@MMF

THIS REPORT IS FOR GUIDANCE ONLY. IF USED AS PART OF A REVIEW PROCEDURE FOR PUBLICATION, IT SHOULD NOT REPLACE THE EXPERTISE OF AN EXPERIENCED CRYSTALLOGRAPHIC REFEREE.

No syntax errors found. CIF dictionary Interpreting this report

Datablock: Acfuran-MeCN@MMF

Bond precision: C-C = 0.0126 A Wavelength=1.54184

Cell:	a=19.57780(15)	b=52.1918(5)	c=14.27570(8)
	alpha=90	beta=90.5101(6)	gamma=90
Temperature:	93 K		

	Calculated	Reported
Volume	14586.3(2)	14586.3(2)
Space group	P 21/c	P 1 21/c 1
Hall group	-P 2ybc	-P 2ybc
Moiety formula	C42 H42 Cl6 N6 Pd3, 0.315(C6 H6 O2), 1.768(C2 H3 N), 2.227(O)	2(C42 H42 Cl6 N6 Pd3), 3.535(C2 H3 N), 4.452(O), 0.632(C6 H6 O2
Sum formula	C47.43 H49.19 Cl6 N7.77 O2.86 Pd3	C94.86 H98.39 Cl12 N15.53 O5.71 Pd6
Mr	1305.63	2611.33
Dx,g cm-3	1.189	1.189
Z	8	4
Mu (mm-1)	8.202	8.202
F000	5207.9	5208.0
F000'	5239.26	
h,k,lmax	23,62,17	23,62,17
Nref	26719	26651
Tmin,Tmax	0.200,0.409	0.332,1.000
Tmin'	0.101	

Correction method= # Reported T Limits: Tmin=0.332 Tmax=1.000
AbsCorr = MULTI-SCAN

Data completeness= 0.997 Theta(max)= 68.248

R(reflections)= 0.1166(22933) wR2(reflections)= 0.3135(26651)

S = 1.095 Npar= 1243

The following ALERTS were generated. Each ALERT has the format
test-name_ALERT_alert-type_alert-level.
Click on the hyperlinks for more details of the test.

Alert level A

PLAT602_ALERT_2_A Solvent Accessible VOID(S) in Structure .... ! Check

Author Response: Some solvents in the large pore could not be located due to severe disordering.

PLAT971_ALERT_2_A Check Calcd Resid. Dens. 1.10Ang From Pd1 4.50 eA-3

Author Response: The atom type is correct and there is no evidence of twinning.

PLAT971_ALERT_2_A Check Calcd Resid. Dens. 0.95Ang From Pd2 3.66 eA-3

Author Response: The atom type is correct and there is no evidence of twinning.

Alert level B

PLAT306_ALERT_2_B Isolated Oxygen Atom (H-atoms Missing ?) .... O3W Check

Author Response: Hydrogen atoms of water molecules could not be located in the difference electron density maps.

PLAT971_ALERT_2_B Check Calcd Resid. Dens. 1.10Ang From Pd4 3.46 eA-3

Author Response: The atom type is correct and there is no evidence of twinning.

PLAT971_ALERT_2_B Check Calcd Resid. Dens. 1.01Ang From N1 3.44 eA-3

Author Response: The atom type is correct and there is no evidence of twinning.

PLAT971_ALERT_2_B Check Calcd Resid. Dens. 1.14Ang From Pd3 3.25 eA-3

Author Response: The atom type is correct and there is no evidence of twinning.

PLAT971_ALERT_2_B Check Calcd Resid. Dens. 1.15Ang From Pd3   3.15 eA-3

Author Response: The atom type is correct and there is no evidence of twinning.

PLAT971_ALERT_2_B Check Calcd Resid. Dens. 0.65Ang From C20   2.80 eA-3

Author Response: The atom type is correct and there is no evidence of twinning.

PLAT971_ALERT_2_B Check Calcd Resid. Dens. 1.16Ang From Pd4   2.80 eA-3

Author Response: The atom type is correct and there is no evidence of twinning.

PLAT972_ALERT_2_B Check Calcd Resid. Dens. 0.80Ang From Pd6   -2.54 eA-3

Author Response: The atom type is correct and there is no evidence of twinning. The large residual density on Pd atoms may be Due to an anomalous dispersion effect and has no chemical significance.

Alert level C

```
DIFMX02_ALERT_1_C  The maximum difference density is > 0.1*ZMAX*0.75
            The relevant atom site should be identified.
PLAT041_ALERT_1_C Calc. and Reported SumFormula    Strings  Differ     Please Check
PLAT042_ALERT_1_C Calc. and Reported MoietyFormula Strings  Differ     Please Check
PLAT082_ALERT_2_C High R1 Value ..................................       0.12 Report
PLAT084_ALERT_3_C High wR2 Value (i.e. > 0.25) ...................       0.31 Report
PLAT097_ALERT_2_C Large Reported Max.  (Positive) Residual Density       4.35 eA-3
PLAT213_ALERT_2_C Atom N3              has ADP max/min Ratio .....        3.4 prolat
PLAT220_ALERT_2_C NonSolvent   Resd 2  C   Ueq(max)/Ueq(min) Range        3.7 Ratio
PLAT220_ALERT_2_C NonSolvent   Resd 2 Cl   Ueq(max)/Ueq(min) Range        3.5 Ratio
PLAT220_ALERT_2_C NonSolvent   Resd 2  N   Ueq(max)/Ueq(min) Range        3.1 Ratio
PLAT241_ALERT_2_C High   'MainMol' Ueq as Compared to Neighbors of        C70 Check
PLAT242_ALERT_2_C Low    'MainMol' Ueq as Compared to Neighbors of        Pd6 Check
PLAT242_ALERT_2_C Low    'MainMol' Ueq as Compared to Neighbors of        C62 Check
PLAT250_ALERT_2_C Large U3/U1 Ratio for Average U(i,j) Tensor ....        2.6 Note
PLAT250_ALERT_2_C Large U3/U1 Ratio for Average U(i,j) Tensor ....        2.1 Note
PLAT250_ALERT_2_C Large U3/U1 Ratio for Average U(i,j) Tensor ....        3.9 Note
PLAT260_ALERT_2_C Large Average Ueq of Residue Including       O1A      0.216 Check
PLAT260_ALERT_2_C Large Average Ueq of Residue Including       N7S      0.177 Check
PLAT260_ALERT_2_C Large Average Ueq of Residue Including       N2S      0.108 Check
PLAT260_ALERT_2_C Large Average Ueq of Residue Including       N5S      0.104 Check
PLAT260_ALERT_2_C Large Average Ueq of Residue Including       N6S      0.130 Check
PLAT260_ALERT_2_C Large Average Ueq of Residue Including       O3W      0.166 Check
PLAT260_ALERT_2_C Large Average Ueq of Residue Including       O4W      0.250 Check
PLAT260_ALERT_2_C Large Average Ueq of Residue Including       O5W      0.230 Check
PLAT260_ALERT_2_C Large Average Ueq of Residue Including       O6W      0.273 Check
PLAT260_ALERT_2_C Large Average Ueq of Residue Including       O1W      0.146 Check
PLAT260_ALERT_2_C Large Average Ueq of Residue Including       O2W      0.203 Check
PLAT260_ALERT_2_C Large Average Ueq of Residue Including       O7W      0.248 Check
PLAT260_ALERT_2_C Large Average Ueq of Residue Including       O8W      0.156 Check
```

```
PLAT260_ALERT_2_C Large Average Ueq of Residue Including       O9W      0.156 Check
PLAT260_ALERT_2_C Large Average Ueq of Residue Including      O10W      0.153 Check
PLAT260_ALERT_2_C Large Average Ueq of Residue Including      O11W      0.150 Check
PLAT342_ALERT_3_C Low Bond Precision on  C-C Bonds ..............    0.01256 Ang.
PLAT411_ALERT_2_C Short Inter H...H Contact  H58       ..H84A     .       2.11 Ang.
                                                   x,y,-1+z  =      1_554 Check
PLAT420_ALERT_2_C D-H Bond Without Acceptor  N7       --H7       .     Please Check
PLAT767_ALERT_4_C INS Embedded LIST 6 Instruction Should be LIST 4     Please Check
PLAT906_ALERT_3_C Large K Value in the Analysis of Variance ......     12.638 Check
PLAT906_ALERT_3_C Large K Value in the Analysis of Variance ......      3.039 Check
PLAT911_ALERT_3_C Missing FCF Refl Between Thmin & STh/L=    0.600         53 Report
PLAT918_ALERT_3_C Reflection(s) with I(obs) much Smaller I(calc) .          4 Check
PLAT971_ALERT_2_C Check Calcd Resid. Dens.  1.11Ang From Pd6            2.35 eA-3
```

Author Response: The atom type is correct and there is no evidence of twinning.

```
PLAT971_ALERT_2_C Check Calcd Resid. Dens.  1.07Ang From N11            2.21 eA-3
```

Author Response: The atom type is correct and there is no evidence of twinning.

```
PLAT971_ALERT_2_C Check Calcd Resid. Dens.  1.12Ang From Pd2            2.08 eA-3
```

Author Response: The atom type is correct and there is no evidence of twinning.

```
PLAT971_ALERT_2_C Check Calcd Resid. Dens.  0.49Ang From Pd1            1.98 eA-3
```

Author Response: The atom type is correct and there is no evidence of twinning.

```
PLAT971_ALERT_2_C Check Calcd Resid. Dens.  1.04Ang From N9             1.90 eA-3
```

Author Response: The atom type is correct and there is no evidence of twinning.

```
PLAT971_ALERT_2_C Check Calcd Resid. Dens.  1.08Ang From Pd5            1.83 eA-3
```

Author Response: The atom type is correct and there is no evidence of twinning.

```
PLAT971_ALERT_2_C Check Calcd Resid. Dens.  0.71Ang From C62            1.77 eA-3
```

Author Response: The atom type is correct and there is no evidence of twinning.

```
PLAT971_ALERT_2_C Check Calcd Resid. Dens.  1.03Ang From N10            1.76 eA-3
```

Author Response: The atom type is correct and there is no evidence of twinning.

PLAT971_ALERT_2_C Check Calcd Resid. Dens. 0.23Ang From Pd1   1.64 eA-3

Author Response: The atom type is correct and there is no evidence of twinning.

PLAT971_ALERT_2_C Check Calcd Resid. Dens. 1.23Ang From C5S   1.52 eA-3

Author Response: The atom type is correct and there is no evidence of twinning.

PLAT972_ALERT_2_C Check Calcd Resid. Dens. 0.57Ang From Pd6   -2.27 eA-3

Author Response: The atom type is correct and there is no evidence of twinning. The large residual density on Pd atoms may be Due to an anomalous dispersion effect and has no chemical significance.

PLAT972_ALERT_2_C Check Calcd Resid. Dens. 0.54Ang From Pd6   -2.23 eA-3

Author Response: The atom type is correct and there is no evidence of twinning. The large residual density on Pd atoms may be Due to an anomalous dispersion effect and has no chemical significance.

PLAT972_ALERT_2_C Check Calcd Resid. Dens. 1.02Ang From Pd3   -1.94 eA-3

Author Response: The atom type is correct and there is no evidence of twinning. The large residual density on Pd atoms may be Due to an anomalous dispersion effect and has no chemical significance.

PLAT972_ALERT_2_C Check Calcd Resid. Dens. 0.74Ang From Pd5   -1.75 eA-3

Author Response: The atom type is correct and there is no evidence of twinning. The large residual density on Pd atoms may be Due to an anomalous dispersion effect and has no chemical significance.

PLAT972_ALERT_2_C Check Calcd Resid. Dens. 0.83Ang From Pd3   -1.59 eA-3

Author Response: The atom type is correct and there is no evidence of twinning. The large residual density on Pd atoms may be Due to an anomalous dispersion effect and has no chemical significance.

PLAT972_ALERT_2_C Check Calcd Resid. Dens. 0.68Ang From Pd5   -1.58 eA-3

Author Response: The atom type is correct and there is no evidence of twinning. The large residual density on Pd atoms may be Due to an anomalous dispersion effect and has no chemical significance.

```
PLAT972_ALERT_2_C Check Calcd Resid. Dens.  0.76Ang From Pd5           -1.54 eA-3
```

Author Response: The atom type is correct and there is no evidence of twinning. The large residual density on Pd atoms may be Due to an anomalous dispersion effect and has no chemical significance.

```
PLAT977_ALERT_2_C Check Negative Difference Density on H16       .      -0.34 eA-3
PLAT977_ALERT_2_C Check Negative Difference Density on H18       .      -0.39 eA-3
PLAT977_ALERT_2_C Check Negative Difference Density on H28A      .      -0.45 eA-3
PLAT977_ALERT_2_C Check Negative Difference Density on H41       .      -0.36 eA-3
```

Alert level G

```
CELLZ01_ALERT_1_G Difference between formula and atom_site contents detected.
CELLZ01_ALERT_1_G ALERT: check formula stoichiometry or atom site occupancies.
           From the CIF: _cell_formula_units_Z    4
           From the CIF: _chemical_formula_sum  C94.86 H98.39 Cl12 N15.53 O5.71 Pd
           TEST: Compare cell contents of formula and atom_site data

           atom    Z*formula  cif sites diff
           C        379.44    379.41    0.03
           H        393.56    393.56    0.00
           Cl        48.00     48.00    0.00
           N         62.12     62.15   -0.03
           O         22.84     22.85   -0.01
           Pd        24.00     24.00    0.00
PLAT002_ALERT_2_G Number of Distance or Angle Restraints on AtSite         21 Note
PLAT003_ALERT_2_G Number of Uiso or Uij Restrained non-H Atoms ...         39 Report
PLAT007_ALERT_5_G Number of Unrefined Donor-H Atoms .............          12 Report
PLAT045_ALERT_1_G Calculated and Reported Z Differ by a Factor ...          2 Check
PLAT068_ALERT_1_G Reported F000 Differs from Calcd (or Missing)...     Please Check
PLAT072_ALERT_2_G SHELXL First  Parameter in WGHT  Unusually Large       0.12 Report
PLAT083_ALERT_2_G SHELXL Second Parameter in WGHT  Unusually Large     302.07 Why ?
PLAT142_ALERT_4_G s.u. on b - Axis Small or Missing .............     0.00050 Ang.
PLAT143_ALERT_4_G s.u. on c - Axis Small or Missing .............     0.00008 Ang.
PLAT172_ALERT_4_G The CIF-Embedded .res File Contains DFIX Records         17 Report
PLAT174_ALERT_4_G The CIF-Embedded .res File Contains FLAT Records          3 Report
PLAT178_ALERT_4_G The CIF-Embedded .res File Contains SIMU Records         11 Report
PLAT186_ALERT_4_G The CIF-Embedded .res File Contains ISOR Records          7 Report
PLAT187_ALERT_4_G The CIF-Embedded .res File Contains RIGU Records         14 Report
PLAT188_ALERT_3_G A Non-default SIMU Restraint Value has been used     0.0100 Report
PLAT188_ALERT_3_G A Non-default SIMU Restraint Value has been used     0.0200 Report
PLAT188_ALERT_3_G A Non-default SIMU Restraint Value has been used     0.0200 Report
PLAT188_ALERT_3_G A Non-default SIMU Restraint Value has been used     0.0200 Report
PLAT188_ALERT_3_G A Non-default SIMU Restraint Value has been used     0.0100 Report
PLAT188_ALERT_3_G A Non-default SIMU Restraint Value has been used     0.0200 Report
PLAT188_ALERT_3_G A Non-default SIMU Restraint Value has been used     0.0200 Report
PLAT188_ALERT_3_G A Non-default SIMU Restraint Value has been used     0.0200 Report
PLAT188_ALERT_3_G A Non-default SIMU Restraint Value has been used     0.0200 Report
PLAT190_ALERT_3_G A Non-default RIGU Restraint Value for First Par     0.0010 Report
PLAT190_ALERT_3_G A Non-default RIGU Restraint Value for SecondPar     0.0010 Report
PLAT190_ALERT_3_G A Non-default RIGU Restraint Value for First Par     0.0020 Report
PLAT190_ALERT_3_G A Non-default RIGU Restraint Value for SecondPar     0.0020 Report
PLAT190_ALERT_3_G A Non-default RIGU Restraint Value for First Par     0.0020 Report
PLAT190_ALERT_3_G A Non-default RIGU Restraint Value for SecondPar     0.0020 Report
```

```
PLAT190_ALERT_3_G A Non-default RIGU Restraint Value for First Par     0.0020 Report
PLAT190_ALERT_3_G A Non-default RIGU Restraint Value for SecondPar     0.0020 Report
PLAT190_ALERT_3_G A Non-default RIGU Restraint Value for First Par     0.0020 Report
PLAT190_ALERT_3_G A Non-default RIGU Restraint Value for SecondPar     0.0020 Report
PLAT190_ALERT_3_G A Non-default RIGU Restraint Value for First Par     0.0010 Report
PLAT190_ALERT_3_G A Non-default RIGU Restraint Value for SecondPar     0.0010 Report
PLAT190_ALERT_3_G A Non-default RIGU Restraint Value for First Par     0.0020 Report
PLAT190_ALERT_3_G A Non-default RIGU Restraint Value for SecondPar     0.0020 Report
PLAT190_ALERT_3_G A Non-default RIGU Restraint Value for First Par     0.0020 Report
PLAT190_ALERT_3_G A Non-default RIGU Restraint Value for SecondPar     0.0020 Report
PLAT190_ALERT_3_G A Non-default RIGU Restraint Value for First Par     0.0020 Report
PLAT190_ALERT_3_G A Non-default RIGU Restraint Value for SecondPar     0.0020 Report
PLAT190_ALERT_3_G A Non-default RIGU Restraint Value for First Par     0.0020 Report
PLAT190_ALERT_3_G A Non-default RIGU Restraint Value for SecondPar     0.0020 Report
PLAT190_ALERT_3_G A Non-default RIGU Restraint Value for First Par     0.0020 Report
PLAT190_ALERT_3_G A Non-default RIGU Restraint Value for SecondPar     0.0020 Report
PLAT190_ALERT_3_G A Non-default RIGU Restraint Value for First Par     0.0020 Report
PLAT190_ALERT_3_G A Non-default RIGU Restraint Value for SecondPar     0.0020 Report
PLAT232_ALERT_2_G Hirshfeld Test Diff (M-X)  Pd6      --N11      .        6.6 s.u.
PLAT300_ALERT_4_G Atom Site Occupancy of N3S        Constrained at        0.5 Check
PLAT300_ALERT_4_G Atom Site Occupancy of C5S        Constrained at        0.5 Check
PLAT300_ALERT_4_G Atom Site Occupancy of C6S        Constrained at        0.5 Check
PLAT300_ALERT_4_G Atom Site Occupancy of H6SA       Constrained at        0.5 Check
PLAT300_ALERT_4_G Atom Site Occupancy of H6SB       Constrained at        0.5 Check
PLAT300_ALERT_4_G Atom Site Occupancy of H6SC       Constrained at        0.5 Check
PLAT300_ALERT_4_G Atom Site Occupancy of N4S        Constrained at        0.5 Check
PLAT300_ALERT_4_G Atom Site Occupancy of C7S        Constrained at        0.5 Check
PLAT300_ALERT_4_G Atom Site Occupancy of C8S        Constrained at        0.5 Check
PLAT300_ALERT_4_G Atom Site Occupancy of H8SA       Constrained at        0.5 Check
PLAT300_ALERT_4_G Atom Site Occupancy of H8SB       Constrained at        0.5 Check
PLAT300_ALERT_4_G Atom Site Occupancy of H8SC       Constrained at        0.5 Check
PLAT300_ALERT_4_G Atom Site Occupancy of N7S        Constrained at        0.5 Check
PLAT300_ALERT_4_G Atom Site Occupancy of C13S       Constrained at        0.5 Check
PLAT300_ALERT_4_G Atom Site Occupancy of C14S       Constrained at        0.5 Check
PLAT300_ALERT_4_G Atom Site Occupancy of H14C       Constrained at        0.5 Check
PLAT300_ALERT_4_G Atom Site Occupancy of H14D       Constrained at        0.5 Check
PLAT300_ALERT_4_G Atom Site Occupancy of H14E       Constrained at        0.5 Check
PLAT300_ALERT_4_G Atom Site Occupancy of N5S        Constrained at     0.3333 Check
PLAT300_ALERT_4_G Atom Site Occupancy of C9S        Constrained at     0.3333 Check
PLAT300_ALERT_4_G Atom Site Occupancy of C10S       Constrained at     0.3333 Check
PLAT300_ALERT_4_G Atom Site Occupancy of H10B       Constrained at     0.3333 Check
PLAT300_ALERT_4_G Atom Site Occupancy of H10C       Constrained at     0.3333 Check
PLAT300_ALERT_4_G Atom Site Occupancy of H10D       Constrained at     0.3333 Check
PLAT300_ALERT_4_G Atom Site Occupancy of N6S        Constrained at     0.3333 Check
PLAT300_ALERT_4_G Atom Site Occupancy of C11S       Constrained at     0.3333 Check
PLAT300_ALERT_4_G Atom Site Occupancy of C12S       Constrained at     0.3333 Check
PLAT300_ALERT_4_G Atom Site Occupancy of H12B       Constrained at     0.3333 Check
PLAT300_ALERT_4_G Atom Site Occupancy of H12C       Constrained at     0.3333 Check
PLAT300_ALERT_4_G Atom Site Occupancy of H12D       Constrained at     0.3333 Check
PLAT300_ALERT_4_G Atom Site Occupancy of O4W        Constrained at        0.5 Check
PLAT300_ALERT_4_G Atom Site Occupancy of O5W        Constrained at        0.5 Check
PLAT300_ALERT_4_G Atom Site Occupancy of O6W        Constrained at        0.5 Check
PLAT300_ALERT_4_G Atom Site Occupancy of O2W        Constrained at     0.3333 Check
PLAT300_ALERT_4_G Atom Site Occupancy of O7W        Constrained at       0.25 Check
PLAT300_ALERT_4_G Atom Site Occupancy of O8W        Constrained at       0.25 Check
PLAT300_ALERT_4_G Atom Site Occupancy of O9W        Constrained at       0.25 Check
PLAT300_ALERT_4_G Atom Site Occupancy of O10W       Constrained at       0.25 Check
```

```
PLAT300_ALERT_4_G Atom Site Occupancy of O11W       Constrained at       0.25 Check
PLAT302_ALERT_4_G Anion/Solvent/Minor-Residue Disorder (Resd  3  )       100% Note
PLAT302_ALERT_4_G Anion/Solvent/Minor-Residue Disorder (Resd  5  )       100% Note
PLAT302_ALERT_4_G Anion/Solvent/Minor-Residue Disorder (Resd  6  )       100% Note
PLAT302_ALERT_4_G Anion/Solvent/Minor-Residue Disorder (Resd  7  )       100% Note
PLAT302_ALERT_4_G Anion/Solvent/Minor-Residue Disorder (Resd  8  )       100% Note
PLAT302_ALERT_4_G Anion/Solvent/Minor-Residue Disorder (Resd  9  )       100% Note
PLAT302_ALERT_4_G Anion/Solvent/Minor-Residue Disorder (Resd 10  )       100% Note
PLAT302_ALERT_4_G Anion/Solvent/Minor-Residue Disorder (Resd 12  )       100% Note
PLAT302_ALERT_4_G Anion/Solvent/Minor-Residue Disorder (Resd 13  )       100% Note
PLAT302_ALERT_4_G Anion/Solvent/Minor-Residue Disorder (Resd 14  )       100% Note
PLAT302_ALERT_4_G Anion/Solvent/Minor-Residue Disorder (Resd 15  )       100% Note
PLAT302_ALERT_4_G Anion/Solvent/Minor-Residue Disorder (Resd 16  )       100% Note
PLAT302_ALERT_4_G Anion/Solvent/Minor-Residue Disorder (Resd 17  )       100% Note
PLAT302_ALERT_4_G Anion/Solvent/Minor-Residue Disorder (Resd 18  )       100% Note
PLAT302_ALERT_4_G Anion/Solvent/Minor-Residue Disorder (Resd 19  )       100% Note
PLAT302_ALERT_4_G Anion/Solvent/Minor-Residue Disorder (Resd 20  )       100% Note
PLAT302_ALERT_4_G Anion/Solvent/Minor-Residue Disorder (Resd 21  )       100% Note
PLAT304_ALERT_4_G Non-Integer Number of Atoms in ..... (Resd  3  )       8.82 Check
PLAT304_ALERT_4_G Non-Integer Number of Atoms in ..... (Resd  8  )       2.22 Check
PLAT304_ALERT_4_G Non-Integer Number of Atoms in ..... (Resd 12  )       0.50 Check
PLAT304_ALERT_4_G Non-Integer Number of Atoms in ..... (Resd 13  )       0.50 Check
PLAT304_ALERT_4_G Non-Integer Number of Atoms in ..... (Resd 14  )       0.50 Check
PLAT304_ALERT_4_G Non-Integer Number of Atoms in ..... (Resd 15  )       0.37 Check
PLAT304_ALERT_4_G Non-Integer Number of Atoms in ..... (Resd 16  )       0.33 Check
PLAT304_ALERT_4_G Non-Integer Number of Atoms in ..... (Resd 17  )       0.25 Check
PLAT304_ALERT_4_G Non-Integer Number of Atoms in ..... (Resd 18  )       0.25 Check
PLAT304_ALERT_4_G Non-Integer Number of Atoms in ..... (Resd 19  )       0.25 Check
PLAT304_ALERT_4_G Non-Integer Number of Atoms in ..... (Resd 20  )       0.25 Check
PLAT304_ALERT_4_G Non-Integer Number of Atoms in ..... (Resd 21  )       0.25 Check
PLAT311_ALERT_2_G Isolated Disordered Oxygen Atom (No H's ?) .....        O4W Check
PLAT311_ALERT_2_G Isolated Disordered Oxygen Atom (No H's ?) .....        O5W Check
PLAT311_ALERT_2_G Isolated Disordered Oxygen Atom (No H's ?) .....        O6W Check
PLAT311_ALERT_2_G Isolated Disordered Oxygen Atom (No H's ?) .....        O1W Check
PLAT311_ALERT_2_G Isolated Disordered Oxygen Atom (No H's ?) .....        O2W Check
PLAT311_ALERT_2_G Isolated Disordered Oxygen Atom (No H's ?) .....        O7W Check
PLAT311_ALERT_2_G Isolated Disordered Oxygen Atom (No H's ?) .....        O8W Check
PLAT311_ALERT_2_G Isolated Disordered Oxygen Atom (No H's ?) .....        O9W Check
PLAT311_ALERT_2_G Isolated Disordered Oxygen Atom (No H's ?) .....       O10W Check
PLAT311_ALERT_2_G Isolated Disordered Oxygen Atom (No H's ?) .....       O11W Check
PLAT398_ALERT_2_G Deviating  C-O-C   Angle From 120 for O2A      .      107.9 Degree
PLAT432_ALERT_2_G Short Inter X...Y Contact  O3W      ..C10S     .       2.93 Ang.
                                                     x,y,z  =      1_555 Check
PLAT720_ALERT_4_G Number of Unusual/Non-Standard Labels ..........         17 Note
PLAT790_ALERT_4_G Centre of Gravity not Within Unit Cell: Resd.  #         18 Note
              O
PLAT790_ALERT_4_G Centre of Gravity not Within Unit Cell: Resd.  #         20 Note
              O
PLAT790_ALERT_4_G Centre of Gravity not Within Unit Cell: Resd.  #         21 Note
              O
PLAT793_ALERT_4_G Model has Chirality at N1          (Centro SPGR)          S Verify
PLAT793_ALERT_4_G Model has Chirality at N2          (Centro SPGR)          R Verify
PLAT793_ALERT_4_G Model has Chirality at N3          (Centro SPGR)          S Verify
PLAT793_ALERT_4_G Model has Chirality at N4          (Centro SPGR)          R Verify
PLAT793_ALERT_4_G Model has Chirality at N5          (Centro SPGR)          S Verify
PLAT793_ALERT_4_G Model has Chirality at N6          (Centro SPGR)          R Verify
PLAT793_ALERT_4_G Model has Chirality at N7          (Centro SPGR)          R Verify
```

```
PLAT793_ALERT_4_G Model has Chirality at N8          (Centro SPGR)          S Verify
PLAT793_ALERT_4_G Model has Chirality at N9          (Centro SPGR)          S Verify
PLAT793_ALERT_4_G Model has Chirality at N10         (Centro SPGR)          R Verify
PLAT793_ALERT_4_G Model has Chirality at N11         (Centro SPGR)          R Verify
PLAT793_ALERT_4_G Model has Chirality at N12         (Centro SPGR)          S Verify
PLAT794_ALERT_5_G Tentative Bond Valency for Pd1       (II)      .       2.03 Info
PLAT794_ALERT_5_G Tentative Bond Valency for Pd2       (II)      .       2.15 Info
PLAT794_ALERT_5_G Tentative Bond Valency for Pd3       (II)      .       2.04 Info
PLAT794_ALERT_5_G Tentative Bond Valency for Pd4       (II)      .       2.01 Info
PLAT794_ALERT_5_G Tentative Bond Valency for Pd5       (II)      .       2.13 Info
PLAT794_ALERT_5_G Tentative Bond Valency for Pd6       (II)      .       2.27 Info
PLAT860_ALERT_3_G Number of Least-Squares Restraints .............        530 Note
PLAT883_ALERT_1_G No Info/Value for _atom_sites_solution_primary .     Please Do !
PLAT910_ALERT_3_G Missing # of FCF Reflection(s) Below Theta(Min).          3 Note
PLAT912_ALERT_4_G Missing # of FCF Reflections Above STh/L=  0.600         12 Note
PLAT913_ALERT_3_G Missing # of Very Strong Reflections in FCF ....          2 Note
PLAT933_ALERT_2_G Number of HKL-OMIT Records in Embedded .res File          5 Note
PLAT978_ALERT_2_G Number C-C Bonds with Positive Residual Density.          0 Info
```

---

3 **ALERT level A** = Most likely a serious problem - resolve or explain
8 **ALERT level B** = A potentially serious problem, consider carefully
61 **ALERT level C** = Check. Ensure it is not caused by an omission or oversight
159 **ALERT level G** = General information/check it is not something unexpected

8 ALERT type 1 CIF construction/syntax error, inconsistent or missing data
81 ALERT type 2 Indicator that the structure model may be wrong or deficient
42 ALERT type 3 Indicator that the structure quality may be low
93 ALERT type 4 Improvement, methodology, query or suggestion
7 ALERT type 5 Informative message, check

It is advisable to attempt to resolve as many as possible of the alerts in all categories. Often the minor alerts point to easily fixed oversights, errors and omissions in your CIF or refinement strategy, so attention to these fine details can be worthwhile. In order to resolve some of the more serious problems it may be necessary to carry out additional measurements or structure refinements. However, the purpose of your study may justify the reported deviations and the more serious of these should normally be commented upon in the discussion or experimental section of a paper or in the "special_details" fields of the CIF. checkCIF was carefully designed to identify outliers and unusual parameters, but every test has its limitations and alerts that are not important in a particular case may appear. Conversely, the absence of alerts does not guarantee there are no aspects of the results needing attention. It is up to the individual to critically assess their own results and, if necessary, seek expert advice.

Publication of your CIF in IUCr journals

A basic structural check has been run on your CIF. These basic checks will be run on all CIFs submitted for publication in IUCr journals (*Acta Crystallographica*, *Journal of Applied Crystallography*, *Journal of Synchrotron Radiation*); however, if you intend to submit to *Acta Crystallographica Section C* or *E* or *IUCrData*, you should make sure that full publication checks are run on the final version of your CIF prior to submission.

Publication of your CIF in other journals

Please refer to the *Notes for Authors* of the relevant journal for any special instructions relating to CIF submission.

PLATON version of 28/11/2022; check.def file version of 28/11/2022

Datablock Acfuran-MeCN@MMF - ellipsoid plot

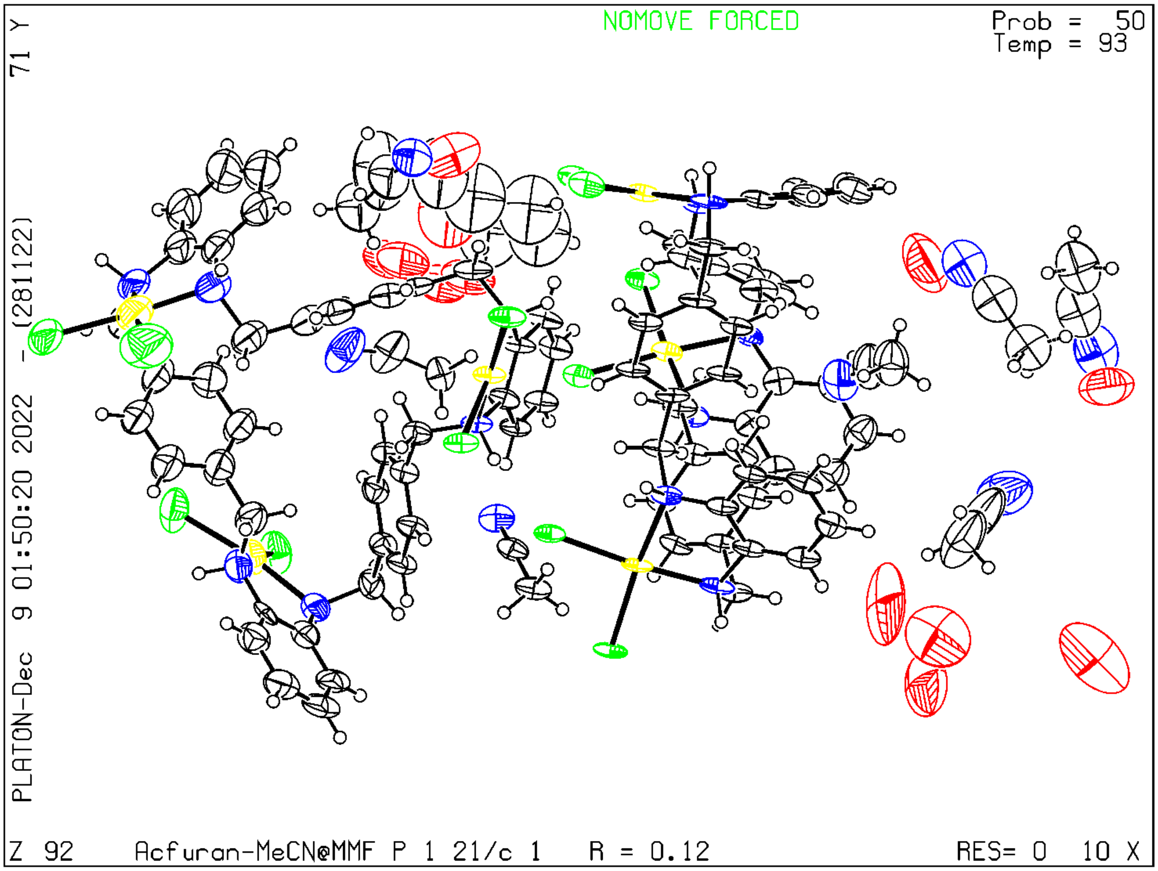

Supplement: Supplementary file 2 — Supporting Information [file SMLL-20-2405507-s001.zip › Acfuran-MeCN@MMFcheckcif.pdf]
