# Supplementary material for: Porous Supramolecular Crystalline Probe that Detects Non‐Covalent Interactions Involved in Molecular Recognition of Furanic Compounds
Source: Small. 2024 Jul 30;20(49):2405507. doi: 10.1002/smll.202405507 (PMC11618713; doi:10.1002/smll.202405507)

checkCIF/PLATON report

Structure factors have been supplied for datablock(s) furan-MeCN-MMF

THIS REPORT IS FOR GUIDANCE ONLY. IF USED AS PART OF A REVIEW PROCEDURE FOR PUBLICATION, IT SHOULD NOT REPLACE THE EXPERTISE OF AN EXPERIENCED CRYSTALLOGRAPHIC REFEREE.

No syntax errors found. CIF dictionary Interpreting this report

Datablock: furan-MeCN-MMF

Bond precision:	C-C = 0.0299 A		Wavelength=1.54184
Cell:	a=19.57180(14)	b=51.7499(5)	c=14.2509(1)
	alpha=90	beta=90.8846(6)	gamma=90
Temperature:	93 K		

	Calculated	Reported
Volume	14432.1(2)	14432.1(2)
Space group	P 21/c	P 1 21/c 1
Hall group	-P 2ybc	-P 2ybc
Moiety formula	8(C42 H42 Cl6 N6 Pd3), 17(C2 H3 N), ***(O)	2(C42 H42 Cl6 N6 Pd3), 4.25(C2 H3 N), 8.133(O)
Sum formula	C370 H387 Cl48 N65 O32.53 Pd24	C92.50 H96.75 Cl12 N16.25 O8.13 Pd6
Mr	10520.20	2630.03
Dx,g cm-3	1.211	1.210
Z	1	4
Mu (mm-1)	8.308	8.308
F000	5242.3	5242.0
F000'	5274.02	
h,k,lmax	23,62,17	23,62,17
Nref	26424	26292
Tmin,Tmax	0.153,0.489	0.202,1.000
Tmin'	0.028	

Correction method= # Reported T Limits: Tmin=0.202 Tmax=1.000
AbsCorr = MULTI-SCAN

Data completeness= 0.995 Theta(max)= 68.249

R(reflections)= 0.1522(22585) wR2(reflections)= 0.3792(26292)

S = 1.089 Npar= 1306

The following ALERTS were generated. Each ALERT has the format
test-name_ALERT_alert-type_alert-level.
Click on the hyperlinks for more details of the test.

Alert level A

PLAT602_ALERT_2_A Solvent Accessible VOID(S) in Structure .... ! Check

Author Response: Some solvents in the large pore could not be located due to severe disordering.

PLAT971_ALERT_2_A Check Calcd Resid. Dens. 1.11Ang From Pd1 6.00 eA-3

Author Response: The atom type is correct and there is no evidence of twinning. The large residual density on Pd atoms may be Due to an anomalous dispersion effect and has no chemical significance.

PLAT971_ALERT_2_A Check Calcd Resid. Dens. 1.05Ang From Pd4 5.08 eA-3

Author Response: The atom type is correct and there is no evidence of twinning. The large residual density on Pd atoms may be Due to an anomalous dispersion effect and has no chemical significance.

PLAT971_ALERT_2_A Check Calcd Resid. Dens. 1.06Ang From Pd3 4.80 eA-3

Author Response: The atom type is correct and there is no evidence of twinning. The large residual density on Pd atoms may be Due to an anomalous dispersion effect and has no chemical significance.

PLAT971_ALERT_2_A Check Calcd Resid. Dens. 0.94Ang From Pd3 4.35 eA-3

Author Response: The atom type is correct and there is no evidence of twinning. The large residual density on Pd atoms may be Due to an anomalous dispersion effect and has no chemical significance.

PLAT971_ALERT_2_A Check Calcd Resid. Dens. 1.11Ang From Pd2 4.34 eA-3

Author Response: The atom type is correct and there is no evidence of twinning. The large residual density on Pd atoms may be Due to an anomalous dispersion effect and has no chemical significance.

PLAT971_ALERT_2_A Check Calcd Resid. Dens. 0.94Ang From Pd1 4.30 eA-3

Author Response: The atom type is correct and there is no evidence of twinning. The large residual density on Pd atoms may be Due to an anomalous dispersion effect and has no chemical significance.

PLAT971_ALERT_2_A Check Calcd Resid. Dens. 1.19Ang From Pd4 4.06 eA-3

Author Response: The atom type is correct and there is no evidence of twinning. The large residual density on Pd atoms may be Due to an anomalous dispersion effect and has no chemical significance.

Alert level B

PLAT084_ALERT_3_B High wR2 Value (i.e. > 0.25) .... 0.38 Report

Author Response: Some solvents and guests in the large pore could not be located due to severe disordering. Therefore, the data quality was not high enough.

PLAT097_ALERT_2_B Large Reported Max. (Positive) Residual Density 5.27 eA-3

Author Response: The residual density is located between Pd and Cl atoms. Therefore, this may be due to an anomalous dispersion effect.

PLAT306_ALERT_2_B Isolated Oxygen Atom (H-atoms Missing ?) .... O1W Check

Author Response: Hydrogen atoms of water molecules could not be located in the difference electron density maps.

PLAT306_ALERT_2_B Isolated Oxygen Atom (H-atoms Missing ?) .... O4W Check

Author Response: Hydrogen atoms of water molecules could not be located in the difference electron density maps.

PLAT342_ALERT_3_B Low Bond Precision on C-C Bonds .... 0.02985 Ang.

Author Response: Some solvents and guests in the large pore could not be located due to severe disordering. Therefore, the data quality was not high enough.

```
PLAT971_ALERT_2_B Check Calcd Resid. Dens.  1.11Ang From Pd5            3.29 eA-3
```

Author Response: The atom type is correct and there is no evidence of twinning. The large residual density on Pd atoms may be Due to an anomalous dispersion effect and has no chemical significance.

```
PLAT971_ALERT_2_B Check Calcd Resid. Dens.  1.03Ang From Pd2            2.98 eA-3
```

Author Response: The atom type is correct and there is no evidence of twinning. The large residual density on Pd atoms may be Due to an anomalous dispersion effect and has no chemical significance.

```
PLAT971_ALERT_2_B Check Calcd Resid. Dens.  1.20Ang From Pd5            2.69 eA-3
```

Author Response: The atom type is correct and there is no evidence of twinning. The large residual density on Pd atoms may be Due to an anomalous dispersion effect and has no chemical significance.

```
PLAT972_ALERT_2_B Check Calcd Resid. Dens.  0.81Ang From Pd6           -2.77 eA-3
```

Author Response: The atom type is correct and there is no evidence of twinning. The large residual density on Pd atoms may be Due to an anomalous dispersion effect and has no chemical significance.

Alert level C

```
DIFMX02_ALERT_1_C  The maximum difference density is > 0.1*ZMAX*0.75
            The relevant atom site should be identified.
PLAT042_ALERT_1_C Calc. and Reported MoietyFormula Strings  Differ     Please Check
PLAT077_ALERT_4_C Unitcell Contains Non-integer Number of Atoms ..     Please Check
PLAT082_ALERT_2_C High R1 Value ..................................       0.15 Report
PLAT094_ALERT_2_C Ratio of Maximum / Minimum Residual Density ....       2.12 Report
PLAT213_ALERT_2_C Atom N1              has ADP max/min Ratio .....        3.2 prolat
PLAT213_ALERT_2_C Atom C34             has ADP max/min Ratio .....        3.2 oblate
PLAT213_ALERT_2_C Atom C62             has ADP max/min Ratio .....        3.3 oblate
PLAT220_ALERT_2_C NonSolvent   Resd 1  C   Ueq(max)/Ueq(min) Range        3.1 Ratio
PLAT220_ALERT_2_C NonSolvent   Resd 2  C   Ueq(max)/Ueq(min) Range        3.6 Ratio
PLAT220_ALERT_2_C NonSolvent   Resd 2 Cl   Ueq(max)/Ueq(min) Range        3.4 Ratio
PLAT234_ALERT_4_C Large Hirshfeld Difference C8       --C13      .       0.19 Ang.
PLAT234_ALERT_4_C Large Hirshfeld Difference C17      --C18      .       0.20 Ang.
PLAT234_ALERT_4_C Large Hirshfeld Difference C19      --C20      .       0.24 Ang.
PLAT234_ALERT_4_C Large Hirshfeld Difference C29      --C30      .       0.22 Ang.
PLAT234_ALERT_4_C Large Hirshfeld Difference Pd4      --N7       .       0.16 Ang.
PLAT234_ALERT_4_C Large Hirshfeld Difference Pd5      --N10      .       0.21 Ang.
PLAT234_ALERT_4_C Large Hirshfeld Difference N10      --C62      .       0.19 Ang.
PLAT234_ALERT_4_C Large Hirshfeld Difference C78      --C79      .       0.24 Ang.
PLAT241_ALERT_2_C High   'MainMol' Ueq as Compared to Neighbors of        C19 Check
PLAT241_ALERT_2_C High   'MainMol' Ueq as Compared to Neighbors of        C31 Check
PLAT241_ALERT_2_C High   'MainMol' Ueq as Compared to Neighbors of        N10 Check
```

```
PLAT241_ALERT_2_C High   'MainMol' Ueq as Compared to Neighbors of        C61 Check
PLAT241_ALERT_2_C High   'MainMol' Ueq as Compared to Neighbors of        C70 Check
PLAT242_ALERT_2_C Low    'MainMol' Ueq as Compared to Neighbors of        C20 Check
PLAT242_ALERT_2_C Low    'MainMol' Ueq as Compared to Neighbors of        C62 Check
PLAT244_ALERT_4_C Low    'Solvent' Ueq as Compared to Neighbors of        C3S Check
PLAT250_ALERT_2_C Large U3/U1 Ratio for Average U(i,j) Tensor ....        2.6 Note
PLAT260_ALERT_2_C Large Average Ueq of Residue Including       N2S      0.130 Check
PLAT260_ALERT_2_C Large Average Ueq of Residue Including       N3S      0.158 Check
PLAT260_ALERT_2_C Large Average Ueq of Residue Including       N4S      0.113 Check
PLAT260_ALERT_2_C Large Average Ueq of Residue Including       N5S      0.154 Check
PLAT260_ALERT_2_C Large Average Ueq of Residue Including       O1W      0.138 Check
PLAT260_ALERT_2_C Large Average Ueq of Residue Including       O3W      0.200 Check
PLAT260_ALERT_2_C Large Average Ueq of Residue Including       O4W      0.170 Check
PLAT260_ALERT_2_C Large Average Ueq of Residue Including       O5W      0.266 Check
PLAT260_ALERT_2_C Large Average Ueq of Residue Including       O7W      0.188 Check
PLAT260_ALERT_2_C Large Average Ueq of Residue Including       O8W      0.217 Check
PLAT260_ALERT_2_C Large Average Ueq of Residue Including      O10W      0.196 Check
PLAT260_ALERT_2_C Large Average Ueq of Residue Including      O11W      0.294 Check
PLAT260_ALERT_2_C Large Average Ueq of Residue Including      O15W      0.271 Check
PLAT260_ALERT_2_C Large Average Ueq of Residue Including       O2W      0.210 Check
PLAT260_ALERT_2_C Large Average Ueq of Residue Including       O6W      0.262 Check
PLAT260_ALERT_2_C Large Average Ueq of Residue Including       O9W      0.284 Check
PLAT260_ALERT_2_C Large Average Ueq of Residue Including      O12W      0.254 Check
PLAT260_ALERT_2_C Large Average Ueq of Residue Including      O13W      0.251 Check
PLAT260_ALERT_2_C Large Average Ueq of Residue Including      O14W      0.247 Check
PLAT334_ALERT_2_C Small <C-C> Benzene Dist.   C15      -C20       .       1.35 Ang.
PLAT334_ALERT_2_C Small <C-C> Benzene Dist.   C57      -C62       .       1.37 Ang.
PLAT334_ALERT_2_C Small <C-C> Benzene Dist.   C71      -C76       .       1.37 Ang.
PLAT411_ALERT_2_C Short Inter H...H Contact  H58      ..H84B      .       2.10 Ang.
                                                   x,y,-1+z  =      1_554 Check
PLAT420_ALERT_2_C D-H Bond Without Acceptor  N7       --H7        .    Please Check
PLAT430_ALERT_2_C Short Inter D...A Contact  O1W      ..N3S       .       2.88 Ang.
                                                      x,y,z  =      1_555 Check
PLAT767_ALERT_4_C INS Embedded LIST 6 Instruction Should be LIST 4     Please Check
PLAT906_ALERT_3_C Large K Value in the Analysis of Variance ......     20.964 Check
PLAT906_ALERT_3_C Large K Value in the Analysis of Variance ......      4.652 Check
PLAT906_ALERT_3_C Large K Value in the Analysis of Variance ......      2.488 Check
PLAT911_ALERT_3_C Missing FCF Refl Between Thmin & STh/L=    0.600        127 Report
PLAT918_ALERT_3_C Reflection(s) with I(obs) much Smaller I(calc) .          1 Check
PLAT971_ALERT_2_C Check Calcd Resid. Dens.  0.77Ang From C20              2.31 eA-3
```

Author Response: The atom type is correct and there is no evidence of twinning. The large residual density on Pd atoms may be Due to an anomalous dispersion effect and has no chemical significance.

```
PLAT971_ALERT_2_C Check Calcd Resid. Dens.  1.01Ang From Pd6              1.87 eA-3
```

Author Response: The atom type is correct and there is no evidence of twinning. The large residual density on Pd atoms may be Due to an anomalous dispersion effect and has no chemical significance.

PLAT971_ALERT_2_C Check Calcd Resid. Dens. 1.06Ang From Pd6   1.87 eA-3

Author Response: The atom type is correct and there is no evidence of twinning. The large residual density on Pd atoms may be Due to an anomalous dispersion effect and has no chemical significance.

PLAT971_ALERT_2_C Check Calcd Resid. Dens. 1.14Ang From Pd6   1.85 eA-3

Author Response: The atom type is correct and there is no evidence of twinning. The large residual density on Pd atoms may be Due to an anomalous dispersion effect and has no chemical significance.

PLAT971_ALERT_2_C Check Calcd Resid. Dens. 1.14Ang From Cl4   1.76 eA-3

Author Response: The atom type is correct and there is no evidence of twinning. The large residual density on Pd atoms may be Due to an anomalous dispersion effect and has no chemical significance.

PLAT971_ALERT_2_C Check Calcd Resid. Dens. 1.08Ang From Cl1   1.71 eA-3

Author Response: The atom type is correct and there is no evidence of twinning. The large residual density on Pd atoms may be Due to an anomalous dispersion effect and has no chemical significance.

PLAT971_ALERT_2_C Check Calcd Resid. Dens. 0.93Ang From Cl6   1.67 eA-3

Author Response: The atom type is correct and there is no evidence of twinning. The large residual density on Pd atoms may be Due to an anomalous dispersion effect and has no chemical significance.

PLAT971_ALERT_2_C Check Calcd Resid. Dens. 1.03Ang From Cl3   1.56 eA-3

Author Response: The atom type is correct and there is no evidence of twinning. The large residual density on Pd atoms may be Due to an anomalous dispersion effect and has no chemical significance.

PLAT971_ALERT_2_C Check Calcd Resid. Dens. 0.91Ang From Cl2   1.53 eA-3

Author Response: The atom type is correct and there is no evidence of twinning. The large residual density on Pd atoms may be Due to an anomalous dispersion effect and has no chemical significance.

PLAT971_ALERT_2_C Check Calcd Resid. Dens. 0.19Ang From C62   1.51 eA-3

Author Response: The atom type is correct and there is no evidence of twinning. The large residual density on Pd atoms may be Due to an anomalous dispersion effect and has no chemical significance.

PLAT972_ALERT_2_C Check Calcd Resid. Dens. 0.63Ang From Pd6   -2.16 eA-3

Author Response: The atom type is correct and there is no evidence of twinning. The large residual density on Pd atoms may be Due to an anomalous dispersion effect and has no chemical significance.

PLAT972_ALERT_2_C Check Calcd Resid. Dens. 0.60Ang From Pd6   -2.02 eA-3

Author Response: The atom type is correct and there is no evidence of twinning. The large residual density on Pd atoms may be Due to an anomalous dispersion effect and has no chemical significance.

PLAT972_ALERT_2_C Check Calcd Resid. Dens. 0.51Ang From Pd6   -2.00 eA-3

Author Response: The atom type is correct and there is no evidence of twinning. The large residual density on Pd atoms may be Due to an anomalous dispersion effect and has no chemical significance.

PLAT972_ALERT_2_C Check Calcd Resid. Dens. 1.33Ang From C5   -1.83 eA-3

Author Response: The atom type is correct and there is no evidence of twinning. The large residual density on Pd atoms may be Due to an anomalous dispersion effect and has no chemical significance.

PLAT972_ALERT_2_C Check Calcd Resid. Dens. 0.70Ang From Pd5   -1.82 eA-3

Author Response: The atom type is correct and there is no evidence of twinning. The large residual density on Pd atoms may be Due to an anomalous dispersion effect and has no chemical significance.

PLAT972_ALERT_2_C Check Calcd Resid. Dens. 0.60Ang From Pd5   -1.74 eA-3

Author Response: The atom type is correct and there is no evidence of twinning. The large residual density on Pd atoms may be Due to an anomalous dispersion effect and has no chemical significance.

```
PLAT972_ALERT_2_C Check Calcd Resid. Dens.  0.60Ang From Pd5           -1.74 eA-3
```

Author Response: The atom type is correct and there is no evidence of twinning. The large residual density on Pd atoms may be Due to an anomalous dispersion effect and has no chemical significance.

```
PLAT972_ALERT_2_C Check Calcd Resid. Dens.  2.01Ang From C4            -1.61 eA-3
```

Author Response: The atom type is correct and there is no evidence of twinning. The large residual density on Pd atoms may be Due to an anomalous dispersion effect and has no chemical significance.

```
PLAT977_ALERT_2_C Check Negative Difference Density on H12A      .      -0.49 eA-3
PLAT977_ALERT_2_C Check Negative Difference Density on H28A      .      -0.33 eA-3
PLAT977_ALERT_2_C Check Negative Difference Density on H32       .      -0.42 eA-3
PLAT977_ALERT_2_C Check Negative Difference Density on H51       .      -0.39 eA-3
PLAT977_ALERT_2_C Check Negative Difference Density on H52       .      -0.36 eA-3
```

Alert level G

```
PLAT003_ALERT_2_G Number of Uiso or Uij Restrained non-H Atoms ...         23 Report
PLAT007_ALERT_5_G Number of Unrefined Donor-H Atoms .............         12 Report
PLAT045_ALERT_1_G Calculated and Reported Z Differ by a Factor ...      0.250 Check
PLAT068_ALERT_1_G Reported F000 Differs from Calcd (or Missing)...     Please Check
PLAT072_ALERT_2_G SHELXL First  Parameter in WGHT  Unusually Large       0.11 Report
PLAT083_ALERT_2_G SHELXL Second Parameter in WGHT  Unusually Large     507.25 Why ?
PLAT142_ALERT_4_G s.u. on b - Axis Small or Missing .............    0.00050 Ang.
PLAT143_ALERT_4_G s.u. on c - Axis Small or Missing .............    0.00010 Ang.
PLAT178_ALERT_4_G The CIF-Embedded .res File Contains SIMU Records          5 Report
PLAT186_ALERT_4_G The CIF-Embedded .res File Contains ISOR Records         11 Report
PLAT187_ALERT_4_G The CIF-Embedded .res File Contains RIGU Records          7 Report
PLAT188_ALERT_3_G A Non-default SIMU Restraint Value has been used     0.0200 Report
PLAT190_ALERT_3_G A Non-default RIGU Restraint Value for First Par     0.0020 Report
PLAT190_ALERT_3_G A Non-default RIGU Restraint Value for SecondPar     0.0020 Report
PLAT190_ALERT_3_G A Non-default RIGU Restraint Value for First Par     0.0020 Report
PLAT190_ALERT_3_G A Non-default RIGU Restraint Value for SecondPar     0.0020 Report
PLAT190_ALERT_3_G A Non-default RIGU Restraint Value for First Par     0.0020 Report
PLAT190_ALERT_3_G A Non-default RIGU Restraint Value for SecondPar     0.0020 Report
PLAT232_ALERT_2_G Hirshfeld Test Diff (M-X)  Pd1      --N1       .        5.6 s.u.
PLAT300_ALERT_4_G Atom Site Occupancy of N4S       Constrained at         0.5 Check
PLAT300_ALERT_4_G Atom Site Occupancy of C7S       Constrained at         0.5 Check
PLAT300_ALERT_4_G Atom Site Occupancy of C8S       Constrained at         0.5 Check
PLAT300_ALERT_4_G Atom Site Occupancy of H8SA      Constrained at         0.5 Check
PLAT300_ALERT_4_G Atom Site Occupancy of H8SB      Constrained at         0.5 Check
PLAT300_ALERT_4_G Atom Site Occupancy of H8SC      Constrained at         0.5 Check
PLAT300_ALERT_4_G Atom Site Occupancy of N5S       Constrained at        0.75 Check
PLAT300_ALERT_4_G Atom Site Occupancy of C9S       Constrained at        0.75 Check
PLAT300_ALERT_4_G Atom Site Occupancy of C10S      Constrained at        0.75 Check
PLAT300_ALERT_4_G Atom Site Occupancy of H10B      Constrained at        0.75 Check
PLAT300_ALERT_4_G Atom Site Occupancy of H10C      Constrained at        0.75 Check
PLAT300_ALERT_4_G Atom Site Occupancy of H10D      Constrained at        0.75 Check
PLAT300_ALERT_4_G Atom Site Occupancy of O5W       Constrained at         0.5 Check
PLAT300_ALERT_4_G Atom Site Occupancy of O10W      Constrained at         0.5 Check
```

```
PLAT300_ALERT_4_G Atom Site Occupancy of O11W       Constrained at        0.5 Check
PLAT300_ALERT_4_G Atom Site Occupancy of O15W       Constrained at        0.5 Check
PLAT300_ALERT_4_G Atom Site Occupancy of O14W       Constrained at     0.3333 Check
PLAT302_ALERT_4_G Anion/Solvent/Minor-Residue Disorder (Resd  6  )       100% Note
PLAT302_ALERT_4_G Anion/Solvent/Minor-Residue Disorder (Resd  7  )       100% Note
PLAT302_ALERT_4_G Anion/Solvent/Minor-Residue Disorder (Resd  9  )       100% Note
PLAT302_ALERT_4_G Anion/Solvent/Minor-Residue Disorder (Resd 11  )       100% Note
PLAT302_ALERT_4_G Anion/Solvent/Minor-Residue Disorder (Resd 12  )       100% Note
PLAT302_ALERT_4_G Anion/Solvent/Minor-Residue Disorder (Resd 13  )       100% Note
PLAT302_ALERT_4_G Anion/Solvent/Minor-Residue Disorder (Resd 14  )       100% Note
PLAT302_ALERT_4_G Anion/Solvent/Minor-Residue Disorder (Resd 15  )       100% Note
PLAT302_ALERT_4_G Anion/Solvent/Minor-Residue Disorder (Resd 16  )       100% Note
PLAT302_ALERT_4_G Anion/Solvent/Minor-Residue Disorder (Resd 17  )       100% Note
PLAT302_ALERT_4_G Anion/Solvent/Minor-Residue Disorder (Resd 18  )       100% Note
PLAT302_ALERT_4_G Anion/Solvent/Minor-Residue Disorder (Resd 19  )       100% Note
PLAT302_ALERT_4_G Anion/Solvent/Minor-Residue Disorder (Resd 20  )       100% Note
PLAT302_ALERT_4_G Anion/Solvent/Minor-Residue Disorder (Resd 21  )       100% Note
PLAT302_ALERT_4_G Anion/Solvent/Minor-Residue Disorder (Resd 22  )       100% Note
PLAT304_ALERT_4_G Non-Integer Number of Atoms in ..... (Resd  7  )       4.50 Check
PLAT304_ALERT_4_G Non-Integer Number of Atoms in ..... (Resd  9  )       0.62 Check
PLAT304_ALERT_4_G Non-Integer Number of Atoms in ..... (Resd 11  )       0.50 Check
PLAT304_ALERT_4_G Non-Integer Number of Atoms in ..... (Resd 12  )       0.51 Check
PLAT304_ALERT_4_G Non-Integer Number of Atoms in ..... (Resd 13  )       0.57 Check
PLAT304_ALERT_4_G Non-Integer Number of Atoms in ..... (Resd 14  )       0.50 Check
PLAT304_ALERT_4_G Non-Integer Number of Atoms in ..... (Resd 15  )       0.50 Check
PLAT304_ALERT_4_G Non-Integer Number of Atoms in ..... (Resd 16  )       0.50 Check
PLAT304_ALERT_4_G Non-Integer Number of Atoms in ..... (Resd 17  )       0.38 Check
PLAT304_ALERT_4_G Non-Integer Number of Atoms in ..... (Resd 18  )       0.49 Check
PLAT304_ALERT_4_G Non-Integer Number of Atoms in ..... (Resd 19  )       0.43 Check
PLAT304_ALERT_4_G Non-Integer Number of Atoms in ..... (Resd 20  )       0.49 Check
PLAT304_ALERT_4_G Non-Integer Number of Atoms in ..... (Resd 21  )       0.31 Check
PLAT304_ALERT_4_G Non-Integer Number of Atoms in ..... (Resd 22  )       0.33 Check
PLAT311_ALERT_2_G Isolated Disordered Oxygen Atom (No H's ?) .....        O3W Check
PLAT311_ALERT_2_G Isolated Disordered Oxygen Atom (No H's ?) .....        O5W Check
PLAT311_ALERT_2_G Isolated Disordered Oxygen Atom (No H's ?) .....        O7W Check
PLAT311_ALERT_2_G Isolated Disordered Oxygen Atom (No H's ?) .....        O8W Check
PLAT311_ALERT_2_G Isolated Disordered Oxygen Atom (No H's ?) .....       O10W Check
PLAT311_ALERT_2_G Isolated Disordered Oxygen Atom (No H's ?) .....       O11W Check
PLAT311_ALERT_2_G Isolated Disordered Oxygen Atom (No H's ?) .....       O15W Check
PLAT311_ALERT_2_G Isolated Disordered Oxygen Atom (No H's ?) .....        O2W Check
PLAT311_ALERT_2_G Isolated Disordered Oxygen Atom (No H's ?) .....        O6W Check
PLAT311_ALERT_2_G Isolated Disordered Oxygen Atom (No H's ?) .....        O9W Check
PLAT311_ALERT_2_G Isolated Disordered Oxygen Atom (No H's ?) .....       O12W Check
PLAT311_ALERT_2_G Isolated Disordered Oxygen Atom (No H's ?) .....       O13W Check
PLAT311_ALERT_2_G Isolated Disordered Oxygen Atom (No H's ?) .....       O14W Check
PLAT335_ALERT_2_G Check Large C6 Ring C-C Range C57      -C62            0.22 Ang.
PLAT335_ALERT_2_G Check Large C6 Ring C-C Range C71      -C76            0.21 Ang.
PLAT720_ALERT_4_G Number of Unusual/Non-Standard Labels ..........         12 Note
PLAT722_ALERT_1_G Angle   Calc      120.00, Rep      118.90 Dev...       1.10 Degree
             C75     -C74     -H74        1_555  1_555  1_555     #  364 Check
PLAT722_ALERT_1_G Angle   Calc      122.00, Rep      120.90 Dev...       1.10 Degree
             C74     -C73     -H73        1_555  1_555  1_555     #  403 Check
PLAT790_ALERT_4_G Centre of Gravity not Within Unit Cell: Resd.  #          6 Note
             C2 H3 N
PLAT790_ALERT_4_G Centre of Gravity not Within Unit Cell: Resd.  #         17 Note
             O
PLAT793_ALERT_4_G Model has Chirality at N1          (Centro SPGR)          S Verify
```

```
PLAT793_ALERT_4_G Model has Chirality at N2          (Centro SPGR)          R Verify
PLAT793_ALERT_4_G Model has Chirality at N3          (Centro SPGR)          S Verify
PLAT793_ALERT_4_G Model has Chirality at N4          (Centro SPGR)          R Verify
PLAT793_ALERT_4_G Model has Chirality at N5          (Centro SPGR)          S Verify
PLAT793_ALERT_4_G Model has Chirality at N6          (Centro SPGR)          R Verify
PLAT793_ALERT_4_G Model has Chirality at N7          (Centro SPGR)          R Verify
PLAT793_ALERT_4_G Model has Chirality at N8          (Centro SPGR)          S Verify
PLAT793_ALERT_4_G Model has Chirality at N9          (Centro SPGR)          S Verify
PLAT793_ALERT_4_G Model has Chirality at N10         (Centro SPGR)          R Verify
PLAT793_ALERT_4_G Model has Chirality at N11         (Centro SPGR)          R Verify
PLAT793_ALERT_4_G Model has Chirality at N12         (Centro SPGR)          S Verify
PLAT794_ALERT_5_G Tentative Bond Valency for Pd1       (II)      .       2.00 Info
PLAT794_ALERT_5_G Tentative Bond Valency for Pd2       (II)      .       2.14 Info
PLAT794_ALERT_5_G Tentative Bond Valency for Pd3       (II)      .       2.03 Info
PLAT794_ALERT_5_G Tentative Bond Valency for Pd4       (II)      .       1.98 Info
PLAT794_ALERT_5_G Tentative Bond Valency for Pd5       (II)      .       2.14 Info
PLAT794_ALERT_5_G Tentative Bond Valency for Pd6       (II)      .       2.14 Info
PLAT860_ALERT_3_G Number of Least-Squares Restraints .............        258 Note
PLAT883_ALERT_1_G No Info/Value for _atom_sites_solution_primary .     Please Do !
PLAT910_ALERT_3_G Missing # of FCF Reflection(s) Below Theta(Min).          3 Note
PLAT912_ALERT_4_G Missing # of FCF Reflections Above STh/L=  0.600          3 Note
PLAT913_ALERT_3_G Missing # of Very Strong Reflections in FCF ....          2 Note
PLAT933_ALERT_2_G Number of HKL-OMIT Records in Embedded .res File          3 Note
PLAT978_ALERT_2_G Number C-C Bonds with Positive Residual Density.          0 Info
```

```
   8 ALERT level A = Most likely a serious problem - resolve or explain
   9 ALERT level B = A potentially serious problem, consider carefully
  82 ALERT level C = Check. Ensure it is not caused by an omission or oversight
 110 ALERT level G = General information/check it is not something unexpected

   7 ALERT type 1 CIF construction/syntax error, inconsistent or missing data
 100 ALERT type 2 Indicator that the structure model may be wrong or deficient
  17 ALERT type 3 Indicator that the structure quality may be low
  78 ALERT type 4 Improvement, methodology, query or suggestion
   7 ALERT type 5 Informative message, check
```

It is advisable to attempt to resolve as many as possible of the alerts in all categories. Often the minor alerts point to easily fixed oversights, errors and omissions in your CIF or refinement strategy, so attention to these fine details can be worthwhile. In order to resolve some of the more serious problems it may be necessary to carry out additional measurements or structure refinements. However, the purpose of your study may justify the reported deviations and the more serious of these should normally be commented upon in the discussion or experimental section of a paper or in the "special_details" fields of the CIF. checkCIF was carefully designed to identify outliers and unusual parameters, but every test has its limitations and alerts that are not important in a particular case may appear. Conversely, the absence of alerts does not guarantee there are no aspects of the results needing attention. It is up to the individual to critically assess their own results and, if necessary, seek expert advice.

Publication of your CIF in IUCr journals

A basic structural check has been run on your CIF. These basic checks will be run on all CIFs submitted for publication in IUCr journals (*Acta Crystallographica*, *Journal of Applied Crystallography*, *Journal of Synchrotron Radiation*); however, if you intend to submit to *Acta Crystallographica Section C* or *E* or *IUCrData*, you should make sure that full publication checks are run on the final version of your CIF prior to submission.

Publication of your CIF in other journals

Please refer to the *Notes for Authors* of the relevant journal for any special instructions relating to CIF submission.

PLATON version of 28/11/2022; check.def file version of 28/11/2022

Datablock furan-MeCN-MMF - ellipsoid plot

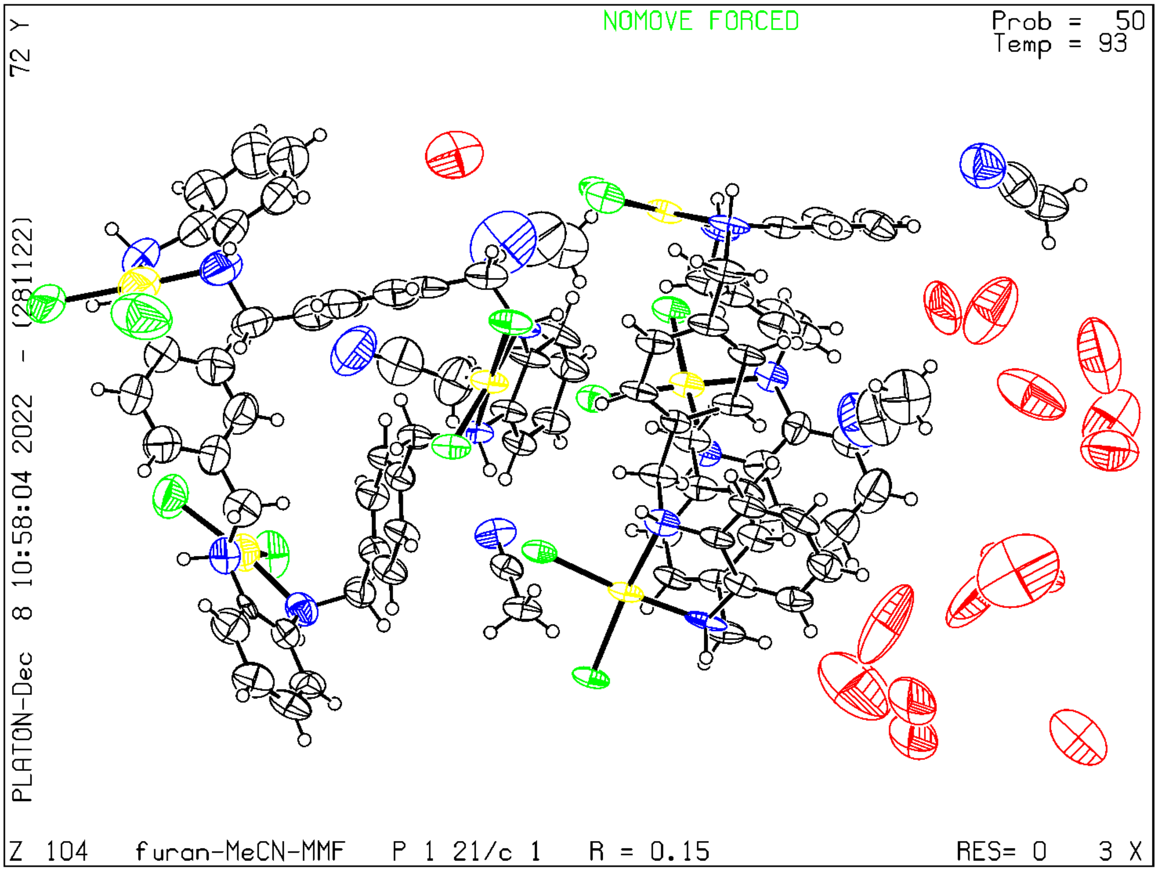

Supplement: Supplementary file 2 — Supporting Information [file SMLL-20-2405507-s001.zip › furan-MeCN@MMF_checkcif.pdf]
