# Supplementary material for: Porous Supramolecular Crystalline Probe that Detects Non‐Covalent Interactions Involved in Molecular Recognition of Furanic Compounds
Source: Small. 2024 Jul 30;20(49):2405507. doi: 10.1002/smll.202405507 (PMC11618713; doi:10.1002/smll.202405507)

checkCIF/PLATON report

Structure factors have been supplied for datablock(s) FAL-MeCN@MMF

THIS REPORT IS FOR GUIDANCE ONLY. IF USED AS PART OF A REVIEW PROCEDURE FOR PUBLICATION, IT SHOULD NOT REPLACE THE EXPERTISE OF AN EXPERIENCED CRYSTALLOGRAPHIC REFEREE.

No syntax errors found. CIF dictionary Interpreting this report

Datablock: FAL-MeCN@MMF

```
Bond precision:   C-C = 0.0120 A          Wavelength=1.54184

Cell:             a=19.57830(15)    b=52.1082(5)      c=14.30530(9)
                  alpha=90          beta=90.8158(6)   gamma=90
Temperature:      93 K
```

	Calculated	Reported
Volume	14592.6(2)	14592.6(2)
Space group	P 21/c	P 1 21/c 1
Hall group	-P 2ybc	-P 2ybc
Moiety formula	C42 H42 Cl6 N6 Pd3, 0.303(C6 H O), 0.198(C5 H4 O2), 0.198(C5),	2(C42 H42 Cl6 N6 Pd3), 4.664(C2 H3 N), 3.419(O), 0.395(C5 H4 O2
Sum formula	C50.45 H50.09 Cl6 N8.33 O2.41 Pd3	C100.91 H100.18 Cl12 N16.66 O4.82 Pd6
Mr	1343.58	2687.19
Dx,g cm-3	1.223	1.223
Z	8	4
Mu (mm-1)	8.211	8.211
F000	5363.1	5363.0
F000'	5394.80	
h,k,lmax	23,62,17	23,62,17
Nref	26734	26697
Tmin,Tmax	0.159,0.708	0.303,1.000
Tmin'	0.071	

```
Correction method= # Reported T Limits: Tmin=0.303 Tmax=1.000
AbsCorr = MULTI-SCAN

Data completeness= 0.999            Theta(max)= 68.249
```

R(reflections)= 0.1016(22964) wR2(reflections)= 0.2665(26697)

S = 1.137 Npar= 1278

The following ALERTS were generated. Each ALERT has the format
test-name_ALERT_alert-type_alert-level.
Click on the hyperlinks for more details of the test.

Alert level A

PLAT602_ALERT_2_A Solvent Accessible VOID(S) in Structure .... ! Check

Author Response: Some solvents in the large pore could not be located due to severe disordering.

Alert level B

PLAT306_ALERT_2_B Isolated Oxygen Atom (H-atoms Missing ?) .... O1W Check

Author Response: Hydrogen atoms of water molecules and OH groups of the guests could not be located in the difference electron density maps.

PLAT971_ALERT_2_B Check Calcd Resid. Dens. 1.01Ang From Pd6 3.00 eA-3

Author Response: The atom type is correct and there is no evidence of twinning.

PLAT971_ALERT_2_B Check Calcd Resid. Dens. 0.74Ang From C20 2.61 eA-3

Author Response: The atom type is correct and there is no evidence of twinning.

PLAT971_ALERT_2_B Check Calcd Resid. Dens. 0.53Ang From C62 2.54 eA-3

Author Response: The atom type is correct and there is no evidence of twinning.

PLAT972_ALERT_2_B Check Calcd Resid. Dens. 0.88Ang From Pd6 -2.90 eA-3

Author Response: The atom type is correct and there is no evidence of twinning. The large residual density on Pd atoms may be Due to an anomalous dispersion effect and has no chemical significance.

PLAT972_ALERT_2_B Check Calcd Resid. Dens. 0.42Ang From Pd6 -2.59 eA-3

Author Response: The atom type is correct and there is no evidence of twinning. The large residual density on Pd atoms may be Due to an anomalous dispersion effect and has no chemical significance.

Alert level C

```
PLAT041_ALERT_1_C Calc. and Reported SumFormula    Strings  Differ     Please Check
PLAT077_ALERT_4_C Unitcell Contains Non-integer Number of Atoms ..     Please Check
PLAT084_ALERT_3_C High wR2 Value (i.e. > 0.25) ..................       0.27 Report
PLAT213_ALERT_2_C Atom N3             has ADP max/min Ratio .....        3.8 prolat
PLAT213_ALERT_2_C Atom C20            has ADP max/min Ratio .....        3.4 prolat
PLAT220_ALERT_2_C NonSolvent   Resd 2  C   Ueq(max)/Ueq(min) Range       4.6 Ratio
PLAT220_ALERT_2_C NonSolvent   Resd 2 Cl   Ueq(max)/Ueq(min) Range       3.8 Ratio
PLAT220_ALERT_2_C NonSolvent   Resd 2  N   Ueq(max)/Ueq(min) Range       3.2 Ratio
PLAT234_ALERT_4_C Large Hirshfeld Difference C19      --C20      .      0.18 Ang.
PLAT234_ALERT_4_C Large Hirshfeld Difference Pd6      --N11      .      0.18 Ang.
PLAT234_ALERT_4_C Large Hirshfeld Difference N10      --C62      .      0.19 Ang.
PLAT241_ALERT_2_C High   'MainMol' Ueq as Compared to Neighbors of       C61 Check
PLAT241_ALERT_2_C High   'MainMol' Ueq as Compared to Neighbors of       C70 Check
PLAT242_ALERT_2_C Low    'MainMol' Ueq as Compared to Neighbors of       Pd6 Check
PLAT242_ALERT_2_C Low    'MainMol' Ueq as Compared to Neighbors of       C62 Check
PLAT244_ALERT_4_C Low    'Solvent' Ueq as Compared to Neighbors of       C3S Check
PLAT250_ALERT_2_C Large U3/U1 Ratio for Average U(i,j) Tensor ....       2.5 Note
PLAT250_ALERT_2_C Large U3/U1 Ratio for Average U(i,j) Tensor ....       2.4 Note
PLAT250_ALERT_2_C Large U3/U1 Ratio for Average U(i,j) Tensor ....       2.8 Note
PLAT260_ALERT_2_C Large Average Ueq of Residue Including      O1B      0.169 Check
PLAT260_ALERT_2_C Large Average Ueq of Residue Including      O1A      0.160 Check
PLAT260_ALERT_2_C Large Average Ueq of Residue Including      C1C      0.155 Check
PLAT260_ALERT_2_C Large Average Ueq of Residue Including      N2S      0.183 Check
PLAT260_ALERT_2_C Large Average Ueq of Residue Including      N3S      0.144 Check
PLAT260_ALERT_2_C Large Average Ueq of Residue Including      N4S      0.232 Check
PLAT260_ALERT_2_C Large Average Ueq of Residue Including      N5S      0.147 Check
PLAT260_ALERT_2_C Large Average Ueq of Residue Including      N7S      0.120 Check
PLAT260_ALERT_2_C Large Average Ueq of Residue Including      N6S      0.139 Check
PLAT260_ALERT_2_C Large Average Ueq of Residue Including      O1W      0.174 Check
PLAT260_ALERT_2_C Large Average Ueq of Residue Including      O2W      0.279 Check
PLAT260_ALERT_2_C Large Average Ueq of Residue Including      O3W      0.254 Check
PLAT260_ALERT_2_C Large Average Ueq of Residue Including      O4W      0.280 Check
PLAT260_ALERT_2_C Large Average Ueq of Residue Including      O6W      0.240 Check
PLAT342_ALERT_3_C Low Bond Precision on  C-C Bonds ...............    0.01195 Ang.
PLAT365_ALERT_2_C Long   C(sp3)-C(sp)  Bond  C3S      - C4S      .      1.61 Ang.
PLAT411_ALERT_2_C Short Inter H...H Contact  H58      ..H84A     .      2.11 Ang.
                                                    x,y,1+z  =      1_556 Check
PLAT420_ALERT_2_C D-H Bond Without Acceptor  N7       --H7       .     Please Check
PLAT767_ALERT_4_C INS Embedded LIST 6 Instruction Should be LIST 4     Please Check
PLAT906_ALERT_3_C Large K Value in the Analysis of Variance ......     10.171 Check
PLAT906_ALERT_3_C Large K Value in the Analysis of Variance ......      2.965 Check
PLAT911_ALERT_3_C Missing FCF Refl Between Thmin & STh/L=    0.600         25 Report
PLAT918_ALERT_3_C Reflection(s) with I(obs) much Smaller I(calc) .          4 Check
PLAT971_ALERT_2_C Check Calcd Resid. Dens.  0.96Ang From Pd1            2.47 eA-3
```

Author Response: The atom type is correct and there is no evidence of twinning.

PLAT971_ALERT_2_C Check Calcd Resid. Dens. 1.15Ang From Pd6 2.11 eA-3

Author Response: The atom type is correct and there is no evidence of twinning.

PLAT971_ALERT_2_C Check Calcd Resid. Dens. 1.04Ang From Pd4 2.05 eA-3

Author Response: The atom type is correct and there is no evidence of twinning.

PLAT971_ALERT_2_C Check Calcd Resid. Dens. 1.14Ang From Pd3 1.86 eA-3

Author Response: The atom type is correct and there is no evidence of twinning.

PLAT971_ALERT_2_C Check Calcd Resid. Dens. 1.08Ang From Pd2 1.85 eA-3

Author Response: The atom type is correct and there is no evidence of twinning.

PLAT971_ALERT_2_C Check Calcd Resid. Dens. 0.97Ang From Pd1 1.77 eA-3

Author Response: The atom type is correct and there is no evidence of twinning.

PLAT971_ALERT_2_C Check Calcd Resid. Dens. 1.09Ang From Pd3 1.70 eA-3

Author Response: The atom type is correct and there is no evidence of twinning.

PLAT971_ALERT_2_C Check Calcd Resid. Dens. 1.17Ang From Pd6 1.69 eA-3

Author Response: The atom type is correct and there is no evidence of twinning.

PLAT971_ALERT_2_C Check Calcd Resid. Dens. 0.40Ang From Pd1 1.52 eA-3

Author Response: The atom type is correct and there is no evidence of twinning.

PLAT971_ALERT_2_C Check Calcd Resid. Dens. 0.37Ang From Pd1 1.52 eA-3

Author Response: The atom type is correct and there is no evidence of twinning.

PLAT972_ALERT_2_C Check Calcd Resid. Dens. 0.70Ang From Pd6 -2.42 eA-3

Author Response: The atom type is correct and there is no evidence of twinning. The large residual density on Pd atoms may be Due to an anomalous dispersion effect and has no chemical significance.

```
PLAT972_ALERT_2_C Check Calcd Resid. Dens.  0.52Ang From Pd5           -1.57 eA-3
```

Author Response: The atom type is correct and there is no evidence of twinning. The large residual density on Pd atoms may be Due to an anomalous dispersion effect and has no chemical significance.

```
PLAT972_ALERT_2_C Check Calcd Resid. Dens.  0.68Ang From Pd5           -1.57 eA-3
```

Author Response: The atom type is correct and there is no evidence of twinning. The large residual density on Pd atoms may be Due to an anomalous dispersion effect and has no chemical significance.

```
PLAT972_ALERT_2_C Check Calcd Resid. Dens.  0.76Ang From Pd5           -1.56 eA-3
```

Author Response: The atom type is correct and there is no evidence of twinning. The large residual density on Pd atoms may be Due to an anomalous dispersion effect and has no chemical significance.

```
PLAT973_ALERT_2_C Check Calcd Positive Resid. Density on       Pd6       1.19 eA-3
PLAT977_ALERT_2_C Check Negative Difference Density on H4        .      -0.37 eA-3
PLAT977_ALERT_2_C Check Negative Difference Density on H28A      .      -0.35 eA-3
PLAT977_ALERT_2_C Check Negative Difference Density on H72       .      -0.32 eA-3
```

Alert level G

```
FORMU01_ALERT_1_G  There is a discrepancy between the atom counts in the
            _chemical_formula_sum and _chemical_formula_moiety. This is
            usually due to the moiety formula being in the wrong format.
            Atom count from _chemical_formula_sum:   C100.91 H100.18 Cl12 N16.66 O
            Atom count from _chemical_formula_moiety:C98.933 H99.57201 Cl12 N16.66
CELLZ01_ALERT_1_G Difference between formula and atom_site contents detected.
CELLZ01_ALERT_1_G ALERT: check formula stoichiometry or atom site occupancies.
           From the CIF: _cell_formula_units_Z    4
           From the CIF: _chemical_formula_sum  C100.91 H100.18 Cl12 N16.66 O4.82
           TEST: Compare cell contents of formula and atom_site data

           atom    Z*formula  cif sites diff
           C        403.64    403.63    0.01
           H        400.72    400.70    0.02
           Cl        48.00     48.00    0.00
           N         66.64     66.65   -0.01
           O         19.28     19.26    0.02
           Pd        24.00     24.00    0.00
PLAT002_ALERT_2_G Number of Distance or Angle Restraints on AtSite         11 Note
PLAT003_ALERT_2_G Number of Uiso or Uij Restrained non-H Atoms ...         26 Report
PLAT007_ALERT_5_G Number of Unrefined Donor-H Atoms .............         12 Report
PLAT045_ALERT_1_G Calculated and Reported Z Differ by a Factor ...          2 Check
PLAT068_ALERT_1_G Reported F000 Differs from Calcd (or Missing)...     Please Check
PLAT083_ALERT_2_G SHELXL Second Parameter in WGHT  Unusually Large     255.79 Why ?
PLAT142_ALERT_4_G s.u. on b - Axis Small or Missing .............    0.00050 Ang.
PLAT143_ALERT_4_G s.u. on c - Axis Small or Missing .............    0.00009 Ang.
```

```
PLAT172_ALERT_4_G The CIF-Embedded .res File Contains DFIX Records          8 Report
PLAT174_ALERT_4_G The CIF-Embedded .res File Contains FLAT Records         18 Report
PLAT178_ALERT_4_G The CIF-Embedded .res File Contains SIMU Records          2 Report
PLAT186_ALERT_4_G The CIF-Embedded .res File Contains ISOR Records          9 Report
PLAT187_ALERT_4_G The CIF-Embedded .res File Contains RIGU Records         11 Report
PLAT188_ALERT_3_G A Non-default SIMU Restraint Value has been used     0.0200 Report
PLAT190_ALERT_3_G A Non-default RIGU Restraint Value for First Par     0.0020 Report
PLAT190_ALERT_3_G A Non-default RIGU Restraint Value for SecondPar     0.0020 Report
PLAT190_ALERT_3_G A Non-default RIGU Restraint Value for First Par     0.0020 Report
PLAT190_ALERT_3_G A Non-default RIGU Restraint Value for SecondPar     0.0020 Report
PLAT190_ALERT_3_G A Non-default RIGU Restraint Value for First Par     0.0020 Report
PLAT190_ALERT_3_G A Non-default RIGU Restraint Value for SecondPar     0.0020 Report
PLAT190_ALERT_3_G A Non-default RIGU Restraint Value for First Par     0.0020 Report
PLAT190_ALERT_3_G A Non-default RIGU Restraint Value for SecondPar     0.0020 Report
PLAT190_ALERT_3_G A Non-default RIGU Restraint Value for First Par     0.0020 Report
PLAT190_ALERT_3_G A Non-default RIGU Restraint Value for SecondPar     0.0020 Report
PLAT190_ALERT_3_G A Non-default RIGU Restraint Value for First Par     0.0020 Report
PLAT190_ALERT_3_G A Non-default RIGU Restraint Value for SecondPar     0.0020 Report
PLAT190_ALERT_3_G A Non-default RIGU Restraint Value for First Par     0.0020 Report
PLAT190_ALERT_3_G A Non-default RIGU Restraint Value for SecondPar     0.0020 Report
PLAT190_ALERT_3_G A Non-default RIGU Restraint Value for First Par     0.0020 Report
PLAT190_ALERT_3_G A Non-default RIGU Restraint Value for SecondPar     0.0020 Report
PLAT190_ALERT_3_G A Non-default RIGU Restraint Value for First Par     0.0020 Report
PLAT190_ALERT_3_G A Non-default RIGU Restraint Value for SecondPar     0.0020 Report
PLAT190_ALERT_3_G A Non-default RIGU Restraint Value for First Par     0.0020 Report
PLAT190_ALERT_3_G A Non-default RIGU Restraint Value for SecondPar     0.0020 Report
PLAT232_ALERT_2_G Hirshfeld Test Diff (M-X)  Pd2      --N3       .        5.2 s.u.
PLAT300_ALERT_4_G Atom Site Occupancy of N3S       Constrained at         0.5 Check
PLAT300_ALERT_4_G Atom Site Occupancy of C5S       Constrained at         0.5 Check
PLAT300_ALERT_4_G Atom Site Occupancy of C6S       Constrained at         0.5 Check
PLAT300_ALERT_4_G Atom Site Occupancy of H6SA      Constrained at         0.5 Check
PLAT300_ALERT_4_G Atom Site Occupancy of H6SB      Constrained at         0.5 Check
PLAT300_ALERT_4_G Atom Site Occupancy of H6SC      Constrained at         0.5 Check
PLAT300_ALERT_4_G Atom Site Occupancy of N4S       Constrained at         0.5 Check
PLAT300_ALERT_4_G Atom Site Occupancy of C7S       Constrained at         0.5 Check
PLAT300_ALERT_4_G Atom Site Occupancy of C8S       Constrained at         0.5 Check
PLAT300_ALERT_4_G Atom Site Occupancy of H8SA      Constrained at         0.5 Check
PLAT300_ALERT_4_G Atom Site Occupancy of H8SB      Constrained at         0.5 Check
PLAT300_ALERT_4_G Atom Site Occupancy of H8SC      Constrained at         0.5 Check
PLAT300_ALERT_4_G Atom Site Occupancy of N7S       Constrained at        0.75 Check
PLAT300_ALERT_4_G Atom Site Occupancy of C13S      Constrained at        0.75 Check
PLAT300_ALERT_4_G Atom Site Occupancy of C14S      Constrained at        0.75 Check
PLAT300_ALERT_4_G Atom Site Occupancy of H14C      Constrained at        0.75 Check
PLAT300_ALERT_4_G Atom Site Occupancy of H14D      Constrained at        0.75 Check
PLAT300_ALERT_4_G Atom Site Occupancy of H14E      Constrained at        0.75 Check
PLAT300_ALERT_4_G Atom Site Occupancy of N6S       Constrained at      0.3333 Check
PLAT300_ALERT_4_G Atom Site Occupancy of C11S      Constrained at      0.3333 Check
PLAT300_ALERT_4_G Atom Site Occupancy of C12S      Constrained at      0.3333 Check
PLAT300_ALERT_4_G Atom Site Occupancy of H12B      Constrained at      0.3333 Check
PLAT300_ALERT_4_G Atom Site Occupancy of H12C      Constrained at      0.3333 Check
PLAT300_ALERT_4_G Atom Site Occupancy of H12D      Constrained at      0.3333 Check
PLAT300_ALERT_4_G Atom Site Occupancy of O2W       Constrained at        0.75 Check
PLAT300_ALERT_4_G Atom Site Occupancy of O3W       Constrained at         0.5 Check
PLAT300_ALERT_4_G Atom Site Occupancy of O4W       Constrained at         0.5 Check
PLAT300_ALERT_4_G Atom Site Occupancy of O6W       Constrained at        0.25 Check
PLAT302_ALERT_4_G Anion/Solvent/Minor-Residue Disorder (Resd  3  )       100% Note
PLAT302_ALERT_4_G Anion/Solvent/Minor-Residue Disorder (Resd  4  )       100% Note
```

```
PLAT302_ALERT_4_G Anion/Solvent/Minor-Residue Disorder (Resd  5  )       100% Note
PLAT302_ALERT_4_G Anion/Solvent/Minor-Residue Disorder (Resd  8  )       100% Note
PLAT302_ALERT_4_G Anion/Solvent/Minor-Residue Disorder (Resd  9  )       100% Note
PLAT302_ALERT_4_G Anion/Solvent/Minor-Residue Disorder (Resd 10  )       100% Note
PLAT302_ALERT_4_G Anion/Solvent/Minor-Residue Disorder (Resd 11  )       100% Note
PLAT302_ALERT_4_G Anion/Solvent/Minor-Residue Disorder (Resd 12  )       100% Note
PLAT302_ALERT_4_G Anion/Solvent/Minor-Residue Disorder (Resd 14  )       100% Note
PLAT302_ALERT_4_G Anion/Solvent/Minor-Residue Disorder (Resd 15  )       100% Note
PLAT302_ALERT_4_G Anion/Solvent/Minor-Residue Disorder (Resd 16  )       100% Note
PLAT302_ALERT_4_G Anion/Solvent/Minor-Residue Disorder (Resd 17  )       100% Note
PLAT302_ALERT_4_G Anion/Solvent/Minor-Residue Disorder (Resd 18  )       100% Note
PLAT304_ALERT_4_G Non-Integer Number of Atoms in ..... (Resd  3  )       4.84 Check
PLAT304_ALERT_4_G Non-Integer Number of Atoms in ..... (Resd  4  )       4.35 Check
PLAT304_ALERT_4_G Non-Integer Number of Atoms in ..... (Resd  5  )       1.98 Check
PLAT304_ALERT_4_G Non-Integer Number of Atoms in ..... (Resd 10  )       3.48 Check
PLAT304_ALERT_4_G Non-Integer Number of Atoms in ..... (Resd 11  )       4.50 Check
PLAT304_ALERT_4_G Non-Integer Number of Atoms in ..... (Resd 14  )       0.75 Check
PLAT304_ALERT_4_G Non-Integer Number of Atoms in ..... (Resd 15  )       0.50 Check
PLAT304_ALERT_4_G Non-Integer Number of Atoms in ..... (Resd 16  )       0.50 Check
PLAT304_ALERT_4_G Non-Integer Number of Atoms in ..... (Resd 17  )       0.42 Check
PLAT304_ALERT_4_G Non-Integer Number of Atoms in ..... (Resd 18  )       0.25 Check
PLAT311_ALERT_2_G Isolated Disordered Oxygen Atom (No H's ?) .....        O2W Check
PLAT311_ALERT_2_G Isolated Disordered Oxygen Atom (No H's ?) .....        O3W Check
PLAT311_ALERT_2_G Isolated Disordered Oxygen Atom (No H's ?) .....        O4W Check
PLAT311_ALERT_2_G Isolated Disordered Oxygen Atom (No H's ?) .....        O5W Check
PLAT311_ALERT_2_G Isolated Disordered Oxygen Atom (No H's ?) .....        O6W Check
PLAT398_ALERT_2_G Deviating  C-O-C   Angle From 120 for O1A      .      107.8 Degree
PLAT432_ALERT_2_G Short Inter X...Y Contact  Cl11     ..C61      .       3.24 Ang.
                                             x,1/2-y,-1/2+z  =      4_565 Check
PLAT432_ALERT_2_G Short Inter X...Y Contact  O4W      ..C4C      .       2.98 Ang.
                                                    x,y,1+z  =      1_556 Check
PLAT432_ALERT_2_G Short Inter X...Y Contact  O4W      ..C3C      .       3.00 Ang.
                                                    x,y,1+z  =      1_556 Check
PLAT432_ALERT_2_G Short Inter X...Y Contact  C96      ..C5C      .       3.16 Ang.
                                                      x,y,z  =      1_555 Check
PLAT720_ALERT_4_G Number of Unusual/Non-Standard Labels .........         15 Note
PLAT790_ALERT_4_G Centre of Gravity not Within Unit Cell: Resd.  #          3 Note
              C6 H O
PLAT790_ALERT_4_G Centre of Gravity not Within Unit Cell: Resd.  #          5 Note
              C5
PLAT790_ALERT_4_G Centre of Gravity not Within Unit Cell: Resd.  #          6 Note
              C2 H3 N
PLAT793_ALERT_4_G Model has Chirality at N1          (Centro SPGR)          S Verify
PLAT793_ALERT_4_G Model has Chirality at N2          (Centro SPGR)          R Verify
PLAT793_ALERT_4_G Model has Chirality at N3          (Centro SPGR)          S Verify
PLAT793_ALERT_4_G Model has Chirality at N4          (Centro SPGR)          R Verify
PLAT793_ALERT_4_G Model has Chirality at N5          (Centro SPGR)          S Verify
PLAT793_ALERT_4_G Model has Chirality at N6          (Centro SPGR)          R Verify
PLAT793_ALERT_4_G Model has Chirality at N7          (Centro SPGR)          R Verify
PLAT793_ALERT_4_G Model has Chirality at N8          (Centro SPGR)          S Verify
PLAT793_ALERT_4_G Model has Chirality at N9          (Centro SPGR)          S Verify
PLAT793_ALERT_4_G Model has Chirality at N10         (Centro SPGR)          R Verify
PLAT793_ALERT_4_G Model has Chirality at N11         (Centro SPGR)          R Verify
PLAT793_ALERT_4_G Model has Chirality at N12         (Centro SPGR)          S Verify
PLAT794_ALERT_5_G Tentative Bond Valency for Pd1       (II)      .       2.04 Info
PLAT794_ALERT_5_G Tentative Bond Valency for Pd2       (II)      .       2.15 Info
PLAT794_ALERT_5_G Tentative Bond Valency for Pd3       (II)      .       2.08 Info
```

```
PLAT794_ALERT_5_G Tentative Bond Valency for Pd5       (II)      .       2.11 Info
PLAT794_ALERT_5_G Tentative Bond Valency for Pd6       (II)      .       2.28 Info
PLAT802_ALERT_4_G CIF Input Record(s) with more than 80 Characters          1 Info
PLAT860_ALERT_3_G Number of Least-Squares Restraints ............         500 Note
PLAT883_ALERT_1_G No Info/Value for _atom_sites_solution_primary .     Please Do !
PLAT910_ALERT_3_G Missing # of FCF Reflection(s) Below Theta(Min).          3 Note
PLAT912_ALERT_4_G Missing # of FCF Reflections Above STh/L=  0.600         10 Note
PLAT913_ALERT_3_G Missing # of Very Strong Reflections in FCF ....          2 Note
PLAT933_ALERT_2_G Number of HKL-OMIT Records in Embedded .res File          7 Note
PLAT978_ALERT_2_G Number C-C Bonds with Positive Residual Density.          0 Info
```

```
  1 ALERT level A = Most likely a serious problem - resolve or explain
  6 ALERT level B = A potentially serious problem, consider carefully
 60 ALERT level C = Check. Ensure it is not caused by an omission or oversight
128 ALERT level G = General information/check it is not something unexpected

  7 ALERT type 1 CIF construction/syntax error, inconsistent or missing data
 70 ALERT type 2 Indicator that the structure model may be wrong or deficient
 30 ALERT type 3 Indicator that the structure quality may be low
 82 ALERT type 4 Improvement, methodology, query or suggestion
  6 ALERT type 5 Informative message, check
```

It is advisable to attempt to resolve as many as possible of the alerts in all categories. Often the minor alerts point to easily fixed oversights, errors and omissions in your CIF or refinement strategy, so attention to these fine details can be worthwhile. In order to resolve some of the more serious problems it may be necessary to carry out additional measurements or structure refinements. However, the purpose of your study may justify the reported deviations and the more serious of these should normally be commented upon in the discussion or experimental section of a paper or in the "special_details" fields of the CIF. checkCIF was carefully designed to identify outliers and unusual parameters, but every test has its limitations and alerts that are not important in a particular case may appear. Conversely, the absence of alerts does not guarantee there are no aspects of the results needing attention. It is up to the individual to critically assess their own results and, if necessary, seek expert advice.

Publication of your CIF in IUCr journals

A basic structural check has been run on your CIF. These basic checks will be run on all CIFs submitted for publication in IUCr journals (*Acta Crystallographica*, *Journal of Applied Crystallography*, *Journal of Synchrotron Radiation*); however, if you intend to submit to *Acta Crystallographica Section C* or *E* or *IUCrData*, you should make sure that full publication checks are run on the final version of your CIF prior to submission.

Publication of your CIF in other journals

Please refer to the *Notes for Authors* of the relevant journal for any special instructions relating to CIF submission.

PLATON version of 28/11/2022; check.def file version of 28/11/2022

Datablock FAL-MeCN@MMF - ellipsoid plot

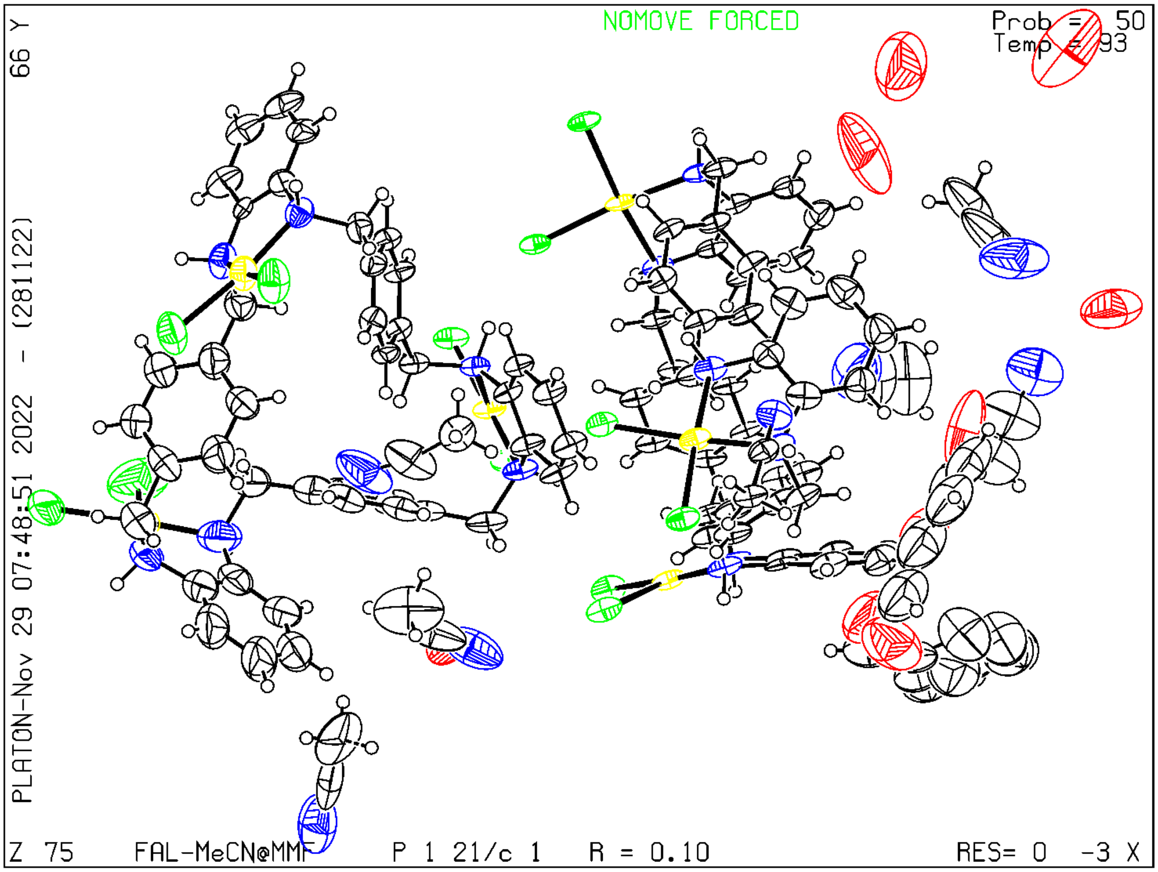

Supplement: Supplementary file 2 — Supporting Information [file SMLL-20-2405507-s001.zip › Furfural-MeCN@MMF_checkcif.pdf]
