# Supplementary material for: Porous Supramolecular Crystalline Probe that Detects Non‐Covalent Interactions Involved in Molecular Recognition of Furanic Compounds
Source: Small. 2024 Jul 30;20(49):2405507. doi: 10.1002/smll.202405507 (PMC11618713; doi:10.1002/smll.202405507)

checkCIF/PLATON report

Structure factors have been supplied for datablock(s) FOL-MeCN@MMF

THIS REPORT IS FOR GUIDANCE ONLY. IF USED AS PART OF A REVIEW PROCEDURE FOR PUBLICATION, IT SHOULD NOT REPLACE THE EXPERTISE OF AN EXPERIENCED CRYSTALLOGRAPHIC REFEREE.

No syntax errors found. CIF dictionary Interpreting this report

Datablock: FOL-MeCN@MMF

Bond precision: C-C = 0.0125 A Wavelength=1.54184

Cell:	a=19.61990(12)	b=52.2723(4)	c=14.30240(9)
	alpha=90	beta=91.5063(6)	gamma=90
Temperature:	93 K		

	Calculated	Reported
Volume	14663.15(17)	14663.15(17)
Space group	P 21/c	P 1 21/c 1
Hall group	-P 2ybc	-P 2ybc
Moiety formula	2(C42 H42 Cl6 N6 Pd3), C4.13 H4.13 O1.65, C3.44 H3.44 O1.38, 2.	2(C42 H42 Cl6 N6 Pd3), C2 H3 N, 4.727(O), 3.532(C5 H5 O2), 0.48
Sum formula	C106.06 H104.66 Cl12 N13 O11.80 Pd6	C106.08 H104.66 Cl12 N13 O11.79 Pd6
Mr	2814.05	2814.12
Dx,g cm-3	1.275	1.275
Z	4	4
Mu (mm-1)	8.225	8.225
F000	5625.8	5626.0
F000’	5658.72	
h,k,lmax	23,62,17	23,62,17
Nref	26865	26837
Tmin,Tmax	0.227,0.567	0.443,1.000
Tmin’	0.055	

Correction method= # Reported T Limits: Tmin=0.443 Tmax=1.000
AbsCorr = MULTI-SCAN

Data completeness= 0.999 Theta(max)= 68.249

R(reflections)= 0.1011(22800) wR2(reflections)= 0.2821(26837)

S = 1.093 Npar= 1463

The following ALERTS were generated. Each ALERT has the format
test-name_ALERT_alert-type_alert-level.
Click on the hyperlinks for more details of the test.

Alert level A

PLAT602_ALERT_2_A Solvent Accessible VOID(S) in Structure .... ! Check

Author Response: Some solvents in the large pore could not be located due to severe disordering.

PLAT971_ALERT_2_A Check Calcd Resid. Dens. 0.92Ang From Pd1 3.55 eA-3

Author Response: The atom type is correct and there is no evidence of twinning. The large residual density on Pd atoms may be Due to an anomalous dispersion effect and has no chemical significance.

Alert level B

PLAT430_ALERT_2_B Short Inter D...A Contact O2A ..O3D . 2.77 Ang.
2-x,1-y,-z = 3_765 Check

Author Response: The short contacts come from ROH...ROH, though hydrogen atoms of hydroxy groups could not be located in the difference electron density maps. Therefore, these contacts can be assigned to hydrogen bonding between hydroxy groups.

PLAT971_ALERT_2_B Check Calcd Resid. Dens. 0.93Ang From Pd6 3.38 eA-3

Author Response: The atom type is correct and there is no evidence of twinning. The large residual density on Pd atoms may be Due to an anomalous dispersion effect and has no chemical significance.

PLAT971_ALERT_2_B Check Calcd Resid. Dens. 0.82Ang From Pd1 3.25 eA-3

Author Response: The atom type is correct and there is no evidence of twinning. The large residual density on Pd atoms may be Due to an anomalous dispersion effect and has no chemical significance.

PLAT971_ALERT_2_B Check Calcd Resid. Dens. 1.00Ang From Pd3 3.20 eA-3

Author Response: The atom type is correct and there is no evidence of twinning. The large residual density on Pd atoms may be Due to an anomalous dispersion effect and has no chemical significance.

PLAT971_ALERT_2_B Check Calcd Resid. Dens. 0.94Ang From Pd6 3.14 eA-3

Author Response: The atom type is correct and there is no evidence of twinning. The large residual density on Pd atoms may be Due to an anomalous dispersion effect and has no chemical significance.

PLAT971_ALERT_2_B Check Calcd Resid. Dens. 0.98Ang From Pd4 3.06 eA-3

Author Response: The atom type is correct and there is no evidence of twinning. The large residual density on Pd atoms may be Due to an anomalous dispersion effect and has no chemical significance.

PLAT971_ALERT_2_B Check Calcd Resid. Dens. 0.90Ang From Pd4 3.02 eA-3

Author Response: The atom type is correct and there is no evidence of twinning. The large residual density on Pd atoms may be Due to an anomalous dispersion effect and has no chemical significance.

PLAT971_ALERT_2_B Check Calcd Resid. Dens. 1.03Ang From Pd3 2.79 eA-3

Author Response: The atom type is correct and there is no evidence of twinning. The large residual density on Pd atoms may be Due to an anomalous dispersion effect and has no chemical significance.

PLAT971_ALERT_2_B Check Calcd Resid. Dens. 0.94Ang From Pd2 2.71 eA-3

Author Response: The atom type is correct and there is no evidence of twinning. The large residual density on Pd atoms may be Due to an anomalous dispersion effect and has no chemical significance.

PLAT971_ALERT_2_B Check Calcd Resid. Dens. 1.07Ang From Pd2 2.64 eA-3

Author Response: The atom type is correct and there is no evidence of twinning. The large residual density on Pd atoms may be Due to an anomalous dispersion effect and has no chemical significance.

PLAT971_ALERT_2_B Check Calcd Resid. Dens. 0.54Ang From Pd1 2.61 eA-3

Author Response: The atom type is correct and there is no evidence of twinning. The large residual density on Pd atoms may be Due to an anomalous dispersion effect and has no chemical significance.

PLAT971_ALERT_2_B Check Calcd Resid. Dens. 0.65Ang From Pd1 2.53 eA-3

Author Response: The atom type is correct and there is no evidence of twinning. The large residual density on Pd atoms may be Due to an anomalous dispersion effect and has no chemical significance.

Alert level C

DIFMX02_ALERT_1_C The maximum difference density is > 0.1*ZMAX*0.75
The relevant atom site should be identified.
PLAT041_ALERT_1_C Calc. and Reported SumFormula Strings Differ Please Check
PLAT042_ALERT_1_C Calc. and Reported MoietyFormula Strings Differ Please Check
PLAT084_ALERT_3_C High wR2 Value (i.e. > 0.25) .... 0.28 Report
PLAT094_ALERT_2_C Ratio of Maximum / Minimum Residual Density .... 2.46 Report
PLAT097_ALERT_2_C Large Reported Max. (Positive) Residual Density 3.52 eA-3
PLAT213_ALERT_2_C Atom C20 has ADP max/min Ratio ..... 3.4 prolat
PLAT230_ALERT_2_C Hirshfeld Test Diff for C61 --C62 . 6.8 s.u.
PLAT242_ALERT_2_C Low 'MainMol' Ueq as Compared to Neighbors of Pd6 Check
PLAT250_ALERT_2_C Large U3/U1 Ratio for Average U(i,j) Tensor .... 2.9 Note
PLAT250_ALERT_2_C Large U3/U1 Ratio for Average U(i,j) Tensor .... 2.5 Note
PLAT260_ALERT_2_C Large Average Ueq of Residue Including O3D 0.198 Check
PLAT260_ALERT_2_C Large Average Ueq of Residue Including O3E 0.162 Check
PLAT260_ALERT_2_C Large Average Ueq of Residue Including O1A 0.164 Check
PLAT260_ALERT_2_C Large Average Ueq of Residue Including O1B 0.215 Check
PLAT260_ALERT_2_C Large Average Ueq of Residue Including O1C 0.179 Check
PLAT260_ALERT_2_C Large Average Ueq of Residue Including C1F 0.204 Check
PLAT260_ALERT_2_C Large Average Ueq of Residue Including O1W 0.217 Check
PLAT260_ALERT_2_C Large Average Ueq of Residue Including O2W 0.288 Check
PLAT260_ALERT_2_C Large Average Ueq of Residue Including O3W 0.271 Check
PLAT260_ALERT_2_C Large Average Ueq of Residue Including O6W 0.218 Check
PLAT260_ALERT_2_C Large Average Ueq of Residue Including O11W 0.118 Check
PLAT260_ALERT_2_C Large Average Ueq of Residue Including O4W 0.255 Check
PLAT260_ALERT_2_C Large Average Ueq of Residue Including O5W 0.241 Check
PLAT260_ALERT_2_C Large Average Ueq of Residue Including O7W 0.266 Check
PLAT260_ALERT_2_C Large Average Ueq of Residue Including O8W 0.198 Check
PLAT260_ALERT_2_C Large Average Ueq of Residue Including O9W 0.238 Check
PLAT260_ALERT_2_C Large Average Ueq of Residue Including O10W 0.107 Check
PLAT309_ALERT_2_C Single Bonded Oxygen (C-O > 1.3 Ang) .... O3D Check
PLAT309_ALERT_2_C Single Bonded Oxygen (C-O > 1.3 Ang) .... O3E Check
PLAT309_ALERT_2_C Single Bonded Oxygen (C-O > 1.3 Ang) .... O2A Check
PLAT309_ALERT_2_C Single Bonded Oxygen (C-O > 1.3 Ang) .... O2B Check
PLAT309_ALERT_2_C Single Bonded Oxygen (C-O > 1.3 Ang) .... O2C Check
PLAT334_ALERT_2_C Small <C-C> Benzene Dist. C15 -C20 . 1.36 Ang.
PLAT334_ALERT_2_C Small <C-C> Benzene Dist. C64 -C69 . 1.37 Ang.
PLAT334_ALERT_2_C Small <C-C> Benzene Dist. C71 -C76 . 1.36 Ang.
PLAT342_ALERT_3_C Low Bond Precision on C-C Bonds .... 0.01254 Ang.

```
PLAT411_ALERT_2_C Short Inter H...H Contact  H58      ..H84B     .       2.14 Ang.
                                                    x,y,1+z  =      1_556 Check
PLAT420_ALERT_2_C D-H Bond Without Acceptor  N7       --H7       .     Please Check
PLAT767_ALERT_4_C INS Embedded LIST 6 Instruction Should be LIST 4     Please Check
PLAT906_ALERT_3_C Large K Value in the Analysis of Variance ......      3.968 Check
PLAT911_ALERT_3_C Missing FCF Refl Between Thmin & STh/L=    0.600         20 Report
PLAT918_ALERT_3_C Reflection(s) with I(obs) much Smaller I(calc) .          3 Check
PLAT971_ALERT_2_C Check Calcd Resid. Dens.  0.66Ang From Pd4           2.40 eA-3
```

Author Response: The atom type is correct and there is no evidence of twinning. The large residual density on Pd atoms may be Due to an anomalous dispersion effect and has no chemical significance.

```
PLAT971_ALERT_2_C Check Calcd Resid. Dens.  0.91Ang From Pd5           2.23 eA-3
```

Author Response: The atom type is correct and there is no evidence of twinning. The large residual density on Pd atoms may be Due to an anomalous dispersion effect and has no chemical significance.

```
PLAT971_ALERT_2_C Check Calcd Resid. Dens.  0.86Ang From C62           2.20 eA-3
```

Author Response: The atom type is correct and there is no evidence of twinning. The large residual density on Pd atoms may be Due to an anomalous dispersion effect and has no chemical significance.

```
PLAT971_ALERT_2_C Check Calcd Resid. Dens.  0.74Ang From Pd2           2.11 eA-3
```

Author Response: The atom type is correct and there is no evidence of twinning. The large residual density on Pd atoms may be Due to an anomalous dispersion effect and has no chemical significance.

```
PLAT971_ALERT_2_C Check Calcd Resid. Dens.  1.09Ang From Pd5           2.08 eA-3
```

Author Response: The atom type is correct and there is no evidence of twinning. The large residual density on Pd atoms may be Due to an anomalous dispersion effect and has no chemical significance.

```
PLAT971_ALERT_2_C Check Calcd Resid. Dens.  0.88Ang From Pd5           2.05 eA-3
```

Author Response: The atom type is correct and there is no evidence of twinning. The large residual density on Pd atoms may be Due to an anomalous dispersion effect and has no chemical significance.

```
PLAT971_ALERT_2_C Check Calcd Resid. Dens.  0.80Ang From Pd2            1.89 eA-3
```

Author Response: The atom type is correct and there is no evidence of twinning. The large residual density on Pd atoms may be Due to an anomalous dispersion effect and has no chemical significance.

```
PLAT971_ALERT_2_C Check Calcd Resid. Dens.  0.70Ang From Pd3            1.84 eA-3
```

Author Response: The atom type is correct and there is no evidence of twinning. The large residual density on Pd atoms may be Due to an anomalous dispersion effect and has no chemical significance.

```
PLAT971_ALERT_2_C Check Calcd Resid. Dens.  0.59Ang From Pd4            1.73 eA-3
```

Author Response: The atom type is correct and there is no evidence of twinning. The large residual density on Pd atoms may be Due to an anomalous dispersion effect and has no chemical significance.

```
PLAT971_ALERT_2_C Check Calcd Resid. Dens.  0.70Ang From Pd3            1.72 eA-3
```

Author Response: The atom type is correct and there is no evidence of twinning. The large residual density on Pd atoms may be Due to an anomalous dispersion effect and has no chemical significance.

```
PLAT971_ALERT_2_C Check Calcd Resid. Dens.  0.95Ang From Pd5            1.64 eA-3
```

Author Response: The atom type is correct and there is no evidence of twinning. The large residual density on Pd atoms may be Due to an anomalous dispersion effect and has no chemical significance.

```
PLAT977_ALERT_2_C Check Negative Difference Density on H3AA      .      -0.34 eA-3
PLAT977_ALERT_2_C Check Negative Difference Density on H5BA      .      -0.47 eA-3
PLAT977_ALERT_2_C Check Negative Difference Density on H1D       .      -0.56 eA-3
PLAT977_ALERT_2_C Check Negative Difference Density on H2E       .      -0.44 eA-3
PLAT977_ALERT_2_C Check Negative Difference Density on H2EA      .      -0.44 eA-3
PLAT977_ALERT_2_C Check Negative Difference Density on H4E       .      -0.34 eA-3
```

Alert level G

```
FORMU01_ALERT_2_G  There is a discrepancy between the atom counts in the
            _chemical_formula_sum and the formula from the _atom_site* data.
            Atom count from _chemical_formula_sum:C106.08 H104.66 Cl12 N13 O11.79
            Atom count from the _atom_site data:  C106.0600 H104.6600 Cl12 N13 O11
CELLZ01_ALERT_1_G Difference between formula and atom_site contents detected.
CELLZ01_ALERT_1_G ALERT: check formula stoichiometry or atom site occupancies.
           From the CIF: _cell_formula_units_Z    4
           From the CIF: _chemical_formula_sum  C106.08 H104.66 Cl12 N13 O11.79 Pd
           TEST: Compare cell contents of formula and atom_site data
```

```
atom    Z*formula  cif sites diff
C        424.32    424.24    0.08
H        418.64    418.64   -0.00
Cl        48.00     48.00    0.00
N         52.00     52.00    0.00
O         47.16     47.21   -0.05
Pd        24.00     24.00    0.00
PLAT002_ALERT_2_G Number of Distance or Angle Restraints on AtSite         23 Note
PLAT003_ALERT_2_G Number of Uiso or Uij Restrained non-H Atoms ...         49 Report
PLAT007_ALERT_5_G Number of Unrefined Donor-H Atoms .............          12 Report
PLAT068_ALERT_1_G Reported F000 Differs from Calcd (or Missing)...     Please Check
PLAT072_ALERT_2_G SHELXL First  Parameter in WGHT  Unusually Large       0.19 Report
PLAT083_ALERT_2_G SHELXL Second Parameter in WGHT  Unusually Large      34.25 Why ?
PLAT142_ALERT_4_G s.u. on b - Axis Small or Missing .............     0.00040 Ang.
PLAT143_ALERT_4_G s.u. on c - Axis Small or Missing .............     0.00009 Ang.
PLAT168_ALERT_4_G The CIF-Embedded .res File Contains EXYZ Records          4 Report
PLAT171_ALERT_4_G The CIF-Embedded .res File Contains EADP Records          4 Report
PLAT172_ALERT_4_G The CIF-Embedded .res File Contains DFIX Records         18 Report
PLAT174_ALERT_4_G The CIF-Embedded .res File Contains FLAT Records          1 Report
PLAT178_ALERT_4_G The CIF-Embedded .res File Contains SIMU Records          9 Report
PLAT186_ALERT_4_G The CIF-Embedded .res File Contains ISOR Records          9 Report
PLAT187_ALERT_4_G The CIF-Embedded .res File Contains RIGU Records         10 Report
PLAT190_ALERT_3_G A Non-default RIGU Restraint Value for First Par     0.0020 Report
PLAT190_ALERT_3_G A Non-default RIGU Restraint Value for SecondPar     0.0020 Report
PLAT232_ALERT_2_G Hirshfeld Test Diff (M-X)  Pd6      --N11      .        5.3 s.u.
PLAT300_ALERT_4_G Atom Site Occupancy of O6W       Constrained at         0.5 Check
PLAT300_ALERT_4_G Atom Site Occupancy of O4W       Constrained at      0.3333 Check
PLAT300_ALERT_4_G Atom Site Occupancy of O7W       Constrained at      0.3333 Check
PLAT300_ALERT_4_G Atom Site Occupancy of O8W       Constrained at      0.3333 Check
PLAT300_ALERT_4_G Atom Site Occupancy of O9W       Constrained at      0.3333 Check
PLAT302_ALERT_4_G Anion/Solvent/Minor-Residue Disorder (Resd  3  )       100% Note
PLAT302_ALERT_4_G Anion/Solvent/Minor-Residue Disorder (Resd  4  )       100% Note
PLAT302_ALERT_4_G Anion/Solvent/Minor-Residue Disorder (Resd  5  )       100% Note
PLAT302_ALERT_4_G Anion/Solvent/Minor-Residue Disorder (Resd  6  )       100% Note
PLAT302_ALERT_4_G Anion/Solvent/Minor-Residue Disorder (Resd  7  )       100% Note
PLAT302_ALERT_4_G Anion/Solvent/Minor-Residue Disorder (Resd  8  )       100% Note
PLAT302_ALERT_4_G Anion/Solvent/Minor-Residue Disorder (Resd 10  )       100% Note
PLAT302_ALERT_4_G Anion/Solvent/Minor-Residue Disorder (Resd 11  )       100% Note
PLAT302_ALERT_4_G Anion/Solvent/Minor-Residue Disorder (Resd 12  )       100% Note
PLAT302_ALERT_4_G Anion/Solvent/Minor-Residue Disorder (Resd 13  )       100% Note
PLAT302_ALERT_4_G Anion/Solvent/Minor-Residue Disorder (Resd 14  )       100% Note
PLAT302_ALERT_4_G Anion/Solvent/Minor-Residue Disorder (Resd 15  )       100% Note
PLAT302_ALERT_4_G Anion/Solvent/Minor-Residue Disorder (Resd 16  )       100% Note
PLAT302_ALERT_4_G Anion/Solvent/Minor-Residue Disorder (Resd 17  )       100% Note
PLAT302_ALERT_4_G Anion/Solvent/Minor-Residue Disorder (Resd 18  )       100% Note
PLAT302_ALERT_4_G Anion/Solvent/Minor-Residue Disorder (Resd 19  )       100% Note
PLAT302_ALERT_4_G Anion/Solvent/Minor-Residue Disorder (Resd 20  )       100% Note
PLAT304_ALERT_4_G Non-Integer Number of Atoms in ..... (Resd  3  )       9.91 Check
PLAT304_ALERT_4_G Non-Integer Number of Atoms in ..... (Resd  4  )       8.26 Check
PLAT304_ALERT_4_G Non-Integer Number of Atoms in ..... (Resd  5  )       9.35 Check
PLAT304_ALERT_4_G Non-Integer Number of Atoms in ..... (Resd  6  )       7.86 Check
PLAT304_ALERT_4_G Non-Integer Number of Atoms in ..... (Resd  8  )       2.40 Check
PLAT304_ALERT_4_G Non-Integer Number of Atoms in ..... (Resd 10  )       0.52 Check
PLAT304_ALERT_4_G Non-Integer Number of Atoms in ..... (Resd 11  )       0.52 Check
PLAT304_ALERT_4_G Non-Integer Number of Atoms in ..... (Resd 12  )       0.52 Check
PLAT304_ALERT_4_G Non-Integer Number of Atoms in ..... (Resd 13  )       0.50 Check
```

```
PLAT304_ALERT_4_G Non-Integer Number of Atoms in ..... (Resd 14  )       0.78 Check
PLAT304_ALERT_4_G Non-Integer Number of Atoms in ..... (Resd 15  )       0.33 Check
PLAT304_ALERT_4_G Non-Integer Number of Atoms in ..... (Resd 16  )       0.34 Check
PLAT304_ALERT_4_G Non-Integer Number of Atoms in ..... (Resd 17  )       0.33 Check
PLAT304_ALERT_4_G Non-Integer Number of Atoms in ..... (Resd 18  )       0.33 Check
PLAT304_ALERT_4_G Non-Integer Number of Atoms in ..... (Resd 19  )       0.33 Check
PLAT304_ALERT_4_G Non-Integer Number of Atoms in ..... (Resd 20  )       0.22 Check
PLAT311_ALERT_2_G Isolated Disordered Oxygen Atom (No H's ?) .....        O1W Check
PLAT311_ALERT_2_G Isolated Disordered Oxygen Atom (No H's ?) .....        O2W Check
PLAT311_ALERT_2_G Isolated Disordered Oxygen Atom (No H's ?) .....        O3W Check
PLAT311_ALERT_2_G Isolated Disordered Oxygen Atom (No H's ?) .....        O6W Check
PLAT311_ALERT_2_G Isolated Disordered Oxygen Atom (No H's ?) .....       O11W Check
PLAT311_ALERT_2_G Isolated Disordered Oxygen Atom (No H's ?) .....        O4W Check
PLAT311_ALERT_2_G Isolated Disordered Oxygen Atom (No H's ?) .....        O5W Check
PLAT311_ALERT_2_G Isolated Disordered Oxygen Atom (No H's ?) .....        O7W Check
PLAT311_ALERT_2_G Isolated Disordered Oxygen Atom (No H's ?) .....        O8W Check
PLAT311_ALERT_2_G Isolated Disordered Oxygen Atom (No H's ?) .....        O9W Check
PLAT311_ALERT_2_G Isolated Disordered Oxygen Atom (No H's ?) .....       O10W Check
PLAT335_ALERT_2_G Check Large C6 Ring C-C Range C29      -C34            0.16 Ang.
PLAT335_ALERT_2_G Check Large C6 Ring C-C Range C71      -C76            0.19 Ang.
PLAT398_ALERT_2_G Deviating  C-O-C   Angle From 120 for O1D      .      108.0 Degree
PLAT398_ALERT_2_G Deviating  C-O-C   Angle From 120 for O2D      .      107.9 Degree
PLAT398_ALERT_2_G Deviating  C-O-C   Angle From 120 for O1E      .      108.1 Degree
PLAT398_ALERT_2_G Deviating  C-O-C   Angle From 120 for O2E      .      108.0 Degree
PLAT398_ALERT_2_G Deviating  C-O-C   Angle From 120 for O1A      .      108.1 Degree
PLAT398_ALERT_2_G Deviating  C-O-C   Angle From 120 for O1B      .      108.1 Degree
PLAT432_ALERT_2_G Short Inter X...Y Contact  O2A      ..C6D      .       3.00 Ang.
                                              2-x,1-y,-z  =      3_765 Check
PLAT432_ALERT_2_G Short Inter X...Y Contact  C1C      ..C18      .       3.18 Ang.
                                                 x,y,1+z  =      1_556 Check
PLAT720_ALERT_4_G Number of Unusual/Non-Standard Labels .........          19 Note
PLAT790_ALERT_4_G Centre of Gravity not Within Unit Cell: Resd.  #         16 Note
              O
PLAT793_ALERT_4_G Model has Chirality at N1          (Centro SPGR)          R Verify
PLAT793_ALERT_4_G Model has Chirality at N2          (Centro SPGR)          S Verify
PLAT793_ALERT_4_G Model has Chirality at N3          (Centro SPGR)          R Verify
PLAT793_ALERT_4_G Model has Chirality at N4          (Centro SPGR)          S Verify
PLAT793_ALERT_4_G Model has Chirality at N5          (Centro SPGR)          R Verify
PLAT793_ALERT_4_G Model has Chirality at N6          (Centro SPGR)          S Verify
PLAT793_ALERT_4_G Model has Chirality at N7          (Centro SPGR)          S Verify
PLAT793_ALERT_4_G Model has Chirality at N8          (Centro SPGR)          R Verify
PLAT793_ALERT_4_G Model has Chirality at N9          (Centro SPGR)          R Verify
PLAT793_ALERT_4_G Model has Chirality at N10         (Centro SPGR)          S Verify
PLAT793_ALERT_4_G Model has Chirality at N11         (Centro SPGR)          S Verify
PLAT793_ALERT_4_G Model has Chirality at N12         (Centro SPGR)          R Verify
PLAT794_ALERT_5_G Tentative Bond Valency for Pd1       (II)      .       2.02 Info
PLAT794_ALERT_5_G Tentative Bond Valency for Pd2       (II)      .       2.06 Info
PLAT794_ALERT_5_G Tentative Bond Valency for Pd3       (II)      .       2.06 Info
PLAT794_ALERT_5_G Tentative Bond Valency for Pd4       (II)      .       2.05 Info
PLAT794_ALERT_5_G Tentative Bond Valency for Pd5       (II)      .       2.08 Info
PLAT794_ALERT_5_G Tentative Bond Valency for Pd6       (II)      .       2.04 Info
PLAT802_ALERT_4_G CIF Input Record(s) with more than 80 Characters          2 Info
PLAT860_ALERT_3_G Number of Least-Squares Restraints ............         677 Note
PLAT883_ALERT_1_G No Info/Value for _atom_sites_solution_primary .     Please Do !
PLAT910_ALERT_3_G Missing # of FCF Reflection(s) Below Theta(Min).          4 Note
PLAT912_ALERT_4_G Missing # of FCF Reflections Above STh/L=  0.600          5 Note
PLAT913_ALERT_3_G Missing # of Very Strong Reflections in FCF ....          2 Note
```

```
PLAT933_ALERT_2_G Number of HKL-OMIT Records in Embedded .res File         10 Note
PLAT978_ALERT_2_G Number C-C Bonds with Positive Residual Density.          0 Info
```

```
  2 ALERT level A = Most likely a serious problem - resolve or explain
 12 ALERT level B = A potentially serious problem, consider carefully
 60 ALERT level C = Check. Ensure it is not caused by an omission or oversight
108 ALERT level G = General information/check it is not something unexpected

  7 ALERT type 1 CIF construction/syntax error, inconsistent or missing data
 94 ALERT type 2 Indicator that the structure model may be wrong or deficient
 10 ALERT type 3 Indicator that the structure quality may be low
 64 ALERT type 4 Improvement, methodology, query or suggestion
  7 ALERT type 5 Informative message, check
```

It is advisable to attempt to resolve as many as possible of the alerts in all categories. Often the minor alerts point to easily fixed oversights, errors and omissions in your CIF or refinement strategy, so attention to these fine details can be worthwhile. In order to resolve some of the more serious problems it may be necessary to carry out additional measurements or structure refinements. However, the purpose of your study may justify the reported deviations and the more serious of these should normally be commented upon in the discussion or experimental section of a paper or in the "special_details" fields of the CIF. checkCIF was carefully designed to identify outliers and unusual parameters, but every test has its limitations and alerts that are not important in a particular case may appear. Conversely, the absence of alerts does not guarantee there are no aspects of the results needing attention. It is up to the individual to critically assess their own results and, if necessary, seek expert advice.

Publication of your CIF in IUCr journals

A basic structural check has been run on your CIF. These basic checks will be run on all CIFs submitted for publication in IUCr journals (*Acta Crystallographica*, *Journal of Applied Crystallography*, *Journal of Synchrotron Radiation*); however, if you intend to submit to *Acta Crystallographica Section C* or *E* or *IUCrData*, you should make sure that full publication checks are run on the final version of your CIF prior to submission.

Publication of your CIF in other journals

Please refer to the *Notes for Authors* of the relevant journal for any special instructions relating to CIF submission.

PLATON version of 28/11/2022; check.def file version of 28/11/2022

Datablock FOL-MeCN@MMF - ellipsoid plot

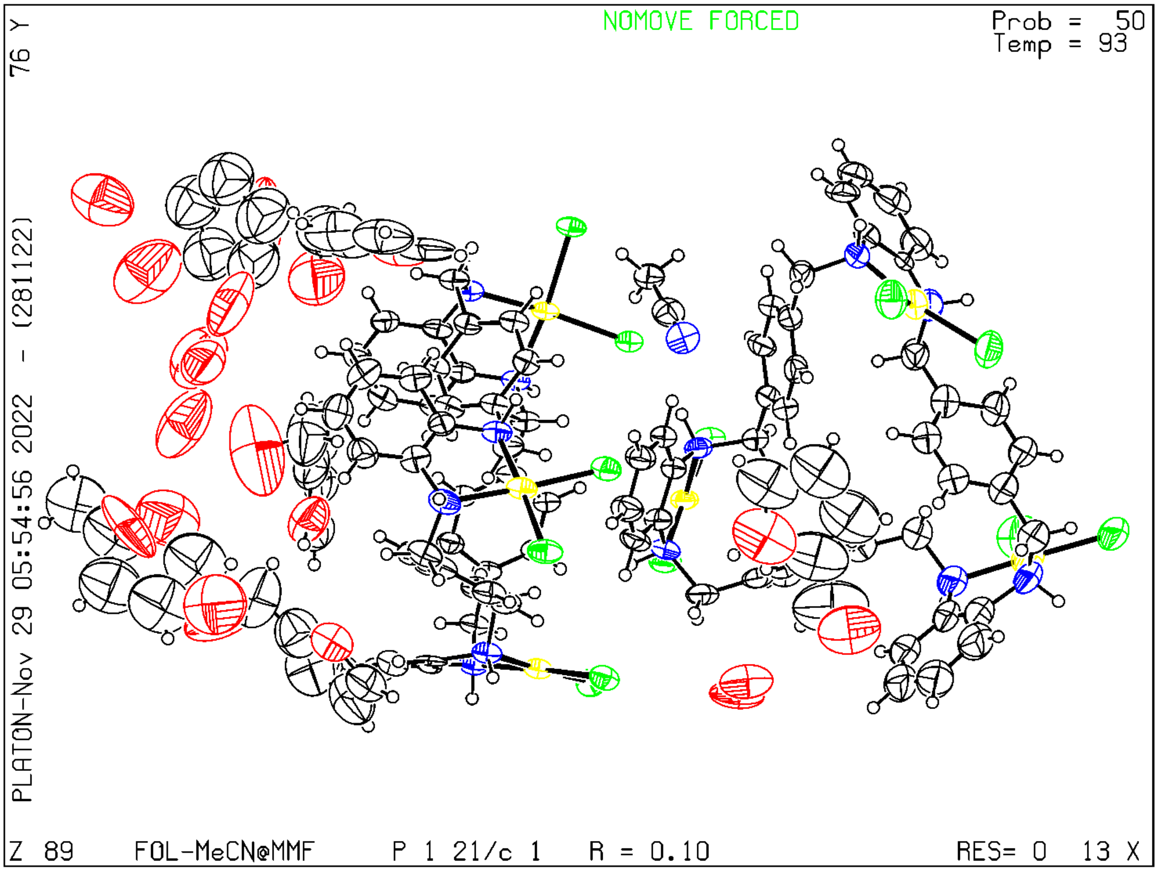

Supplement: Supplementary file 2 — Supporting Information [file SMLL-20-2405507-s001.zip › Furfurylalcohol-MeCN@MMF_checkcif.pdf]
